# RGI‐GOLVEN signaling promotes cell surface immune receptor abundance to regulate plant immunity

**DOI:** 10.15252/embr.202153281

**Published:** 2022-03-01

**Authors:** Martin Stegmann, Patricia Zecua‐Ramirez, Christina Ludwig, Ho‐Seok Lee, Brenda Peterson, Zachary L Nimchuk, Youssef Belkhadir, Ralph Hückelhoven

**Affiliations:** ^1^ Phytopathology School of Life Sciences Technical University of Munich Freising Germany; ^2^ Bavarian Center for Biomolecular Mass Spectrometry (BayBioMS) Technical University of Munich Freising Germany; ^3^ Gregor Mendel Institute (GMI) Austrian Academy of Sciences Vienna Biocenter (VBC) Vienna Austria; ^4^ Department of Biology University of North Carolina at Chapel Hill Chapel Hill NC USA; ^5^ Present address: Institute for Plant Sciences University of Cologne Cologne Germany; ^6^ Present address: Department of Biology Kyung Hee University Seoul Republic of Korea

**Keywords:** pattern‐triggered immunity, phytocytokines, plant endogenous peptides, receptor kinases, Immunology, Microbiology, Virology & Host Pathogen Interaction, Signal Transduction

## Abstract

Plant immune responses must be tightly controlled for proper allocation of resources for growth and development. In plants, endogenous signaling peptides regulate developmental and growth‐related processes. Recent research indicates that some of these peptides also have regulatory functions in the control of plant immune responses. This classifies these peptides as phytocytokines as they show analogies with metazoan cytokines. However, the mechanistic basis for phytocytokine‐mediated regulation of plant immunity remains largely elusive. Here, we identify GOLVEN2 (GLV2) peptides as phytocytokines in *Arabidopsis thaliana*. GLV2 signaling enhances sensitivity of plants to elicitation with immunogenic bacterial elicitors and contributes to resistance against virulent bacterial pathogens. GLV2 is perceived by ROOT MERISTEM GROWTH FACTOR 1 INSENSITIVE (RGI) receptors. *RGI* mutants show reduced elicitor sensitivity and enhanced susceptibility to bacterial infection. RGI3 forms ligand‐induced complexes with the pattern recognition receptor (PRR) FLAGELLIN SENSITIVE 2 (FLS2), suggesting that RGIs are part of PRR signaling platforms. GLV2‐RGI signaling promotes PRR abundance independent of transcriptional regulation and controls plant immunity via a previously undescribed mechanism of phytocytokine activity.

## Graphical abstract

GLV peptides are phytocytokines modulating pattern‐triggered immunity in *Arabidopsis*. The GLV2 receptor RGI3 interacts with the pattern recognition receptor FLS2 and GLV2 perception promotes cell surface immune receptor abundance to control anti‐bacterial immunity.

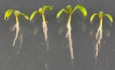

## Introduction

Receptor kinases (RKs) sense external and internal cues, including plant peptide hormones, to regulate a diverse range of plant physiological responses, ranging from growth/development to plant immunity and stress responses. RKs can serve as pattern recognition receptors (PRRs) for detecting microbe‐associated molecular patterns (MAMPs) to trigger pattern‐triggered immunity (PTI), the first line of induced defense of plants against invading microbes. Well‐characterized examples are the perception of bacterial flagellin and ELONGATION FACTOR THERMO‐UNSTABLE (EF‐TU) by the leucine‐rich repeat (LRR)‐RKs FLAGELLIN‐SENSITIVE 2 (FLS2) and EF‐TU RECEPTOR (EFR), respectively (Gómez‐Gómez & Boller, 2000; Kunze *et al*, 2004; Zipfel *et al*, 2004, 2006). FLS2 binds to a 22 amino acid peptide derived from bacterial flagellin (flg22) which is released by host hydrolytic enzymes (Buscaill *et al*, 2019). EFR perceives a 18 amino acid peptide within EF‐TU (elf18) (Kunze *et al*, 2004). Perception of flg22 and elf18 results in the activation of downstream signaling culminating in basal resistance against pathogens (Couto & Zipfel, 2016; DeFalco & Zipfel, 2021).

In addition to MAMPs, plants can also perceive endogenous peptides to sense danger and modulate immune signaling by PRRs. Research in recent years has identified several classes of such immunomodulatory peptides. These are secreted and modulate cell‐to‐cell signaling. They are functionally analogous to metazoan cytokines and thus referred to as phytocytokines (Gust *et al*, 2017). Importantly, mammalian cytokine and MAMP signaling share downstream pathways and regulate each other. However, the modulatory mechanisms of phytocytokines on plant immune responses remain for the most part poorly characterized (Trinchieri & Sher, 2007; Gust *et al*, 2017). Among the most prominent plant immune‐amplifying peptides are the PLANT ELICITOR PEPTIDES (PEPs) which derive from the C terminus of PROPEP precursors and are proteolytically released by METACASPASES (MCs) including MC4 (Bartels *et al*, 2013; Hander *et al*, 2019). *PROPEP* gene expression is triggered by biotic stresses, including microbial infection and MAMP perception (Bartels & Boller, 2015). Two additional peptides that work in a similar fashion are PATHOGEN‐ASSOCIATED MOLECULAR PATTERN (PAMP)‐INDUCED PEPTIDES (PIPs) and SERINE‐RICH ENDOGENOUS PEPTIDES (SCOOPs), which are perceived by RECEPTOR‐LIKE KINASE 7 (RLK7) and MALE DISCOVERER 1‐INTERACTING RECEPTOR‐LIKE KINASE 2 (MIK2), respectively (Hou *et al*, 2014, 2021; Gully *et al*, 2019; Rhodes *et al*, 2021). PEPs, PIPs, and SCOOPs are also implicated in positive feedback loops to boost immune stimulation during infections. Together with their cognate receptors, several peptides that are mainly associated with growth regulatory functions are also involved in controlling plant immune responses. These include RAPID ALKALINIZATION FACTORS (RALFs), PHYTOSULFOKINE (PSK) and PLANT PEPTIDE‐CONTAINING SULFATED TYROSINE 1 (PSY1) (Igarashi *et al*, 2012; Mosher *et al*, 2013; Shen *et al*, 2017; Stegmann *et al*, 2017; Xiao *et al*, 2019). RALF peptide perception through a FERONIA (FER)–LORELEI‐like GPI‐ANCHORED PROTEIN 1 (LLG1) heterocomplex controls PTI through the regulation of PRR complex assembly at the plasma membrane (Stegmann *et al*, 2017; Xiao *et al*, 2019). However, the molecular mechanism of signaling crosstalk between other peptide pathways and PTI remains largely unknown (Segonzac & Monaghan, 2019).

GOLVEN (GLV) peptides, which are also known as ROOT MERISTEM GROWTH FACTOR (RGF) or CLAVATA 3/EMBRYO SURROUNDING REGION‐LIKE (CLEL) peptides, are encoded by a family of 11 genes in *Arabidopsis* (Fernandez *et al*, 2013a). The numbering of the genes is different depending on the used nomenclature (Appendix Table S1) (Fernandez *et al*, 2013a). Hereafter, we will refer to these peptides as GLVs. They are produced as preproproteins and require proteolytic cleavage steps for maturation and extracellular release of the fully maturated 13‐ to 18‐amino‐acid‐long peptide (Ghorbani *et al*, 2016; Stührwohldt *et al*, 2020). For full activity, GLVs are sulfated on a conserved tyrosine residue targeted by TYROSYLPROTEIN SULFOTRANSFERASE (TPST) (Matsuzaki *et al*, 2010; Whitford *et al*, 2012). GLVs play multiple roles on root development and they have been associated with gravitropism, root stem cell niche maintenance, and lateral root formation (Matsuzaki *et al*, 2010; Meng *et al*, 2012; Whitford *et al*, 2012; Fernandez *et al*, 2015, 2020). Several *GLV* genes can be simultaneously expressed in individual cell types or organs, suggesting redundant functions (Matsuzaki *et al*, 2010; Whitford *et al*, 2012; Fernandez *et al*, 2013b, 2020).

GLV peptides are perceived by a family of five LRR‐RKs that function during lateral root initiation and root stem cell niche maintenance. In the latter context, three groups independently found RGF1 INSENSITIVE (RGI) 1‐RGI5 to perceive GLV11 (Ou *et al*, 2016; Shinohara *et al*, 2016; Song *et al*, 2016). GLV11 binds to the ectodomain of RGI1, RGI2, and RGI3 (Shinohara *et al*, 2016; Song *et al*, 2016). Genetically, *RGI1* and *RGI2* are the major genes implicated in root meristematic activity, whereas the role of *RGI3* in this process is less pronounced (Ou *et al*, 2016; Shinohara *et al*, 2016). During GLV‐mediated inhibition of lateral root initiation, RGI1, RGI4, and RGI5 are the main receptors for GLV6 and GLV10 (Fernandez *et al*, 2020). RGI1 can form a GLV11‐induced complex with members of the SOMATIC EMBRYOGENESIS RECEPTOR KINASE (SERK) family (Song *et al*, 2016), which are critical co‐receptor proteins for a multitude of LRR‐type RKs (Ma *et al*, 2016). To relay the extracellular signal into a cellular response, MITOGEN‐ACTIVATED PROTEIN KINASES (MAPKs) become activated after GLV receptor complex activation (Fernandez *et al*, 2020; Lu *et al*, 2020; Shao *et al*, 2020).

In the primary root, GLV11 controls meristematic activity through stimulating the accumulation of the transcription factor PLETHORA (PLT) both transcriptionally and posttranscriptionally (Matsuzaki *et al*, 2010). GLV11 regulates PLT stability through RGI‐dependent transcriptional upregulation of the transcription factor RGF1‐INDUCIBLE TRANSCRIPTION FACTOR 1 (RITF1) and subsequent distribution of reactive oxygen species (ROS) along the developmental zones of roots (Yamada *et al*, 2019). To control root gravitropism, GLV1 and GLV3 increase the abundance and subcellular trafficking of the auxin efflux carrier PINOID 2 (PIN2) largely independent of transcription, indicating that GLV signaling can influence the accumulation and/or stability of diverse proteins and, in the case of plasma membrane proteins, can additionally regulate their endocytosis, vesicle delivery, and/or endosomal sorting (Whitford *et al*, 2012).

A multitude of specific functions of GLV peptides are known for several aspects of root development. A most recent report also indicates a function for the leaf‐expressed GLV4 in plant immunity. *GLV4* gene expression is rapidly induced in response to flg22 treatment and inducible expression of GLV4 peptide precursors induces PTI‐like responses dependent on leaf‐expressed *RGI4* and *RGI5* (Wang *et al*, 2021). These findings suggest an involvement of GLV peptides in the regulation of aboveground immunity. Yet, it remains unclear whether GLV peptides modulate immune signaling by PRRs and if so, what would be the underlying molecular mode of action.

Here, we show that additional shoot‐expressed GLV peptides have a function in modulating immune responses. Overexpression of *GLV1* and *GLV2* peptide precursor genes, as well as simultaneous genetic loss of leaf‐expressed *GLVs*, shows that GLV peptides are positive regulators of PTI and antibacterial resistance. GLV2 is perceived by leaf‐expressed RGI3 which forms a flg22‐ and GLV2‐induced complex with FLS2, suggesting that GLV‐RGI signaling constitutes a peptide receptor pathway controlling PTI. GLV2 perception increases FLS2 and EFR abundance independent of transcriptional regulation, providing a mechanistic basis for GLV‐mediated PTI regulation and a previously undescribed role for GLV peptides in aboveground tissue as PTI‐promoting phytocytokines.

## Results

### *GLVs* are involved in flg22‐triggered PTI

**Figure EV1:**
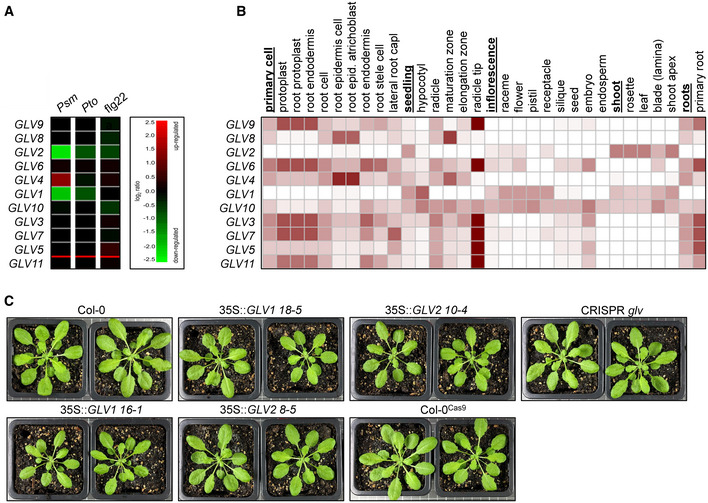
*GLV* peptide differential expression and morphology of *GLV* overexpression lines and CRISPR *glv* Both *GLV1* and *GLV2* are transcriptionally downregulated after infection with *Psm* and to a lesser extent after infection with *Pto*. Treatment with flg22 does not affect *GLV* gene expression. Data were obtained using Genevestigator software and are based on the AT_nRNASeq_ARABI_GL‐1 data set.Tissue‐specific expression pattern of *GLV* family members. *GLV2* is predicted to be the strongest expressed *GLV* member in *Arabidopsis* mature leaves. Data were obtained using Genevestigator software and are based on the AT_mRNASeq_ARABI_GL‐1 data set.Pictures of 6‐week‐old plants of the indicated genotypes.

**Figure 1:**
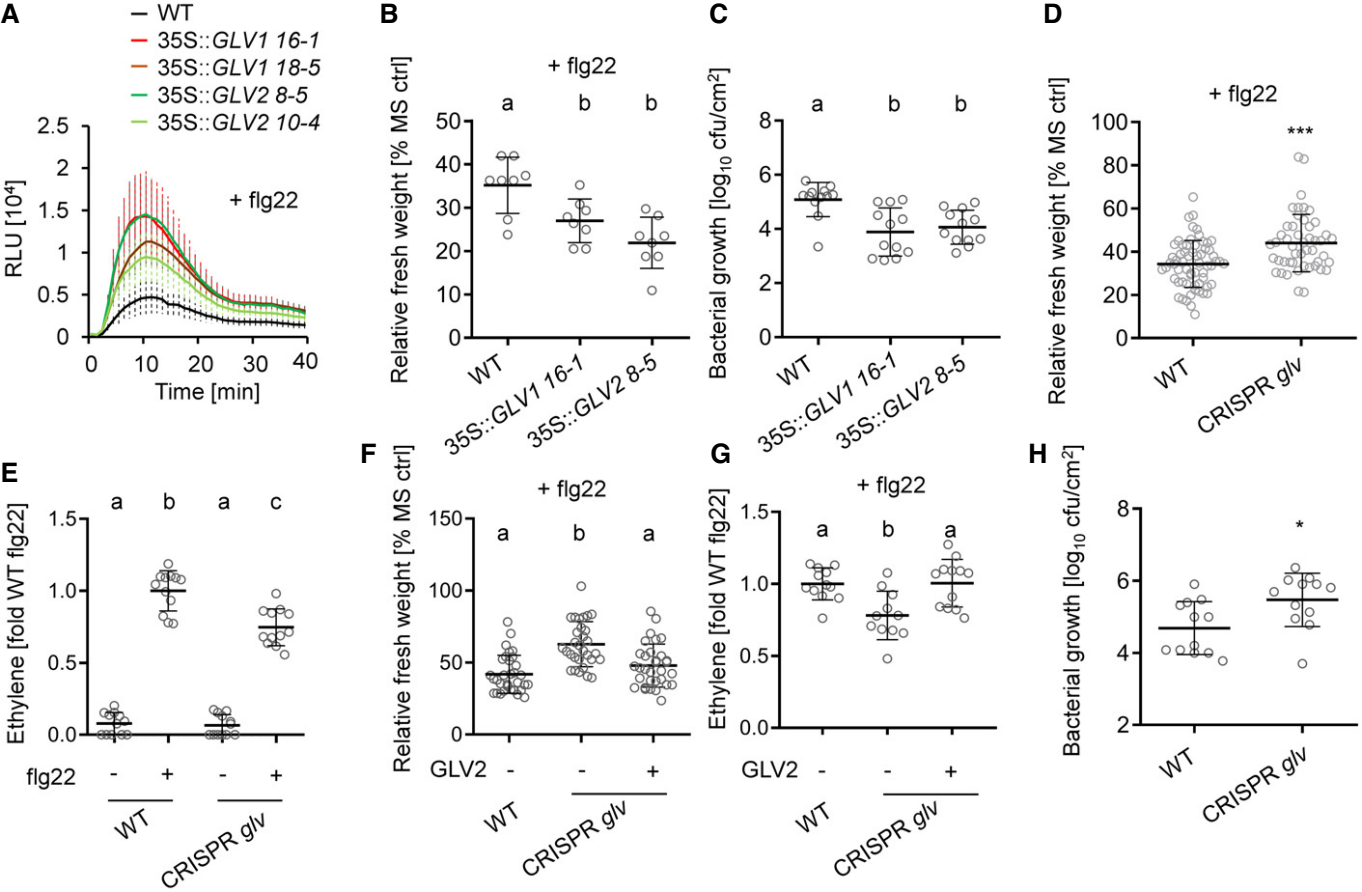
*GLVs* are positive regulators of flg22‐triggered PTI ROS burst in wild type (WT) and *35S::GLV1* and 35S::*GLV2* after elicitation with 100 nM flg22. The kinetic of ROS production is shown over 40 min ± SE, *n* = 8 biological replicates. RLU = Relative light units. Similar results were obtained in three independent experiments.Five‐day‐old seedlings of WT, *35S::GLV1 16‐1*, and *35S::GLV2 8‐5* were treated with 100 nM flg22 for 7 days before measuring fresh weight. Shown is the mean of relative fresh weight compared to the MS medium control ± SD, *n* = 8 biological replicates (one‐way ANOVA, Dunnett *post hoc* test, a‐b *P* < 0.05). Similar results were obtained in three independent experiments.Mean colony forming units (cfu) of *Pto* DC3000 COR‐ 4 days after spray infection of WT, *35S::GLV1 16‐1*, and *35S::GLV2 8‐5*; *n* = 12 biological replicates from three pooled experiments ± SD (one‐way ANOVA, Dunnett *post hoc* test; a‐b, *P* < 0.01).Five‐day‐old seedlings of Col‐0^Cas9^ WT and CRISPR *glv* were treated with 100 nM flg22 for 7 days before measuring fresh weight. Shown is the mean of relative fresh weight compared to the MS medium control ± SD, *n* = 40 biological replicates from five pooled experiments (Student’s *t*‐test, ***P < 0.001).Ethylene production was measured in Col‐0^Cas9^ WT and CRISPR *glv* 3.5 h after elicitation with 500 nM flg22. Shown is the mean of *n* = 12 biological replicates from three pooled experiments ± SD (one‐way ANOVA, Tukey *post hoc* test, a‐b, *P* < 0.001; a‐c, *P* < 0.001; b‐c, *P* < 0.001). Ethylene production was normalized to the average of WT responses to flg22 set as 1.Five‐day‐old Col‐0^Cas9^ WT and CRISPR *glv* seedlings were treated with 100 nM flg22 or co‐treated with 1 μM GLV2 for 7 days before measuring fresh weight. Shown is the mean of relative fresh weight compared to the MS medium control ± SD, *n* = 30–32 biological replicates from two pooled experiments (one‐way ANOVA, Tukey *post hoc* test, a‐b *P* < 0.001).Ethylene production was measured in Col‐0^Cas9^ WT and CRISPR *glv* 3.5 h after elicitation with 500 nM flg22 or co‐treatment with 1 µM GLV2. Ethylene production was normalized to the average of WT responses to flg22 set as 1. Shown is the mean of *n* = 11–12 biological replicates from three pooled experiments ± SD (one‐way ANOVA, Tukey *post hoc* test, a‐b *P* < 0.01).Mean cfu of *Pto* DC3000 COR‐ 4 days after spray inoculation of Col‐0^Cas9^ WT and CRISPR *glv*; *n* = 12 biological replicates from three pooled experiments ± SD (Student’s *t*‐test, *P < 0.05). Source data are available online for this figure.

**Figure EV2:**
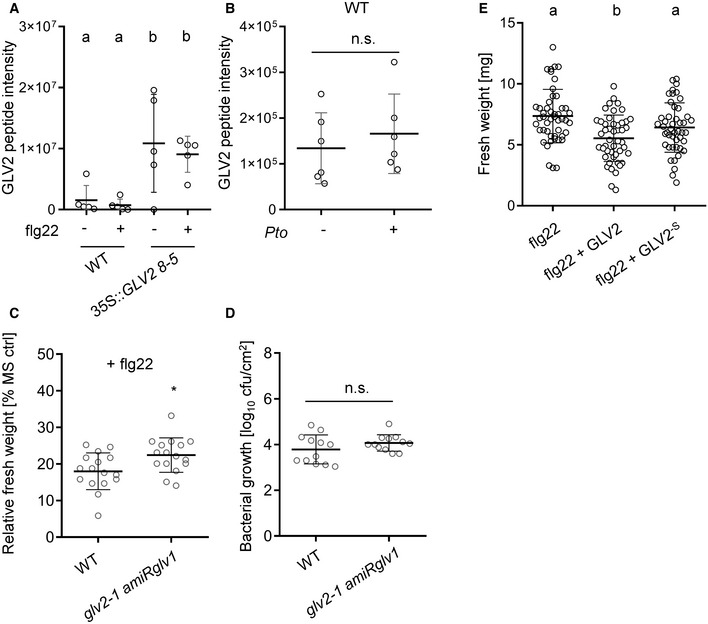
GLV2 peptide detection, phenotypic characterization of *glv1 glv2*, and requirement of GLV2 tyrosine sulfation for its biological activity A, BTargeted proteomics (parallel reaction monitoring, PRM) detection of the GLV2 peptide (DMDY[+80]NSANKKRP[+16]IHN) from apoplastic wash fluids of the indicated genotypes after treatment with 1 h mock or 100 nM flg22 (A) or 24 h postinfection with *Pto* (B). An optimized targeted proteomics assay was setup using a synthetic reference GLV2 peptide (Appendix Table S3). Shown are label‐free mean peptide intensities. The standard deviation is indicated from intensities ± SD, *n* = 5–6 biological replicates (one‐way ANOVA, Dunnett *post hoc* test, a‐b *P* < 0.05).CFive‐day‐old seedlings of the indicated genotypes were treated with 100 nM flg22 for 7 days before measuring fresh weight. Shown is the mean of relative fresh weight compared to the MS control ± SD, *n* = 16 biological replicates (Student’s *t*‐test, *P < 0.05). The *glv2‐1 amiRglv1* lines show reduced flg22‐triggered seedling growth inhibition. Similar results were obtained in three independent experiments.DColony forming units (cfu) of *Pto* DC3000 COR‐ 4 days after spray infection of the indicated genotypes; *n* = 12 biological replicates from three pooled experiments ± SD. Student’s *t*‐test indicates *P* = 0.19. The *glv2‐1 amiRglv1* lines do not show a significantly altered infection phenotype.E5‐day‐old WT seedlings were treated with 10 nM flg22 or co‐treated with 1 μM GLV2 or 1 μM GLV2^‐S^ for 7 days before measuring fresh weight. Shown is the mean of relative fresh weight compared to the MS medium control ± SD, *n* = 45–47 biological replicates from four pooled experiments (one‐way ANOVA, a‐b *P* < 0.001). The GLV2^‐S^ mutant peptide does not significantly enhance flg22‐induced seedling growth inhibition. Source data are available online for this figure.

To identify endogenous peptides involved in immunity, we mined publicly available RNAseq data sets. We focused on members of peptide families that displayed differential transcriptional regulation during biotic stress. With this approach, we found that *GLV1* and *GLV2*, two genes that code for peptides involved in root gravitropism (Whitford *et al*, 2012), are transcriptionally downregulated in leaves after infection with the virulent bacterial strains *Pseudomonas syringae* pv. *maculicola* (*Psm*) and *Pseudomonas syringae* pv. *tomato* (*Pto)* DC3000, albeit with different strengths (Fig EV1A) (Howard *et al*, 2013; Bernsdorff *et al*, 2016). Notably, *GLV1* and *GLV2* did not show altered expression when flg22 was used alone. *GLV1* and *GLV2* are *GLV* members with the strongest predicted expression in leaves (Fig EV1B). Based on these observations, we hypothesized that *GLV1/2* can influence bacterial colonization and perhaps flg22‐triggered responses. To address this, we first used flg22‐induced ROS and seedling growth inhibition (SGI) assays to measure FLS2 activation in *GLV1* and *GLV2* overexpression lines (*35S::GLV1* and *35S::GLV2*) (Whitford *et al*, 2012). Both response outputs were enhanced in *35S::GLV1* and *35S::GLV2* lines (Fig 1A and B). Also, *GLV1* and *GLV2* overexpression lines displayed enhanced resistance against the moderately virulent bacterial pathogen *Pto* DC3000 lacking the effector molecule coronatine (*Pto* DC3000 COR−) (Fig 1C). This demonstrates that overexpression of *GLV1* and *GLV2* elevates flg22‐triggered PTI against bacterial pathogens. *GLV1* and *GLV2* overexpression lines show disturbed root gravitropism and unaltered seedling size (Whitford *et al*, 2012). Grown under our conditions, we observed slightly reduced leaf size compared to the wild type but no overall change in shoot morphology (Fig EV1C). Then, we confirmed that *GLV2* overexpression results in an effective increase in mature GLV2 peptide in the extracellular space of leaves by targeted proteomics on apoplastic wash fluids. We specifically searched for the tyrosine‐sulfated and proline‐hydroxylated GLV2 [DMD(TyrSO_3_H_2_)NSANKKR(Hyp)IHN] that was previously identified in root exudates of *GLV2* overexpression lines (Whitford *et al*, 2012). GLV2 peptide accumulation was strongly increased in *35S::GLV2* plants when compared to wild type (Fig EV2A). We analyzed whether the abundance of mature GLV2 changes upon elicitation with flg22 and only observed a mild but not significant reduction in both the wild type and the overexpression line. Also, 24 h after *Pto* DC3000 infection, we could not detect a significant change in apoplastic GLV2 abundance (Fig EV2B). Thus, we propose that GLV2 secretion is constitutive under the conditions tested and not influenced by flg22‐dependent immune activation or bacterial infection.

Next, we studied the function of GLV peptides in flg22‐triggered PTI by analyzing loss‐of‐function mutants. First, we tested whether flg22‐triggered responses and resistance to *Pto* DC3000 COR‐ are affected in a *glv2‐1 amiRglv1* double‐mutant line (Whitford *et al*, 2012). We noticed a mild reduction in sensitivity toward flg22 in SGI experiments (Fig EV2C), but no increased susceptibility upon *Pto* DC3000 COR‐ infections (Fig EV2D). This suggests that additional GLV peptides may be important for flg22‐triggered PTI in leaf tissue, similar to GLV‐regulated developmental responses in roots (Matsuzaki *et al*, 2010; Whitford *et al*, 2012; Fernandez *et al*, 2020). Consistent with this, the recently identified GLV4 immune‐inducing peptide and four additional GLV members are expressed in shoot and leaf tissue (Fernandez *et al*, 2013a; Wang *et al*, 2021). Thus, we tested a higher‐order CRISPR‐Cas9‐generated *glv* mutant in which the majority of leaf‐expressed *GLVs* (*GLV1*, *GLV2*, *GLV6*, *GLV7*, *GLV8*, and *GLV10*) and the related *CLE18* gene are mutated (Fernandez *et al*, 2020). The resulting CRISPR *glv* mutant was previously described to have increased lateral root density, but no defects in shoot or main root growth (Fernandez *et al*, 2020). Also, grown under our conditions, the CRISPR *glv* mutant did not show changes in adult plant morphology (Fig EV1C). CRISPR *glv* showed reduced flg22‐triggered SGI and ethylene production, respectively (Fig 1D and E). We obtained the precursor‐derived GLV2 peptide by chemical synthesis. Exogenous application of GLV2 could rescue the reduced flg22‐triggered SGI and ethylene phenotype of CRISPR *glv* (Fig 1F and G). The mutant was also significantly more susceptible to *Pto* DC3000 COR‐ (Fig 1H). Together, the data indicate that GLVs are key determinants modulating the intensity of flg22‐triggered PTI.

### GLV2 peptides increase sensitivity of plants to flg22 perception

**Figure 2:**
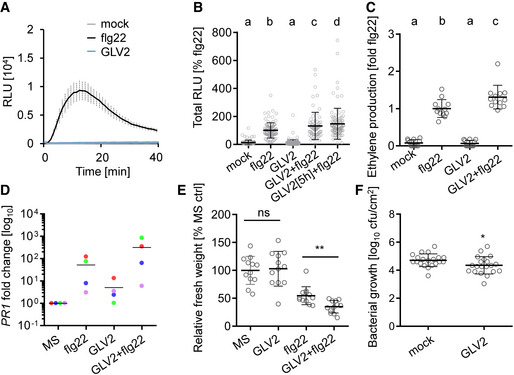
GLV2 treatment enhances flg22‐triggered responses ROS burst in WT after elicitation with 100 nM flg22 or 1 μM GLV2. Shown is the kinetic of ROS production over 40 min ± SEM, *n* = 8 biological replicates. RLU = Relative light units. Similar results were obtained in three independent experiments.ROS burst in WT after elicitation with 10 nM flg22, 1 μM GLV2, co‐treatment or pre‐treatment with 1 μM GLV2 for 5 h prior to elicitation with flg22. Shown is the mean of total ROS production (Total RLU) over 40 min normalized to the flg22 response set as 100% ± SD, *n* = 96–120 biological replicates from 15 pooled experiments (one‐way ANOVA, Tukey *post hoc* test, a‐b/c/d, *P* < 0.001; b‐c, *P* < 0.05; b‐d, *P* < 0.001).Ethylene production was measured in WT after elicitation with 500 nM flg22, 1 μM GLV2, or co‐treatment for 3.5 h. Ethylene production was normalized to the average flg22 response set as 1. Shown is the mean of *n* = 12 biological replicates from three pooled experiments ± SD (one‐way ANOVA, Tukey *post hoc* test, a‐b/c *P* < 0.001; b‐c, *P* < 0.01).
*PR1* expression was measured in 10‐day‐old WT seedlings treated for 24 h with 100 nM flg22, 1 μM GLV2, or co‐treatment. Expression was normalized to the *UBQ* house keeping gene. Shown is the mean of *n* = 4. Symbol colors represent different biological replicatesFive‐day‐old WT seedlings were treated with 10 nM flg22, 1 μM GLV2, or co‐treated for 7 days before measuring fresh weight. Shown is the mean of relative fresh weight compared to the MS medium control ± SD, *n* = 12 biological replicates (Student’s *t*‐test, ns = not significant, **P < 0.01). Similar results were obtained in three independent experiments.Mean cfu of *Pto* DC3000 2 days after syringe inoculation and pre‐treatment with 1 μM GLV2; *n* = 20 biological replicates from five pooled experiments ± SD (Student’s *t*‐test, *P < 0.05). Source data are available online for this figure.

Next, we tested whether the GLV2 peptide can influence flg22‐triggered immunity in wild‐type plants. GLV2 itself did not induce PTI outputs such as an ROS burst response (Fig 2A). However, treating leaf discs with GLV2 together with flg22 increased flg22‐triggered ROS production, a response induced a few minutes after elicitor application (Fig 2B). This effect was even stronger when leaf discs were pre‐treated for 5 h prior to flg22 addition (Fig 2B), suggesting that GLV2 perception results in increased sensitivity of plants to subsequent immune stimulation by flg22. Consistent with that, longer‐term co‐treatment promoted flg22‐triggered signaling outputs. Co‐treatment between flg22 and GLV2 increased flg22‐triggered ethylene production, *PR1* expression, and SGI (Fig 2C–E). Finally, GLV2 treatment induced resistance to subsequent *Pto* DC3000 infection (Fig 2F). These findings reinforce the notion that exogenous application of GLV2 shows a similar effect on flg22‐triggered PTI as endogenous overexpression of the peptide precursor gene.

GLV2 requires sulfation at a conserved tyrosine residue by TPST to control meristematic activity and root gravitropism (Matsuzaki *et al*, 2010; Stührwohldt *et al*, 2020). The *tpst* mutants show increased immune responses which was mainly linked to PSK‐ and PSY1‐mediated immune regulation, both of which are also substrates of TPST (Igarashi *et al*, 2012; Mosher *et al*, 2013). Based on these previous findings, we hypothesized that TPST‐catalyzed GLV tyrosine sulfation may not be required for PTI. Surprisingly though, a GLV2 peptide without sulfated tyrosine (GLV2^‐S^) was ineffective and did not support flg22‐triggered SGI (Fig EV2E), suggesting that tyrosine sulfation is also critical for GLV2’s function during FLS2‐triggered immunity.

### RGI receptor kinases are required for PRR‐mediated signaling

**Figure 3:**
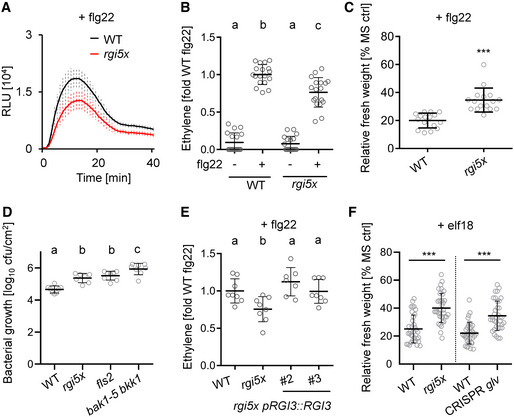
RGI receptors are involved in PTI ROS burst in WT and *rgi5x* after elicitation with 100 nM flg22. Shown is the kinetic of ROS production over 40 min ± SE, *n* = 8 biological replicates. RLU = Relative light units. Similar results were obtained in three independent experiments.Ethylene production was measured in the indicated genotypes 3.5 h after elicitation with mock or 500 nM flg22. Ethylene accumulation was normalized to the average of WT responses to flg22 set as 1. Shown is the mean of *n* = 18 biological replicates from three pooled experiments ± SD (one‐way ANOVA, Tukey *post hoc* test, a‐b *P* < 0.001; a‐c, *P* < 0.001; b‐c, *P* < 0.001).Five‐day‐old seedlings of WT and *rgi5x* were treated with 100 nM flg22 for 7 days before measuring fresh weight. Shown is the mean of relative fresh weight compared to the MS medium control ± SD, *n* = 16 biological replicates (Student’s *t*‐test, ***P < 0.001). Similar results were obtained in three independent experiments.Mean colony forming units (cfu) of *Pto* DC3000 COR‐ 3 days after spray infection of the indicated genotypes; *n* = 7–8 biological replicates from two pooled experiments ± SD (one‐way ANOVA, Tukey *post hoc* test, a‐b *P* < 0.001; a‐c *P* < 0.001, b‐c *P* < 0.05).Ethylene production was measured in WT and *rgi5x* and *rgi5x pRGI3::RGI3 #2* and *#3* 3.5 h after elicitation with 500 nM flg22. Ethylene production was normalized to the average of WT responses to flg22 set as 1. Shown is the mean of *n* = 8 biological replicates from two pooled experiments ± SD (one‐way ANOVA, Dunnett *post hoc* test, a‐b *P* < 0.05).Five‐day‐old seedlings of the indicated genotypes were treated with 100 nM elf18 for 7 days before measuring fresh weight. Shown is the mean of relative fresh weight compared to the MS medium control ± SD, *n* = 36 biological replicates from three pooled experiments (Student’s *t*‐test, ***P < 0.001). Source data are available online for this figure.

**Figure EV3:**
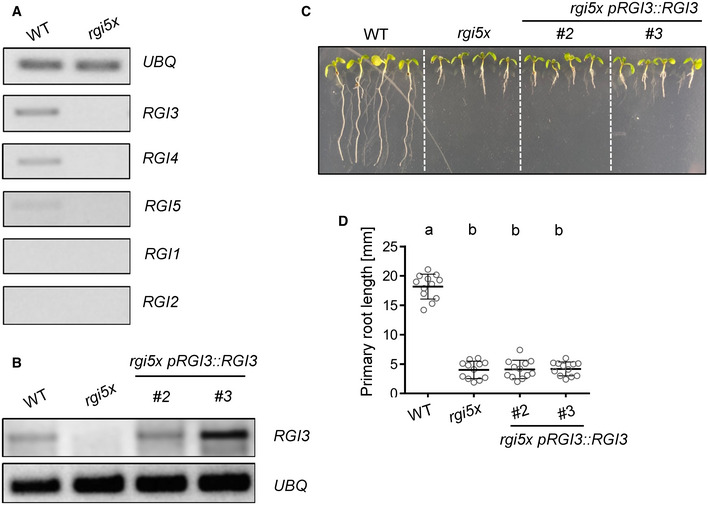
Characterization of *RGI* expression in mature leaves, *RGI3* expression in and phenotypic characterization of *rgi5x pRGI3*::*RGI3* Semi‐quantitative reverse transcription PCR showing expression of *RGI1‐RGI5* in 6‐week‐old leaves of WT and *rgi5x*. *UBQ* was used as a control. *RGI3* and *RGI4* show comparable expression levels. *RGI5* shows weaker transcript levels and *RGI1* and *RGI2* expression was not detectable in mature leaves.Semi‐quantitative reverse transcription PCR showing expression of *RGI3* in transgenic *rgi5x pRGI3::RGI3* lines. *UBQ* was used as a control. Both lines of *rgi5x pRGI3*::*RGI3* show *RGI3* expression in mature leaves.Representative pictures taken 7 days after vertical germination of the indicated genotypes on ½ MS Agar plates.Quantification of main root length of seedlings shown in C. Shown is the mean of *n* = 12 biological replicates ± SD (one‐way ANOVA, Tukey *post hoc* test, a‐b *P* < 0.001). Expression of *pRGI3::RGI3* does not complement the short root phenotype of the *rgi5x* mutant. Data information: Similar results were obtained in three independent experiments. Source data are available online for this figure.

RGI1‐RGI5 are known receptors for GLV peptides (Ou *et al*, 2016; Shinohara *et al*, 2016; Song *et al*, 2016; Fernandez *et al*, 2020). We tested whether RGI1‐RGI5 may be involved in flg22‐triggered responses. The *rgi1/2/3/4/5* quintuple mutant (hereafter referred to as *rgi5x*) showed reduced flg22‐triggered ROS production and ethylene accumulation, respectively (Fig 3A and B). Because both assays are performed with leaf discs, these findings suggest that RGIs have additional functions in leaves where they are involved in flg22‐triggered signaling. The *rgi5x* mutant also showed reduced sensitivity to flg22 in SGI experiments (Fig 3C), confirming a positive regulatory role of RGIs for flg22‐induced responses. Consistent with the recently proposed role for leaf‐expressed RGIs in antibacterial resistance, the *rgi5x* mutant was more susceptible to *Pto* DC3000 COR‐ (Wang *et al*, 2021). The enhanced susceptibility of *rgi5x* was similar to *fls2* loss‐of‐function mutants but less pronounced compared to *bak1‐5 bkk1*, a mutant strongly affected in PTI upon perception of multiple MAMPs (Fig 3D) (Roux *et al*, 2011). Importantly, the *rgi5x* mutant shows severely reduced root growth but no morphological phenotypes in aboveground tissue (Ou *et al*, 2016). *RGI3* is one of three *RGIs* (*RGI3*, *RGI4*, *and RGI5*) expressed in leaves (Klepikova *et al*, 2016). We confirmed that *RGI3* is expressed in leaves from 6‐week‐old plants by semi‐quantitative reverse transcription PCR (Fig EV3A). *RGI3* transcript accumulation was similar to *RGI4* but stronger than *RGI5*. This is in contrast to previous findings which analyzed *RGI3* expression in shoots from seedlings (Wang *et al*, 2021). Thus, we attempted to complement the PTI phenotypes of *rgi5x* with *RGI3* driven under its native promoter (*pRGI3::RGI3*). Expression of *RGI3* complemented the reduced flg22‐induced ethylene production phenotype of the *rgi5x* mutant (Figs 3E and EV3B). Interestingly, the short root phenotype of *rgi5x* was not complemented by *pRGI3::RGI3* (Fig EV3C and D). Based on this, we propose that RGIs are selectively required in distinct processes such as root growth regulation and immunomodulation in leaves. We also tested whether GLV‐RGI‐mediated regulation of PTI extents to other MAMP perception pathways. We found that *rgi5x* and CRISPR *glv* mutants showed impaired seedling growth inhibition in response to elf18 (Fig 3F). This suggests that the GLV‐RGI module is important for the regulation of different PRR signaling pathways and not exclusive for FLS2‐triggered immunity.

### RGI3 perceives GLV2 to regulate PTI

**Figure 4:**
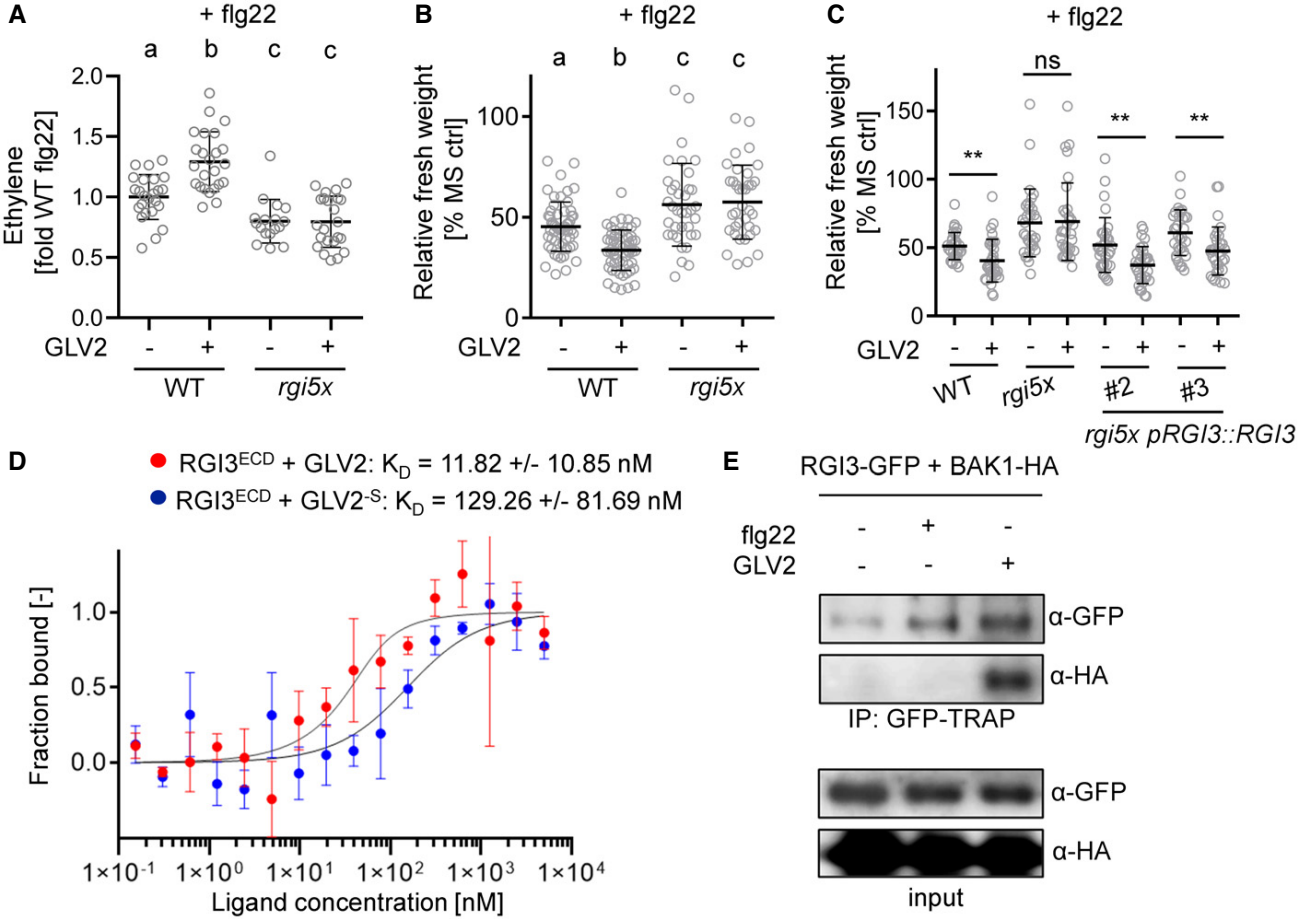
RGI3 is required for GLV2 perception during PTI Ethylene production was measured in WT and *rgi5x* 3.5 h after elicitation with 500 nM flg22 or co‐treatment with 1 μM GLV2. Shown is the mean of *n* = 17–24 biological replicates from four pooled experiments ± SD (one‐way ANOVA, Tukey *post hoc* test, a‐b *P* < 0.001; a‐c, *P* < 0.05). Ethylene production was normalized to the average of WT responses to flg22 set as 1.Five‐day‐old seedlings of WT and *rgi5x* were treated with 10 nM flg22 or co‐treated with 1 μM GLV2 for 7 days before measuring fresh weight. Shown is the mean of relative fresh weight compared to the MS medium control ± SD, *n* = 36–57 biological replicates pooled from four experiments (one‐way ANOVA, Tukey *post hoc* test, a‐b *P* < 0.001; a‐c, *P* < 0.01).Five‐day‐old seedlings of WT, *rgi5x*, and *rgi5x pRGI3*::*RGI3 #2* and *#3* were treated with 10 nM flg22 or co‐treated with 1 μM GLV2 for 7 days before measuring fresh weight. Shown is the mean of relative fresh weight compared to the MS medium control ± SD, *n* = 32 biological replicates pooled from four experiments (Student’s *t*‐test, **P < 0.01).Quantitative analysis of synthetic GLV2 or GLV2^‐S^ peptide binding to RGI3^ECD^ using microscale thermophoresis. Shown is the mean ± SD, *n* = 3 technical replicates.Co‐immunoprecipitation experiment upon co‐expression of BAK1‐HA and RGI3‐GFP in *N. benthamiana*. IP was performed using GFP‐TRAP agarose beads. Western blots for protein detection were probed with α‐GFP or α‐HA antibodies. Similar results were obtained in three independent experiments. Source data are available online for this figure.

Next, we tested whether RGIs are required for GLV2 function during PTI. We found that the GLV2‐mediated increase in flg22‐induced ethylene production and SGI was abolished in *rgi5x* mutants (Fig 4A and B). Therefore, RGIs are genetically required for the GLV2‐triggered modulation of immune responses. Since expression of *RGI3* alone is sufficient to rescue the GLV2‐mediated increase in flg22‐induced SGI in *rgi5x* mutants (Fig 4C), we predicted that RGI3 could act as a GLV2 receptor. To test if GLV2 can interact with RGI3, we purified the ectodomain of RGI3 (RGI3^ECD^) to near homogeneity and used microscale thermophoresis (MST). These experiments revealed a strong interaction between RGI3^ECD^ and GLV2 with a dissociation constant (*K*_D_) of 11.82 (± 10.85 nM, Fig 4D). The interaction was reduced when GLV2^‐S^ was used instead of the WT peptide (*K*_D_ = 129.26 ± 81.69 nM). This is in line with previous reports on sulfated peptide–LRR‐RK quantitative interactions determined by MST (Luu *et al*, 2019). Thus, RGI3 is a *bona fide* and high‐affinity receptor for GLV2. RGI receptors were previously shown to form ligand‐induced complexes with BRASSINOSTEROID INSENSITIVE 1‐ASSOCIATED RECEPTOR KINASE 1 (BAK1) and related SERK family proteins (Song *et al*, 2016; Wang *et al*, 2021). We co‐expressed RGI3‐GFP and BAK1‐HA in *N. benthamiana* experiments. GLV2, but not flg22, induced an interaction between RGI3‐GFP and BAK1‐HA (Fig 4E). This is in accordance with previous reports showing that GLV peptides can induce RGI‐BAK1/SERK complexes (Song *et al*, 2016; Wang *et al*, 2021) and further corroborates our findings that RGI3 is a receptor for GLV2.

### RGI3 forms a flg22‐ and GLV2‐induced complex with FLS2

**Figure 5:**
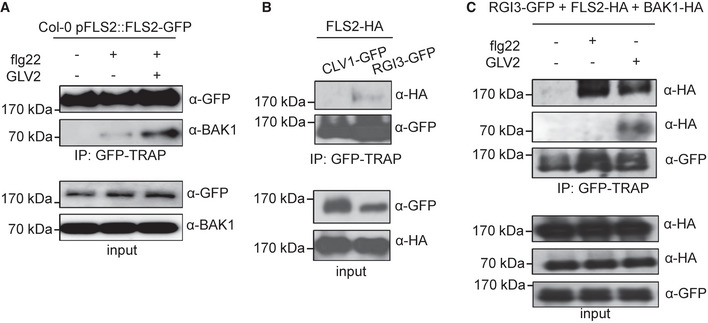
RGI3 forms a flg22‐ and GLV2‐induced complex with FLS2 Co‐immunoprecipitation experiment in 12‐day‐old Col‐0 pFLS2::FLS2‐GFP seedlings. Protein extraction was performed 10 min after mock, 1 μM flg22, or 1 μM flg22 + 1 μM GLV2 treatment. IP was performed using GFP‐TRAP agarose beads. Western blots for protein detection were probed with α‐GFP or α‐BAK1 antibodies.Co‐immunoprecipitation experiment upon co‐expression of FLS2‐HA with either CLV1‐GFP or RGI3‐GFP in *N. benthamiana*. IP was performed using GFP‐TRAP agarose beads. Western blots for protein detection were probed with α‐GFP or α‐HA antibodies.Co‐immunoprecipitation experiment upon co‐expression of FLS2‐HA, BAK1‐HA, and RGI3‐GFP in *N. benthamiana*. Protein extraction was performed 30 min after mock, 1 μM flg22, or 1 μM GLV2 treatment. IP was performed using GFP‐TRAP agarose beads. Western blots for protein detection were probed with α‐GFP or α‐HA antibodies. Data information: Similar results were obtained in three independent experiments. Source data are available online for this figure.

Phytocytokines can regulate immune responses mediated by PRRs, but the mechanistic details of their mode‐of‐action remains largely unknown (Segonzac & Monaghan, 2019). Here, we found that GLV2 peptides can promote FLS2‐triggered immunity. Thus, we aimed at understanding the potential mechanism by which the GLV2‐RGI3 module controls FLS2 function. GLV2 co‐treatment enhanced flg22‐triggered responses, an effect that was more pronounced upon pre‐treatment or longer‐term co‐treatment (Fig 2). We tested whether GLV2 perception has an influence on FLS2‐BAK1 complex formation which is the first measurable output in response to flg22 treatment (Chinchilla *et al*, 2007; Heese *et al*, 2007). Indeed, GLV2 co‐treatment promoted FLS2‐BAK1 complex formation, suggesting that GLV2‐RGI3 may be part of PRR complexes (Fig 5A). Hence, we tested whether RGI3 may interact with FLS2. We expressed RGI3‐GFP in *Nicotiana benthamiana* leaves and performed co‐immunoprecipitation experiments using the LRR‐RK CLAVATA1 (CLV1) as a negative control. FLS2‐HA associated with RGI3‐GFP but not CLV1‐GFP (Fig 5B). The interaction between FLS2‐HA and RGI3‐GFP was weak. Thus, we tested whether treatment with flg22 or GLV2 may influence this association. Interestingly, both flg22 and GLV2 treatment strongly enhanced the interaction between RGI3‐GFP and FLS2‐HA (Fig 5C). We simultaneously expressed BAK1‐HA. GLV2, but not flg22, induced an interaction between RGI3‐GFP and BAK1‐HA (Fig 5C). Collectively, our data suggest that RGI3 forms ligand‐induced complexes with FLS2 which may underlie its immune‐regulatory function.

### GLV2 perception by RGIs regulates posttranscriptional PRR abundance

**Figure 6:**
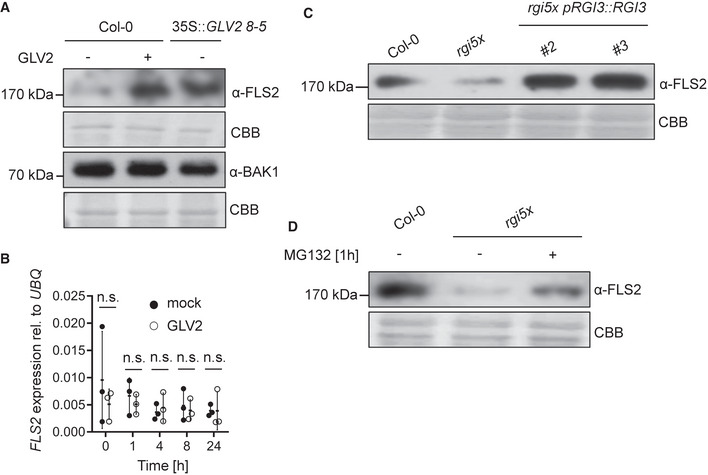
GLV2 perception by RGIs promotes maximal FLS2 abundance independent of transcription Twelve‐day‐old WT or *35S::GLV2 8‐5* seedlings were treated with mock or 1 μM GLV2 for 24 h before protein extraction. Western blots were probed with α‐FLS2 or α‐BAK1 antibodies. CBB: Coomassie brilliant blue.Quantitative real‐time PCR of *FLS2* transcripts from 12‐day‐old seedlings treated with mock or 1 μM GLV2 for the indicated time, *n* = 3 biological replicates. *UBQ* was used as a house keeping gene. Student’s *t*‐test revealed no statistical difference for each of the different time points.Protein extraction was performed from 12‐day‐old seedlings of the indicated genotypes. Western blot was probed with α‐FLS2 antibodies.Protein extraction was performed from 12‐day‐old seedlings after 1 h treatment with mock or 50 μM MG132. Western blots were probed with α‐FLS2 antibodies. Data information: Similar results were obtained in three independent experiments. Source data are available online for this figure.

**Figure EV4:**
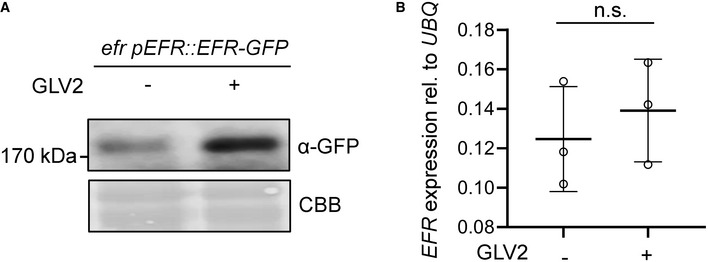
GLV2 treatment promotes EFR abundance Twelve‐day‐old *efr* pEFR::EFR‐GFP seedlings were treated with mock or 1 μM GLV2 for 24 h before protein extraction. Western blots were probed with α‐GFP antibodies. CBB: Coomassie brilliant blue.Quantitative real‐time PCR of *EFR* transcripts from 12‐day‐old *efr* pEFR::EFR‐GFP seedlings treated with mock or 1 μM GLV2 for 24 h, *n* = 3 biological replicates ± SD. *UBQ* was used as a house keeping gene. Student’s *t*‐test revealed no statistical difference. Data information: Similar results were obtained in three independent experiments. Source data are available online for this figure.

GLV signaling was shown to control the stability of the PLT transcription factor and the auxin efflux carrier PIN2 during root stem cell niche maintenance and root gravitropism, respectively (Matsuzaki *et al*, 2010; Whitford *et al*, 2012). We tested whether RGIs may similarly control the accumulation of FLS2. Indeed, we found that both treatment of seedlings with GLV2 for 24 h or *GLV2* precursor overexpression resulted in increased FLS2 protein levels (Fig 6A). In contrast, the abundance of the FLS2 co‐receptor BAK1 was not altered by GLV2 treatment or *GLV2* overexpression. Moreover, GLV2 treatment promoted abundance of EFR in an *efr* pEFR‐EFR‐GFP line (Fig EV4A). Because neither *FLS2* nor *EFR* transcript accumulation was affected by GLV2 treatment (Figs 6B and EV4B), we propose that GLV2 signaling modulates FLS2 and EFR accumulation at the posttranscriptional level. This is reminiscent of the role of GLV1 and GLV3 in increasing the abundance of PIN2 during root gravitropism (Whitford *et al*, 2012). Consistent with this newly discovered function for the GLV2 peptide, the *rgi5x* mutant showed impaired FLS2 accumulation which was complemented by expression of *pRGI3::RGI3* (Fig 6C). The FLS2 accumulation defect of *rgi5x* could be partially rescued by treatment with the 26S proteasome inhibitor MG132 (Fig 6D), suggesting that RGIs control ubiquitination‐dependent FLS2 stability. Collectively, our results suggest that FLS2 and EFR abundance, as well as FLS2‐BAK1 complex formation (Fig 5A), is regulated by GLV2‐RGI signaling. These findings reveal mechanistic insight into GLV phytocytokine function, underpinning its role in the control of plant immunity.

## Discussion

Tight regulation of the on and off set of immune responses is critical to ensure the plants survival and to prevent excessive defense responses in the absence or ceasing of infection. The trade‐off between growth and immunity can be orchestrated by the complex regulation of RK signaling through endogenous plant peptide ligands, having a dual function as growth modulators and phytocytokines (Gust *et al*, 2017).

GLV peptides have regulatory functions controlling root meristem activity, gravitropism, and lateral root emergence but a function for this family of peptides in shoot or leaf tissue remained unknown until recently (Wang *et al*, 2021). We identified the mature GLV2 peptide in apoplastic wash fluids from leaves in wild‐type plants (Fig EV2A), demonstrating that GLV2 is also expressed, processed, and secreted in shoots comparable to roots (Whitford *et al*, 2012). Several lines of evidence show that leaf‐expressed and ‐secreted GLV peptides control immunity by regulating PRR levels. While the overexpression of *GLV1* and *GLV2* increases flg22‐triggered responses and resistance to *Pto*, the genetic elimination of leaf‐expressed GLVs results in impaired flg22 and elf18 signaling, as well as increased susceptibility to *Pto* (Figs 1 and 3F). PTI‐promoting effects of synthetic GLV2 peptides further support that the mature apoplastic peptide in GLV overexpression lines is likely responsible for their immune phenotype (Fig 2). We show that RGIs are positive regulators of flg22‐ and elf18‐induced responses and resistance to *Pto* and that RGI3 is a GLV2 receptor in leaf tissue to mediate its function during PTI (Figs 3 and 4). GLV2 promotes flg22‐induced FLS2‐BAK1 complex formation and FLS2 and EFR protein abundance independent of transcription (Figs 6 and EV4) and its receptor RGI3 forms a flg22‐ and GLV2‐induced complex with FLS2 (Fig 5).

PIP, PEP, and SCOOP signaling through RLK7, PEP RECEPTOR 1/2, and MIK2 induce PTI‐like response such as ROS production or upregulation of defense‐related genes (Flury *et al*, 2013; Tintor *et al*, 2013; Hou *et al*, 2014; Gully *et al*, 2019; Rhodes *et al*, 2021). Unlike these previously described phytocytokines, GLV2 does not induce on its own PTI‐like responses but rather acts to modulate the amplitude of FLS2 immune signaling (Fig 2). Consistent with our findings, the inducible overexpression of GLV4 was shown to activate MAPKs and expression of PTI‐related genes (Wang *et al*, 2021). Yet, exogenous GLV4 peptide application does not elicit PTI, indicating that GLV4, as we demonstrate for GLV2 here, is rather an immune responses modulator. PIP1 and PEP perception leads to the upregulation of *FLS2* transcription to cooperatively contribute to PTI against bacterial infection (Flury *et al*, 2013; Tintor *et al*, 2013; Hou *et al*, 2014). In contrast, we discovered that GLV2 signaling through RGIs posttranscriptionally increases FLS2 and EFR abundance. Thus, our study reveals a previously unknown regulatory mechanism for phytocytokine functions independent of *PRR* gene expression, which controls optimal FLS2 and EFR protein levels for immune activation (Figs 6 and EV4).

In addition to GLVs, other growth regulatory peptides act as phytocytokines, suggesting that endogenous peptide signaling pathways linking growth to PTI may be more widespread than anticipated. Yet, the immunomodulatory molecular mechanisms remain to be discovered. The PSK and PSY1 receptors PSKR1/2 and PSY1R, respectively, negatively regulate flg22‐induced immune responses (Igarashi *et al*, 2012; Mosher *et al*, 2013), but it is unknown whether these are also part of PRR signaling platforms or whether their signal transduction pathways intersect indirectly downstream of receptor activation. FLS2 signaling is controlled by endogenous RALF peptides via their receptor FER, which constitutively interacts with FLS2 and functions as a ligand‐regulated scaffold for FLS2‐BAK1 complexes (Stegmann *et al*, 2017). Our data now indicate that RGI3 is recruited to an flg22‐activated FLS2 complex (Fig 5) providing the first line of evidence for the direct regulation of PRR signaling by a growth‐related and peptide‐binding RK as part of induced PRR signaling platforms. GLV2 also induces RGI3‐FLS2 interaction which suggests the formation of dynamic RGI3‐FLS2 complexes in the presence of exogenous and endogenous ligands. GLV‐RGI signaling also controls elf18‐induced responses (Fig 3F), suggesting that RGIs may associate with additional LRR‐type PRRs. Whether GLV‐RGI signaling also controls signaling by PRRs with other types of extracellular domains remains to be tested. RGI3 did not co‐immunoprecipitate BAK1 after flg22 treatment which raises the question whether RGI3 may preferentially associate with a BAK1‐free pool of FLS2. However, this may also be explained by detection limitations as BAK1 would only indirectly associate with RGI3 via FLS2 in this experimental setup. Future studies need to determine if RGI3 forms tripartite complexes with PRRs and BAK1 in an activation status‐dependent manner.

GLV signaling was previously shown to increase the levels of specific growth‐related proteins (Matsuzaki *et al*, 2010; Whitford *et al*, 2012). GLV1 and GLV3 treatment increased PIN2 stability and subcellular dynamics by elevating both intracellular PIN2‐containing vesicles and PIN2 localization to the plasma membrane (Whitford *et al*, 2012). Similar to PIN2, FLS2 and EFR are subject to vesicular trafficking (Robatzek *et al*, 2006; Beck *et al*, 2012; Mbengue *et al*, 2016) raising the possibility that GLV2 acts via RGI3 to regulate PRR endomembrane dynamics. GLV2 is secreted independent of immune activation (Fig EV2A and B) and induces FLS2‐RGI3 association (Fig 5C). Therefore, GLV2‐RGI3 signaling may regulate steady‐state FLS2 and EFR abundance by directly or indirectly controlling posttranslational modifications underlying FLS2 stability or endosomal trafficking. Ligand‐bound FLS2 and EFR undergo clathrin‐dependent endocytosis through distinct subcellular compartments on their way to the vacuole for degradation (Beck *et al*, 2012; Mbengue *et al*, 2016). FLS2 endocytosis is potentially regulated by ubiquitination through plant U‐box‐type E3 ubiquitin ligases (PUBs). PUB12/13 associate with the activated FLS2 complex and ubiquitinate FLS2 for subsequent degradation (Lu *et al*, 2011). Similarly, RGI3 is recruited to the FLS2 complex in a flg22‐dependent manner (Fig 5) and the defects of FLS2 accumulation in *rgi5x* could be partially complemented by proteasome inhibitor treatment (Fig 6D). This raises the question whether RGI3 may interfere with PUB12/13‐mediated FLS2 or EFR degradation. Yet, *pub12/13* mutants do not show defects in steady‐state FLS2 abundance (Lu *et al*, 2011) and thus GLV2‐RGI3 signaling may require additional components to facilitate PRR abundance control. Unlike FLS2, EFR accumulation is dependent on functional endoplasmic reticulum quality control (Li *et al*, 2009; Nekrasov *et al*, 2009; Saijo *et al*, 2009) and we cannot exclude that GLV2‐RGI3 may also modulate this regulatory pathway. However, this seems less likely as EFR and FLS2 are regulated by GLV2‐RGI3 in a similar way.

Our data also reveal that short‐term GLV2 treatments can promote flg22‐induced FLS2‐BAK1 complex formation (Fig 5A). This suggests that RGI‐GLV2 modulates both PRR accumulation and formation of signaling competent PRR platforms. FER controls FLS2‐BAK1 and EFR‐BAK1 complex formation independent of protein accumulation (Stegmann *et al*, 2017). In contrast to GLV2 treatment, the FER ligand RALF23 inhibits PRR‐BAK1 complex formation. Our data now reveals a phytocytokine enhancing flg22‐induced FLS2‐BAK1 complex assembly. Whether GLV2’s promotion of PRR complex formation and the regulation of PRR accumulation are mechanistically coupled remains to be tested.

*GLV1*/*2* gene expression is not affected by flg22 perception (Fig EV1A). By contrast, auxin induces *GLV1* and *GLV2* expression (Whitford *et al*, 2012), raising the possibility that GLV signaling coordinates hormone responses with cell surface immunity. This may represent a mechanism to fine‐tune the accumulation of PRRs for the detection of microbial intruders under fluctuating environmental conditions. Interestingly, *GLV4* is transcriptionally upregulated after flg22 perception (Wang *et al*, 2021). It remains to be tested whether increased *GLV4* expression translates into more abundant peptides in the apoplast and whether GLV4‐RGI4/5 similarly controls PRR levels or may constitute an independent pathway for danger perception.

Before GLVs are secreted and exert their physiological role, they require maturation through consecutive cleavage events by subtilases (SBTs) on the peptide precursors’ passage through the secretory pathway. Pre‐processing of GLV2 is executed by SBT6.1/S1P (Ghorbani *et al*, 2016; Stührwohldt *et al*, 2020). Intriguingly, S1P is additionally maturating RALF23 peptides which attenuate immunity by inhibiting the PRR scaffolding function of FER (Srivastava *et al*, 2008; Stegmann *et al*, 2017). Production of mature GLV peptides also requires TPST‐dependent tyrosine sulfation (Whitford *et al*, 2012; Song *et al*, 2016; Kaufmann & Sauter, 2019). Accordingly, GLV2^‐S^ has a lower affinity to RGI3 and is not capable of enhancing flg22‐induced immune signaling (Figs 4D and EV2E). TPST was previously shown to negatively regulate immunity against *Pto* via PSK and PSY1 peptides (Igarashi *et al*, 2012; Mosher *et al*, 2013). Our work now reveals that tyrosine‐sulfated peptides can also stimulate immune responses. This highlights the complexity and interconnectivity of diverse RK–peptide pathways in the plants’ adaptation to stress responses. It is remarkable how many different signaling peptides modulate immune responses and that some have opposing functions although they partially share the same enzymes for maturation. In summary, future studies need to address the complex physiological interplay of phytocytokines, including PSK, PSY1, RALF, and GLV peptides, addressing the spatio‐temporal expression, maturation, and/or secretion dynamics of these functionally distinct peptides during immune responses.

## Materials and Methods

### Plant material and growth conditions

*Arabidopsis* ecotype Columbia (Col‐0) was used as a wild‐type control for all plant assays. The CRISPR *glv* mutant plants were compared to Col‐0‐expressing Cas9 (Fernandez *et al*, 2020). The 35S::*GLV1 16‐1/18‐5* and 35S::*GLV2 8‐5/10‐4* were kindly provided by Ana Fernandez (Whitford *et al*, 2012). The *rgi1‐1/rgi2‐1/rgi3‐1/rgi4‐1/rgi5/1* (*rgi5x*) mutant was kindly provided by Jia Li (Ou *et al*, 2016). The Col‐0 pFLS2::FLS2‐GFP and *efr* pEFR::EFR‐GFP lines were described earlier (Göhre *et al*, 2008; Nekrasov *et al*, 2009). Plants for ROS burst assays, ethylene measurements, and *Pto* infection experiments were grown in individual pots at 20–21°C with an 8 h photoperiod in environmentally controlled growth rooms. For seedling‐based assays, seeds were sterilized using chlorine gas for 4 h and grown on ½ Murashige and Skoog (MS) media supplemented with vitamins, 1% sucrose, and 0.8% agar at 22°C and a 16 h photoperiod. For transient co‐immunoprecipitation experiments, *Nicotiana benthamiana* plants were grown for 4 weeks at 22°C and a 16 h photoperiod.

### Molecular cloning

To generate *pRGI3*‐driven *RGI3* complementation lines in the *rgi5x* background, *pRGI3::RGI3* was cloned from genomic DNA containing 1,404 bp upstream of the start codon with primers pRGI3_F and pRGI3_R containing attB attachment sites for subsequent BP reactions. The PCR product was cloned into pDONR223 (Thermo Fisher, Germering, Germany) using BP clonase (Thermo Fisher). The resulting pDONR223 pRGI3::RGI3 construct was subcloned into pGWB4 (Nakagawa *et al*, 2007) using LR clonase (Thermo Fisher). The resulting construct was sequenced and transformed into *Agrobacterium tumefaciens* strain GV3101. Subsequently, *rgi5x* mutants were transformed via floral dip. To generate the *35S*::*RGI3*‐*GFP* construct for transient expression experiments, *RGI3* was PCR amplified from Col‐0 cDNA using primers RGI3_F and RGI3_R. The PCR product was cloned into pDONR223 (Thermo Fisher) and subsequently recombined with pK7FWG2 (VIB Ghent) using LR clonase (Thermo Fisher).

To generate fusion constructs for transient overexpression of CLV1‐GFP, FLS2‐HA, and BAK1‐HA, full‐length coding sequence of *CLV1* was PCR amplified from Col‐0 cDNA using primers CLV1_F and CLV1_R and cloned into a GoldenGate‐adapted pUC18‐based vector similar to previously described (Engler *et al*, 2008). *CLV1* was further domesticated to remove an internal AarI restriction site via whole‐plasmid PCR amplification using primers CLV1‐AarI_F and CLV1‐AarI_R and GoldenGate‐based plasmid recirculation. For cloning *FLS2*, two fragments were amplified from Col‐0 cDNA to remove an internal EciI restriction site with primers FLS2_1_F, FLS2_1_R (FLS2 fragment 1) and FLS2_2_F, FLS2_2_R (FLS2 fragment 2). An internal *FLS2* AarI restriction site was removed as described above using primers FLS2‐AarI_F and FLS2‐AarI_R. Full‐length *BAK1* was amplified from Col‐0 cDNA using primers BAK1_F and BAK1_R. *CLV1*, *FLS2*, and *BAK1* coding sequences were subsequently fused to C‐terminal meGFP or 1xHA epitope tags with an eleven‐ or six‐glycine linker, respectively, and CaMV35S promoter and terminator sequences. Finally, full expression cassettes were cloned into a GoldenGate‐adapted binary pCB302 vector. Sequences of all primers can be found in Appendix Table S2.

### ROS burst measurements

For ROS burst measurements, leaf discs (3 mm diameter) of *Arabidopsis* were collected in 96‐well plates using biopsy punchers and floated overnight on 100 μl H_2_O in a 96‐well plate. Elicitation was performed with the indicated concentration of peptides and 2 μg/ml horseradish peroxidase (Type II, Roche, Penzberg, Germany) and 5 μM L‐012 (FUJIFILM Wako chemicals, Neuss, Germany). Luminescence was measured as relative light units (RLU) in 1min intervals using a Tecan F200 luminometer (Tecan Group Ltd., Männedorf, Switzerland).

### Pathogen inoculation experiments

*Pto* DC3000 COR‐ bacteria were streaked out from glycerol stock on fresh King's B media plates containing 1% agar and 50 μg/ml rifampicin and 50 μg/ml kanamycin. Bacteria were collected from plates with a sterile pipette tip and resuspended in water containing 0.04% Silwet L77 (Sigma Aldrich, St. Louis, USA) to an OD_600_ = 0.2 (10^8^ cfu/ml). The bacterial suspension was sprayed on 4‐ to 5‐week‐old plants, which were subsequently covered with lids for 3 days. Three leaf discs per sample from different plants were collected in microfuge tubes and ground with a tissue lyser (Qiagen, Düsseldorf, Germany). Serial dilutions were plated on LB agar before counting colonies. For inducing resistance with GLV2 peptides, 4‐ to 5‐week‐old plants were infiltrated with 1 μM GLV2 and incubated for 24 h. Subsequently, a suspension of *Pseudomonas* syringae pv. *tomato* DC3000 bacteria was prepared as above to an OD_600_ = 0.0002 (10^5^ colony forming units per mL) and syringe infiltrated into pre‐treated leaves. Two days after inoculation, samples were collected as described above.

### Ethylene measurements

*Arabidopsis* leaf discs (3 mm diameter) were floated overnight in H_2_O‐containing petri dishes. Three leaf discs were transferred to a glass vial (6 ml volume) with 500 μl H_2_O before adding flg22 and/or GLV2 to a final concentration of 500 nM and 1 μM, respectively. Glass vials were tightly sealed with a rubber lid and incubated under slight agitation for 3.5 h on a horizontal shaker. One milliliter air was extracted with a syringe and injected into a gas chromatograph to measure ethylene.

### RNA isolation and quantitative RT‐PCR

Four 2‐week‐old seedlings grown in liquid MS were treated with 100 nM flg22 and/or 1 μM GLV2 for 24 h and ground in liquid N_2_. Total RNA was extracted using Direct‐zol™ RNA Miniprep Plus kit (Zymo Research, Freiburg, Germany) according to the manufacturer`s instructions. cDNA synthesis was performed with 2 μg RNA per sample using RevertAid First Strand cDNA Synthesis kit (Thermo Fisher). *PR1* expression was detected using primers qPR1_F and qPR1_R by quantitative real‐time PCR using Maxima SYBR green mix (Thermo Fisher) and the AriaMx Real‐Time PCR system (Agilent Technologies, Santa Clara, USA). The house keeping gene *UBQ* was used as a reference gene using primers qUBQ_F and qUBQ_R. *FLS2* and *EFR* transcript levels were determined using primers qFLS2_F, qFLS2_R and qEFR_F, qEFR_R, respectively. To detect *RGI1*‐*RGI5* transcript levels in mature leaves of WT and *rgi5x* and *RGI3* expression in *pRGI3::RGI3* lines, primers semi‐qRT‐RGI1‐RGI5_F and R were used. Sequences of all primers can be found in Table S1.

### Root growth and seedling growth inhibition

Seeds were surface sterilized and grown on MS Agar plates for 5 days before transferring individual seedlings in each well of a 48‐well plate containing MS medium with 1 µM GLV2 and/or flg22 in the indicated concentrations before measuring fresh weight 7 days after transfer. To determine root length in *rgi5x* and *rgi5x pRGI3*::*RGI3* lines, seeds were grown vertically on MS Agar plates for 7 days before measuring root length.

### RGI3^ECD^ expression and purification

The ECD construct of RGI3 (RGI3^ECD^) was previously described (Smakowska‐Luzan *et al*, 2018). RGI3^ECD^ was cloned into the baculovirus transfer vector pMeIBac B1 (Invitrogen) using RecA‐mediated sequence and ligation‐independent cloning strategy. A C‐terminal Strep II‐9x His tag was fused to RGI3^ECD^. RGI3^ECD^‐Strep II‐9x His was produced by secreted expression in baculovirus‐infected High Five insect cells and harvested 72 h postinfection. Subsequently, the protein was purified by nickel affinity chromatography (Ni Sepharose excel beads; GE Healthcare) and subjected to a size exclusion chromatography column (Superdex 200 10/300; GE Healthcare) pre‐equilibrated with 20 mM NaH_2_PO_4_/ Na_2_HPO_4_, pH 7.5, 200 mM NaCl, and 5% glycerol.

### Microscale thermophoresis

Purified RGI3^ECD^ was labeled with a fluorescent dye using Monolith Protein Labeling Kit RED‐tris‐NTA Second Generation (His‐tag reactive; NanoTemper Technologies). Fluorescently labeled RGI3^ECD^ (final concentration: 50 nM) was mixed with varying peptide concentrations (ranging from 152.6 pM to 5 μM) in buffer containing 20 mM NaH_2_PO_4_/Na_2_HPO_4_, pH 7.5, 200 mM NaCl, 5% glycerol, and 0.005% Tween. Approximately 8 μl of each sample was loaded in a fused silica capillary (Premium grade, NanoTemper Technologies). Measurements were performed at 25°C in a Monolith NT.115 instrument at a constant LED power of 80% and MST power of 60%. Measurements were performed three times on independent preparations. The data were analyzed by plotting peptide concentrations against percentage changes in normalized fluorescence. Curve fitting was performed using MO. Affinity Analysis (x86) and GraphPad Prism software.

### Protein extraction and co‐immunoprecipitation experiments

To determine FLS2, EFR‐GFP, and BAK1 levels, seedlings were grown for 5 days before transferring two to three seedlings in each well of a 24‐well plate containing MS medium for 7 days. Afterwards, MS medium was replaced with fresh MS medium with or without 1 µM GLV2. Twenty‐four hours later, seedlings were harvested in liquid N_2_ and subjected to protein extraction (50 mM Tris‐HCl pH 7.5, 50 mM NaCl, 10% glycerol, 2 mM ethylenediaminetetraacetic acid, 5 mM dithiothreitol, 1% protease inhibitor cocktail (Sigma Aldrich), 1 mM phenylmethanesulfonyl fluoride, and 1% IGEPAL). After SDS PAGE and western blot, proteins were detected using α‐FLS2, α‐GFP, and α‐BAK1 antibodies (Chinchilla *et al*, 2007).

For co‐immunoprecipitation (Co‐IP) experiments of FLS2‐GFP and BAK1 in Col‐0 pFLS2::FLS2‐GFP lines, proteins were extracted from 12‐day‐old seedlings upon treatment with mock, 1 μM flg22, or co‐treatment with 1 μM flg22 and 1 μM GLV2 for 10 min. For Co‐IP experiments upon transient expression in *N. benthamiana*, 4‐ to 5‐week‐old leaves were infiltrated with Agrobacteria containing constructs for 35S::FLS2‐HA, 35S::CLV1‐GFP, and/or 35S::RGI3‐GFP expression. In all cases constructs were co‐infiltrated with a P19 silencing suppressor construct. Leaves were harvested 2 days postinfiltration and treated with 1 µM flg22 for 30 min via vacuum infiltration before freezing in liquid N_2_. Protein extraction was performed with 50 mM Tris‐HCl pH 7.5, 100 mM NaCl, 10% glycerol, 2 mM ethylenediaminetetraacetic acid, 5 mM dithiothreitol, 1% protease inhibitor cocktail (Sigma Aldrich), 1 mM phenylmethanesulfonyl fluoride, and 0.5% IGEPAL. Immunoprecipitation was performed with magnetic GFP‐TRAP beads (Chromotek, Martinsried, Germany). After SDS PAGE and western blot, proteins were detected using α‐HA (Chromotek), α‐GFP (Santa Cruz Biotechnology, Dallas, USA), and α‐BAK1 antibodies (Chinchilla *et al*, 2007).

### Detection of GLV2 peptides from apoplastic wash fluids using targeted proteomics

Preparations of apoplastic wash fluids were performed as previously described (Nakano *et al*, 2020). In brief, 4‐ to 5‐week‐old soil‐grown plants were detached from root tissue and submerged in apoplastic wash fluid buffer (5 mM NaC_2_H_3_O_2_^‐^, 0.2 M CaCl_2_, pH 4.3) and vacuum infiltrated. Upon centrifugation, eluates were collected and subjected to chlorophenol extraction according to previous protocols (Ohyama *et al*, 2008). After precipitation, peptides were dissolved in H_2_O and analyzed by targeted mass spectrometry using parallel reaction monitoring (PRM). Synthetic GLV2 reference peptide (Table S2) was used for PRM assay setup and optimization. PRM measurements were performed with a 50‐min linear gradient on a Dionex Ultimate 3000 RSLCnano system coupled to a Q‐Exactive HF‐X mass spectrometer (Thermo Fisher Scientific). The mass spectrometer was operated in PRM and positive ionization mode. MS1 spectra (360–1,300 *m/z*) were recorded at a resolution of 60,000 using an AGC target value of 3 × 10^6^ and a MaxIT of 100 ms. Targeted MS2 spectra were acquired at 60,000 resolution with a fixed first mass of 100 *m/z*, after higher‐energy collisional dissociation (HCD) with 26% NCE, an AGC target value of 1 × 10^6^, a MaxIT of 118 ms, and an isolation window of 1.3 m/z. Within a single PRM run, 17 peptide precursors were targeted (the GLV2 target peptide in several charge states as well as 14 retention time reference peptides) without retention time scheduling. The cycle time was ~2.1 s, which lead to ~10 data points per chromatographic peak. PRM data analysis was carried out using the software tool Skyline (version 64‐bit 20.2.0.286 (Maclean *et al*, 2010)). Interferences and peak integration boundaries were reviewed manually, considering the 10 most intense transitions (precursor–fragment ion pairs) of the GLV2 peptide. The total GLV2 peptide intensity was computed by summing up all transition intensities.

### Synthetic peptides and elicitors

The flg22 and elf18 peptides were kindly provided by Dr. Justin Lee (IPB Halle). GLV2 and GLV^‐S^ peptides were synthesized by Pepmic (Suzhou, China) to a purity of minimum 90%. All peptides were dissolved in H_2_O. Sequences of all peptides used in this study can be found in Appendix Table S3.

### Statistical analysis

Analysis was carried out using the GraphPad Prism software (San Diego, USA). The detailed statistical method employed is provided in the respective figure legends.

## Author contributions

**Martin Stegmann:** Conceptualization; Formal analysis; Supervision; Investigation; Visualization; Methodology; Writing—original draft; Project administration. **Patricia Zecua‐Ramirez:** Investigation; Writing—review & editing. **Christina Ludwig:** Investigation; Writing—review & editing. **Ho‐Seok Lee:** Investigation; Methodology; Writing—review & editing. **Brenda Peterson:** Investigation. **Zachary Nimchuk:** Resources; Supervision; Writing—review & editing. **Youssef Belkhadir:** Resources; Supervision; Funding acquisition; Writing—review & editing. **Ralph Hückelhoven:** Resources; Supervision; Funding acquisition; Writing—review & editing.

In addition to the CRediT author contributions listed above, the contributions in detail are:

MS planned the project. MS, YB, CL, and RH designed and conceived the experiments and analyzed data. ZLN oversaw work. MS, PZ‐R, CL, H‐SL, and BP performed the experiments. MS wrote the manuscript with input from all the authors.

## Disclosure and competing interests statement

The authors declare that they have no conflict of interest.

## Supporting information



Appendix

Expanded View Figures PDF

Source Data for Expanded View

Source Data for Figure 1

Source Data for Figure 2

Source Data for Figure 3

Source Data for Figure 4

Source Data for Figure 5

Source Data for Figure 6

## Data Availability

All targeted proteomic raw data and the Skyline analysis file have been deposited to Panorama Public (Sharma *et al*, 2018) and can be accessed under https://panoramaweb.org/GLV2_peptide.url (ProteomeXchange ID: PXD023855).

## References

[embr202153281-bib-0001] Bartels S , Boller T (2015) Quo vadis, pep? Plant elicitor peptides at the crossroads of immunity, stress and development. J Exp Bot 66: 5183–5193 25911744 10.1093/jxb/erv180

[embr202153281-bib-0002] Bartels S , Lori M , Mbengue M , van Verk M , Klauser D , Hander T , Böni R , Robatzek S , Boller T , Van VM *et al* (2013) The family of Peps and their precursors in *Arabidopsis*: differential expression and localization but similar induction of pattern‐triggered immune responses. J Exp Bot 64: 5309–5321 24151300 10.1093/jxb/ert330

[embr202153281-bib-0003] Beck M , Zhou J , Faulkner C , Maclean D , Robatzek S (2012) Spatio‐temporal cellular dynamics of the *Arabidopsis* flagellin receptor reveal activation status‐dependent endosomal sorting. Plant Cell 24: 4205–4219 23085733 10.1105/tpc.112.100263 PMC3516521

[embr202153281-bib-0004] Bernsdorff F , Döring AC , Gruner K , Schuck S , Bräutigam A , Zeier J (2016) Pipecolic acid orchestrates plant systemic acquired resistance and defense priming via salicylic acid‐dependent and ‐independent pathways. Plant Cell 28: 102–129 26672068 10.1105/tpc.15.00496 PMC4746677

[embr202153281-bib-0005] Buscaill P , Chandrasekar B , Sanguankiattichai N , Kourelis J , Kaschani F , Thomas EL , Morimoto K , Kaiser M , Preston GM , Ichinose Y *et al* (2019) Glycosidase and glycan polymorphism control hydrolytic release of immunogenic flagellin peptides. Science 364: eaav0748 30975858 10.1126/science.aav0748

[embr202153281-bib-0006] Chinchilla D , Zipfel C , Robatzek S , Kemmerling B , Nürnberger T , Jones JDG , Felix G , Boller T (2007) A flagellin‐induced complex of the receptor FLS2 and BAK1 initiates plant defence. Nature 448: 497–500 17625569 10.1038/nature05999

[embr202153281-bib-0007] Couto D , Zipfel C (2016) Regulation of pattern recognition receptor signalling in plants. Nat Rev Immunol 16: 537–552 27477127 10.1038/nri.2016.77

[embr202153281-bib-0008] DeFalco TA , Zipfel C (2021) Molecular mechanisms of early plant pattern‐triggered immune signaling. Mol Cell 81: 3449–3467 34403694 10.1016/j.molcel.2021.07.029

[embr202153281-bib-0009] Engler C , Kandzia R , Marillonnet S (2008) A one pot, one step, precision cloning method with high throughput capability. PLoS One 3: e3647 18985154 10.1371/journal.pone.0003647 PMC2574415

[embr202153281-bib-0010] Fernandez A , Drozdzecki A , Hoogewijs K , Nguyen A , Beeckman T , Madder A , Hilson P (2013a) Transcriptional and functional classification of the GOLVEN/ROOT GROWTH FACTOR/CLE‐like signaling peptides reveals their role in lateral root and hair formation. Plant Physiol 161: 954–970 23370719 10.1104/pp.112.206029 PMC3561032

[embr202153281-bib-0011] Fernandez A , Drozdzecki A , Hoogewijs K , Vassileva V , Madder A , Beeckman T , Hilson P (2015) The GLV6/RGF8/CLEL2 peptide regulates early pericycle divisions during lateral root initiation. J Exp Bot 66: 5245–5256 26163695 10.1093/jxb/erv329 PMC4526922

[embr202153281-bib-0012] Fernandez A , Hilson P , Beeckman T (2013b) Golven peptides as important regulatory signalling molecules of plant development. J Exp Bot 64: 5263–5268 23975768 10.1093/jxb/ert248

[embr202153281-bib-0013] Fernandez AI , Vangheluwe N , Xu KE , Jourquin J , Claus LAN , Morales‐Herrera S , Parizot B , De Gernier H , Yu Q , Drozdzecki A *et al* (2020) GOLVEN peptide signalling through RGI receptors and MPK6 restricts asymmetric cell division during lateral root initiation. Nat Plants 6: 533–543 32393883 10.1038/s41477-020-0645-z

[embr202153281-bib-0014] Flury P , Klauser D , Schulze B , Boller T , Bartels S (2013) The anticipation of danger: microbe‐associated molecular pattern perception enhances AtPep‐triggered oxidative burst. Plant Physiol 161: 2023–2035 23400703 10.1104/pp.113.216077 PMC3613473

[embr202153281-bib-0015] Ghorbani S , Hoogewijs K , Pečenková T , Fernandez A , Inzé A , Eeckhout D , Kawa D , De Jaeger G , Beeckman T , Madder A *et al* (2016) The SBT6.1 subtilase processes the GOLVEN1 peptide controlling cell elongation. J Exp Bot 67: 4877–4887 27315833 10.1093/jxb/erw241 PMC4983112

[embr202153281-bib-0016] Göhre V , Spallek T , Häweker H , Mersmann S , Mentzel T , Boller T , de Torres M , Mansfield JW , Robatzek S (2008) Plant pattern‐recognition receptor FLS2 is directed for degradation by the bacterial ubiquitin ligase AvrPtoB. Curr Biol 18: 1824–1832 19062288 10.1016/j.cub.2008.10.063

[embr202153281-bib-0017] Gómez‐Gómez L , Boller T (2000) FLS2: an LRR receptor‐like kinase involved in the perception of the bacterial elicitor flagellin in *Arabidopsis* . Mol Cell 5: 1003–1011 10911994 10.1016/s1097-2765(00)80265-8

[embr202153281-bib-0018] Gully K , Pelletier S , Guillou M‐C , Ferrand M , Aligon S , Pokotylo I , Perrin A , Vergne E , Fagard M , Ruelland E *et al* (2019) The SCOOP12 peptide regulates defense response and root elongation in *Arabidopsis thaliana* . J Exp Bot 70: 1349–1365 30715439 10.1093/jxb/ery454 PMC6382344

[embr202153281-bib-0019] Gust AA , Pruitt R , Nürnberger T (2017) Sensing danger: Key to activating plant immunity. Trends Plant Sci 22: 779–791 28779900 10.1016/j.tplants.2017.07.005

[embr202153281-bib-0020] Hander T , Fernandez‐Fernandez AD , Kumpf RP , Schatowitz H , Pottie R , Rombaut D , Staes A , Nolf J , Boller T , Gevaert K *et al* (2019) Damage on plants activates Ca^2+^‐dependent metacaspases for release of plant immunomodulatory peptides. Science 363: eear7486 10.1126/science.aar7486 30898901

[embr202153281-bib-0021] Heese A , Hann DR , Gimenez‐Ibanez S , Jones AME , He K , Li J , Schroeder JI , Peck SC , Rathjen JP (2007) The receptor‐like kinase SERK3/BAK1 is a central regulator of innate immunity in plants. Proc Natl Acad Sci USA 104: 12217–12222 17626179 10.1073/pnas.0705306104 PMC1924592

[embr202153281-bib-0022] Hou S , Liu D , Huang S , Luo D , Liu Z , Xiang Q , Wang P , Mu R , Han Z , Chen S *et al* (2021) The *Arabidopsis* MIK2 receptor elicits immunity by sensing a conserved signature from phytocytokines and microbes. Nat Commun 12: 25580 10.1038/s41467-021-25580-w PMC8448819 34535661

[embr202153281-bib-0023] Hou S , Wang X , Chen D , Yang X , Wang M , Turrà D , Di Pietro A , Zhang W (2014) The secreted peptide PIP1 amplifies immunity through receptor‐like kinase 7. PLoS Pathog 10: e1004331 25188390 10.1371/journal.ppat.1004331 PMC4154866

[embr202153281-bib-0024] Howard BE , Hu Q , Babaoglu AC , Chandra M , Borghi M , Tan X , He L , Winter‐Sederoff H , Gassmann W , Veronese P *et al* (2013) High‐throughput RNA sequencing of *Pseudomonas*‐infected *Arabidopsis* reveals hidden transcriptome complexity and novel splice variants. PLoS One 8: e74183 24098335 10.1371/journal.pone.0074183 PMC3788074

[embr202153281-bib-0025] Igarashi D , Tsuda K , Katagiri F (2012) The peptide growth factor, phytosulfokine, attenuates pattern‐triggered immunity. Plant J 71: 194–204 22353039 10.1111/j.1365-313X.2012.04950.x

[embr202153281-bib-0026] Kaufmann C , Sauter M (2019) Sulfated plant peptide hormones. J Exp Bot 70: 4267–4277 31231771 10.1093/jxb/erz292 PMC6698702

[embr202153281-bib-0027] Klepikova AV , Kasianov AS , Gerasimov ES , Logacheva MD , Penin AA (2016) A high resolution map of the *Arabidopsis thaliana* developmental transcriptome based on RNA‐seq profiling. Plant J 88: 1058–1070 27549386 10.1111/tpj.13312

[embr202153281-bib-0028] Kunze G , Zipfel C , Robatzek S , Niehaus K , Boller T , Felix G (2004) The N terminus of bacterial elongation factor Tu elicits innate immunity in *Arabidopsis* plants. Plant Cell 16: 3496–3507 15548740 10.1105/tpc.104.026765 PMC535888

[embr202153281-bib-0029] Li J , Zhao‐Hui C , Batoux M , Nekrasov V , Roux M , Chinchilla D , Zipfel C , Jones JDG (2009) Specific ER quality control components required for biogenesis of the plant innate immune receptor EFR. Proc Natl Acad Sci USA 106: 15973–15978 19717464 10.1073/pnas.0905532106 PMC2747228

[embr202153281-bib-0030] Lu D , Lin W , Gao X , Wu S , Cheng C , Avila J , Heese A , Devarenne TP , He P , Shan L (2011) Direct ubiquitination of pattern recognition receptor FLS2 attenuates plant innate immunity. Science 332: 1439–1442 21680842 10.1126/science.1204903 PMC3243913

[embr202153281-bib-0031] Lu X , Shi H , Ou Y , Cui Y , Chang J , Peng L , Gou X , He K , Li J (2020) RGF1‐RGI1, a peptide‐receptor complex, regulates *Arabidopsis* root meristem development via a MAPK signaling cascade. Mol Plant 13: 1594–1607 32916335 10.1016/j.molp.2020.09.005

[embr202153281-bib-0032] Luu DD , Joe A , Chen Y , Parys K , Bahar O , Pruitt R , Chan LJG , Petzold CJ , Long K , Adamchak C *et al* (2019) Biosynthesis and secretion of the microbial sulfated peptide RaxX and binding to the rice XA21 immune receptor. Proc Natl Acad Sci USA 116: 8525 30948631 10.1073/pnas.1818275116 PMC6486716

[embr202153281-bib-0033] Ma X , Xu G , He P , Shan L (2016) SERKing coreceptors for receptors. Trends Plant Sci 21: 1017–1033 27660030 10.1016/j.tplants.2016.08.014

[embr202153281-bib-0034] Maclean B , Tomazela DM , Shulman N , Chambers M , Finney GL , Frewen B , Kern R , Tabb DL , Liebler DC , Maccoss MJ (2010) Skyline: an open source document editor for creating and analyzing targeted proteomics experiments. Bioinformatics 26: 966–968 20147306 10.1093/bioinformatics/btq054 PMC2844992

[embr202153281-bib-0035] Matsuzaki Y , Ogawa‐Ohnishi M , Mori A , Matsubayashi Y (2010) Secreted peptide signals required for maintenance of root stem cell niche in *Arabidopsis* . Science 329: 1065–1067 20798316 10.1126/science.1191132

[embr202153281-bib-0036] Mbengue M , Bourdais G , Gervasi F , Beck M , Zhou JI , Spallek T , Bartels S , Boller T , Ueda T , Kuhn H *et al* (2016) Clathrin‐dependent endocytosis is required for immunity mediated by pattern recognition receptor kinases. Proc Natl Acad Sci USA 113: 11034–11039 27651493 10.1073/pnas.1606004113 PMC5047200

[embr202153281-bib-0037] Meng L , Buchanan BB , Feldman LJ , Luan S (2012) CLE‐like (CLEL) peptides control the pattern of root growth and lateral root development in *Arabidopsis* . Proc Natl Acad Sci USA 109: 1760–1765 22307643 10.1073/pnas.1119864109 PMC3277184

[embr202153281-bib-0038] Mosher S , Seybold H , Rodriguez P , Stahl M , Davies KA , Dayaratne S , Morillo SA , Wierzba M , Favery B , Keller H *et al* (2013) The tyrosine‐sulfated peptide receptors PSKR1 and PSY1R modify the immunity of *Arabidopsis* to biotrophic and necrotrophic pathogens in an antagonistic manner. Plant J 73: 469–482 23062058 10.1111/tpj.12050

[embr202153281-bib-0039] Nakagawa T , Kurose T , Hino T , Tanaka K , Kawamukai M , Niwa Y , Toyooka K , Matsuoka K , Jinbo T , Kimura T (2007) Development of series of gateway binary vectors, pGWBs, for realizing efficient construction of fusion genes for plant transformation. J Biosci Bioeng 104: 34–41 17697981 10.1263/jbb.104.34

[embr202153281-bib-0040] Nakano RT , Ishihama N , Wang Y , Takagi J , Uemura T , Schulze‐lefert P , Nakagami H (2020) Apoplastic fluid preparation from *Arabidopsis thaliana* mildew leaves upon interaction with a nonadapted powdery mildew pathogen. Plant Proteomics 2139: 79–88 10.1007/978-1-0716-0528-8_6 32462579

[embr202153281-bib-0041] Nekrasov V , Li J , Batoux M , Roux M , Chu Z‐H , Lacombe S , Rougon A , Bittel P , Kiss‐Papp M , Chinchilla D *et al* (2009) Control of the pattern‐recognition receptor EFR by an ER protein complex in plant immunity. EMBO J 28: 3428–3438 19763086 10.1038/emboj.2009.262 PMC2776097

[embr202153281-bib-0042] Ohyama K , Ogawa M , Matsubayashi Y (2008) Identification of a biologically active, small, secreted peptide in *Arabidopsis* by in silico gene screening, followed by LC‐MS‐based structure analysis. Plant J 55: 152–160 18315543 10.1111/j.1365-313X.2008.03464.x

[embr202153281-bib-0043] Ou Y , Lu X , Zi Q , Xun Q , Zhang J , Wu Y , Shi H , Wei Z , Zhao B , Zhang X *et al* (2016) RGF1 INSENSITIVE 1 to 5, a group of LRR receptor‐like kinases, are essential for the perception of root meristem growth factor 1 in *Arabidopsis thaliana* . Cell Res 26: 686–698 27229312 10.1038/cr.2016.63 PMC4897188

[embr202153281-bib-0044] Rhodes J , Yang H , Moussu S , Boutrot F , Santiago J , Zipfel C (2021) Perception of a divergent family of phytocytokines by the *Arabidopsis* receptor kinase MIK2. Nat Commun 12: 5494 33514716 10.1038/s41467-021-20932-y PMC7846792

[embr202153281-bib-0045] Robatzek S , Chinchilla D , Boller T (2006) Ligand‐induced endocytosis of the pattern recognition receptor FLS2 in *Arabidopsis* . Genes Dev 20: 537–542 16510871 10.1101/gad.366506 PMC1410809

[embr202153281-bib-0046] Roux M , Schwessinger B , Albrecht C , Chinchilla D , Jones A , Holton N , Malinovsky FG , Tör M , de Vries S , Zipfel C (2011) The *Arabidopsis* leucine‐rich repeat receptor‐like kinases BAK1/SERK3 and BKK1/SERK4 are required for innate immunity to hemibiotrophic and biotrophic pathogens. Plant Cell 23: 2440–2455 21693696 10.1105/tpc.111.084301 PMC3160018

[embr202153281-bib-0047] Saijo Y , Tintor N , Lu X , Rauf P , Pajerowska‐Mukhtar K , Häweker H , Dong X , Robatzek S , Schulze‐Lefert P (2009) Receptor quality control in the endoplasmic reticulum for plant innate immunity. EMBO J 28: 3439–3449 19763087 10.1038/emboj.2009.263 PMC2776098

[embr202153281-bib-0048] Segonzac C , Monaghan J (2019) Modulation of plant innate immune signaling by small peptides. Curr Opin Plant Biol 51: 22–28 31026543 10.1016/j.pbi.2019.03.007

[embr202153281-bib-0049] Shao Y , Yu X , Xu X , Li Y , Yuan W , Xu Y , Mao C , Zhang S , Xu J (2020) The YDA‐MKK4/MKK5‐MPK3/MPK6 cascade functions downstream of the RGF1‐RGI ligand‐receptor pair in regulating mitotic activity in the root apical meristem. Mol Plant 13: 1608–1623 32916336 10.1016/j.molp.2020.09.004

[embr202153281-bib-0050] Sharma V , Eckels J , Schilling B , Ludwig C , Jaffe JD , Maccoss MJ , Maclean B (2018) Panorama public: a public repository for quantitative data sets processed in skyline*. Mol Cell Proteomics 17: 1239–1244 29487113 10.1074/mcp.RA117.000543 PMC5986241

[embr202153281-bib-0051] Shen Q , Bourdais G , Pan H , Robatzek S , Tang D (2017) *Arabidopsis* glycosylphosphatidylinositol‐anchored protein LLG1 associates with and modulates FLS2 to regulate innate immunity. Proc Natl Acad Sci USA 114: 5749–5754 28507137 10.1073/pnas.1614468114 PMC5465910

[embr202153281-bib-0052] Shinohara H , Mori A , Yasue N , Sumida K , Matsubayashi Y (2016) Identification of three LRR‐RKs involved in perception of root meristem growth factor in *Arabidopsis* . Proc Natl Acad Sci USA 113: 3897–3902 27001831 10.1073/pnas.1522639113 PMC4833249

[embr202153281-bib-0065] Smakowska‐Luzan E , Mott GA , Parys K , Stegmann M , Howton TC , Layeghifard M , Neuhold J , Lehner A , Kong J , Grünwald K *et al* (2018) An extracellular network of Arabidopsis leucine‐rich repeat receptor kinases. Nature 553: 342–346 29320478 10.1038/nature25184 PMC6485605

[embr202153281-bib-0053] Song W , Liu LI , Wang J , Wu Z , Zhang H , Tang J , Lin G , Wang Y , Wen X , Li W *et al* (2016) Signature motif‐guided identification of receptors for peptide hormones essential for root meristem growth. Cell Res 26: 674–685 27229311 10.1038/cr.2016.62 PMC4897187

[embr202153281-bib-0054] Srivastava R , Liu J‐X , Guo H , Yin Y , Howell SH (2008) Regulation and processing of a plant peptide hormone, AtRALF23, in *Arabidopsis* . Plant J 59: 930–939 10.1111/j.1365-313X.2009.03926.x 19473327

[embr202153281-bib-0055] Stegmann M , Monaghan J , Smakowska‐Luzan E , Rovenich H , Lehner A , Holton N , Belkhadir Y , Zipfel C (2017) The receptor kinase FER is a RALF‐regulated scaffold controlling plant immune signaling. Science 355: 287–289 28104890 10.1126/science.aal2541

[embr202153281-bib-0056] Stührwohldt N , Scholl S , Lang L , Katzenberger J , Schumacher K , Schaller A (2020) The biogenesis of CLEL peptides involves several processing events in consecutive compartments of the secretory pathway. Elife 9: e55580 32297855 10.7554/eLife.55580 PMC7162652

[embr202153281-bib-0057] Tintor N , Ross A , Kanehara K , Yamada K , Fan L , Kemmerling B , Nürnberger T , Tsuda K , Saijo Y (2013) Layered pattern receptor signaling via ethylene and endogenous elicitor peptides during *Arabidopsis* immunity to bacterial infection. Proc Natl Acad Sci USA 110: 6211–6216 23431187 10.1073/pnas.1216780110 PMC3625345

[embr202153281-bib-0058] Trinchieri G , Sher A (2007) Cooperation of Toll‐like receptor signals in innate immune defence. Nat Rev Immunol 7: 179–190 17318230 10.1038/nri2038

[embr202153281-bib-0059] Wang X , Zhang N , Zhang L , He Y , Cai C , Zhou J , Li J , Meng X (2021) Perception of the pathogen‐induced peptide RGF7 by the receptor‐like kinases RGI4 and RGI5 triggers innate immunity in *Arabidopsis thaliana* . New Phytol 230: 1110–1125 33454976 10.1111/nph.17197

[embr202153281-bib-0060] Whitford R , Fernandez A , Tejos R , Pérez A , Kleine‐Vehn J , Vanneste S , Drozdzecki A , Leitner J , Abas L , Aerts M *et al* (2012) GOLVEN secretory peptides regulate auxin carrier turnover during plant gravitropic responses. Dev Cell 22: 678–685 22421050 10.1016/j.devcel.2012.02.002

[embr202153281-bib-0061] Xiao Y , Stegmann M , Han Z , DeFalco TA , Parys K , Xu L , Belkhadir Y , Zipfel C , Chai J (2019) Mechanisms of RALF peptide perception by a heterotypic receptor complex. Nature 572: 270–274 31291642 10.1038/s41586-019-1409-7

[embr202153281-bib-0062] Yamada M , Han X , Benfey PN (2019) RGF1 controls root meristem size through ROS signalling. Nature 577: 85–88 31801996 10.1038/s41586-019-1819-6 PMC6930331

[embr202153281-bib-0063] Zipfel C , Kunze G , Chinchilla D , Caniard A , Jones JDG , Boller T , Felix G (2006) Perception of the bacterial PAMP EF‐Tu by the receptor EFR restricts *Agrobacterium*‐mediated transformation. Cell 125: 749–760 16713565 10.1016/j.cell.2006.03.037

[embr202153281-bib-0064] Zipfel C , Robatzek S , Navarro L , Oakeley EJ , Jones JDG , Felix G , Boller T (2004) Bacterial disease resistance in *Arabidopsis* through flagellin perception. Nature 428: 764–767 15085136 10.1038/nature02485

