# Supplementary material for: RGI‐GOLVEN signaling promotes cell surface immune receptor abundance to regulate plant immunity
Source: EMBO Rep. 2022 Mar 1;23(5):e53281. doi: 10.15252/embr.202153281 (PMC9066070; doi:10.15252/embr.202153281)
Supplement: Supplementary file 1 — Appendix [file EMBR-23-e53281-s003.docx]

**Appendix for**

**RGI-GOLVEN signaling promotes cell surface immune receptor abundance to regulate plant immunity**

This PDF file includes:

Appendix Table S1 (page 2)

Appendix Table S2 (page 3)

Appendix Table S3 (page 4)

**Appendix Table S1: Different nomenclature of GLV peptides and corresponding receptors**

| **AGI code** | **GLV nomenclature (Whitford et al., 2012)** | **RGF nomenclature (Matsuzaki et al., 2010)** | **CLEL nomenclature (Meng et al., 2012)** | **Genetic GLV- receptor interaction** | **Biochemical GLV-receptor interaction** |
| --- | --- | --- | --- | --- | --- |
| AT4G16515 | GLV1 | RGF6 | CLEL6 | - | - |
| **AT5G64770** | **GLV2** | **RGF9** | **CLEL9** | **RGI3/RGFR3 (this study)** | **RGI3/RGFR3 (this study)** |
| AT3G30350 | GLV3 | RGF4 | - | - | RGI1/RGFR1, RGI2/RGFR2 (Song et al., 2016, Shinohara et al., 2016) |
| AT3G02240 | GLV4 | RGF7 | CLEL4 | - | - |
| AT1G13620 | GLV5 | RGF2 | CLEL1 |  | RGI1/RGFR1, RGI2/RGFR2 (Song et al., 2016, Shinohara et al., 2016) |
| AT2G03830 | GLV6 | RGF8 | CLEL2 | RGI1/RGFR1, RGI4/RGFR4, RGI5/RGFR5 (Fernandez et al., 2020) | RGI1/RGFR1, RGI2/RGFR2 (Song et al., 2016, Shinohara et al., 2016) |
| AT2G04025 | GLV7 | RGF3 | CLEL3 | - | RGI1/RGFR1, RGI2/RGFR2 (Song et al., 2016, Shinohara et al., 2016) |
| AT3G02242 | GLV8 | - | CLEL5 | - | - |
| AT5G15725 | GLV9 | - | - | - | - |
| AT5G51451 | GLV10 | RGF5 | CLEL7 | - | RGI1/RGFR1, RGI2/RGFR2 (Song et al., 2016, Shinohara et al., 2016) |
| AT5G60810 | GLV11 | RGF1 | CLEL8 | RGI1-5/RGFR1-5 (Song et al., 2016; Ou et al., 2016; Shinohara et al., 2016) | RGI1-5/RGFR1-5 (Song et al., 2016; Ou et al., 2016; Shinohara et al., 2016) |
| AT1G66145 | - | - | CLE18 | - | - |

**Appendix Table S2: Primers used in this study**

| **Primer name** | **sequence** |
| --- | --- |
| pRGI3_F | GGGGACAAGTTTGTACAAAAAAGCAGGCTTTCCACTATTTACCCACTTCTG |
| pRGI3_R | GGGGACCACTTTGTACAAGAAAGCTGGGTCAACGGAGTCATCCGAAAAC |
| RGI3_F | GGGGACAAGTTTGTACAAAAAAGCAGGCTTTTATGCCACCAAATATCTATAGAC |
| RGI3_R | GGGGACCACTTTGTACAAGAAAGCTGGGTCAACGGAGTCATCCGAAAAC. |
| CLV1_F | TTTCACCTGCAAAAAATGGCGATGAGAC |
| CLV1_R | TTTCACCTGCTTGGACTAGAACGCGATCAAGTTC |
| CLV1-AarI_F | GGCGAGATTCCACCCGCGATTGGTAATTTCCCC |
| CLV1-AarI_R | GGAAATTACCAATCGCGGGTGGAATCTCGCCGG |
| FLS2_1_F | TTTCGTCTCCAATGAAGTTACTCTCAAAGACC |
| FLS2_1_R | TTTCGTCTCTCTGCCGGGAGCTCACCGG |
| FLS2_2_F | TTTCGTCTCGGCAGATCTAGGGCTTCTTAC |
| FLS2_2_R | TTTCGTCTCTACTAGACTTCTCGATCCTCGTTAC |
| FLS2-AarI_F | ATTGGACCGGAATCACTTGCGATAGTACCGGAC |
| FLS2-AarI_R | GTCCGGTACTATCGCAAGTGATTCCGGTCCAAT |
| BAK1_F | TGAAGACTTAATGGAACGAAGATTAATGATCC |
| BAK1_R | TGAAGACATACTATCTTGGACCCGAGGGG |
| Semi-qRT-RGI3_F | TTTACAATCCCAACGCACTTC |
| Semi-qRT-RGI3_R | CGTGCAATTCGTTAGCTCTTC |
| Semi-qRT-RGI4_F | TTCCAGTTCCGATCACGTTAG |
| Semi-qRT-RGI4_R | GTCAAGAGCTTCAAGCGATTG |
| Semi-qRT-RGI5_F | CCCACCAATCATTTCAACAAC |
| Semi-qRT-RGI5_R | TGAGGTTTCCAATTTGTGAGG |
| Semi-qRT-RGI1_F | GGTAATTGCAGCTCTTTGGTG |
| Semi-qRT-RGI1_R | TTCCAACAACATCCGAGAAAC |
| Semi-qRT-RGI2_F | AGTTGGATATTCCATGGGAGC |
| Semi-qRT-RGI2_R | CGATAGCACATGTGATGGATG |
| qPR1_F | CGGAGCTACGCAGAACAACT |
| qPR1_R | CAGACAAGTCACCGCTACCC |
| qFLS2_F | TGCTCACGTAAGCGATTTTG |
| qFLS2_R | CTGTTTCGTCATCAGCTCCA |
| qEFR_F | TTGTGGCTTCTCTGTGTTGG |
| qEFR_R | TTACCGAAATTGCCTGAACC |
| qUBQ_F | CCAAGCCGAAGAAGATCAAG |
| qUBQ_R | ACTCCTTCCTCAAACGCTGA |

**Appendix Table S3: Sequences of peptides used in this study**

| **Name** | **sequence** |
| --- | --- |
| flg22 | Ac-QRLSTGSRINSAKDDAAGLQIA |
| GLV2 | DMD(TyrSO_3_H_2_)NSANKKR(Hyp)IHN |
| GLV2^-S^ | DMDYNSANKKR(Hyp)IHN |
