# Supplementary material for: RGI‐GOLVEN signaling promotes cell surface immune receptor abundance to regulate plant immunity
Source: EMBO Rep. 2022 Mar 1;23(5):e53281. doi: 10.15252/embr.202153281 (PMC9066070; doi:10.15252/embr.202153281)
Supplement: Supplementary file 2 — Expanded View Figures PDF [file EMBR-23-e53281-s001.pdf]

Expanded View Figures

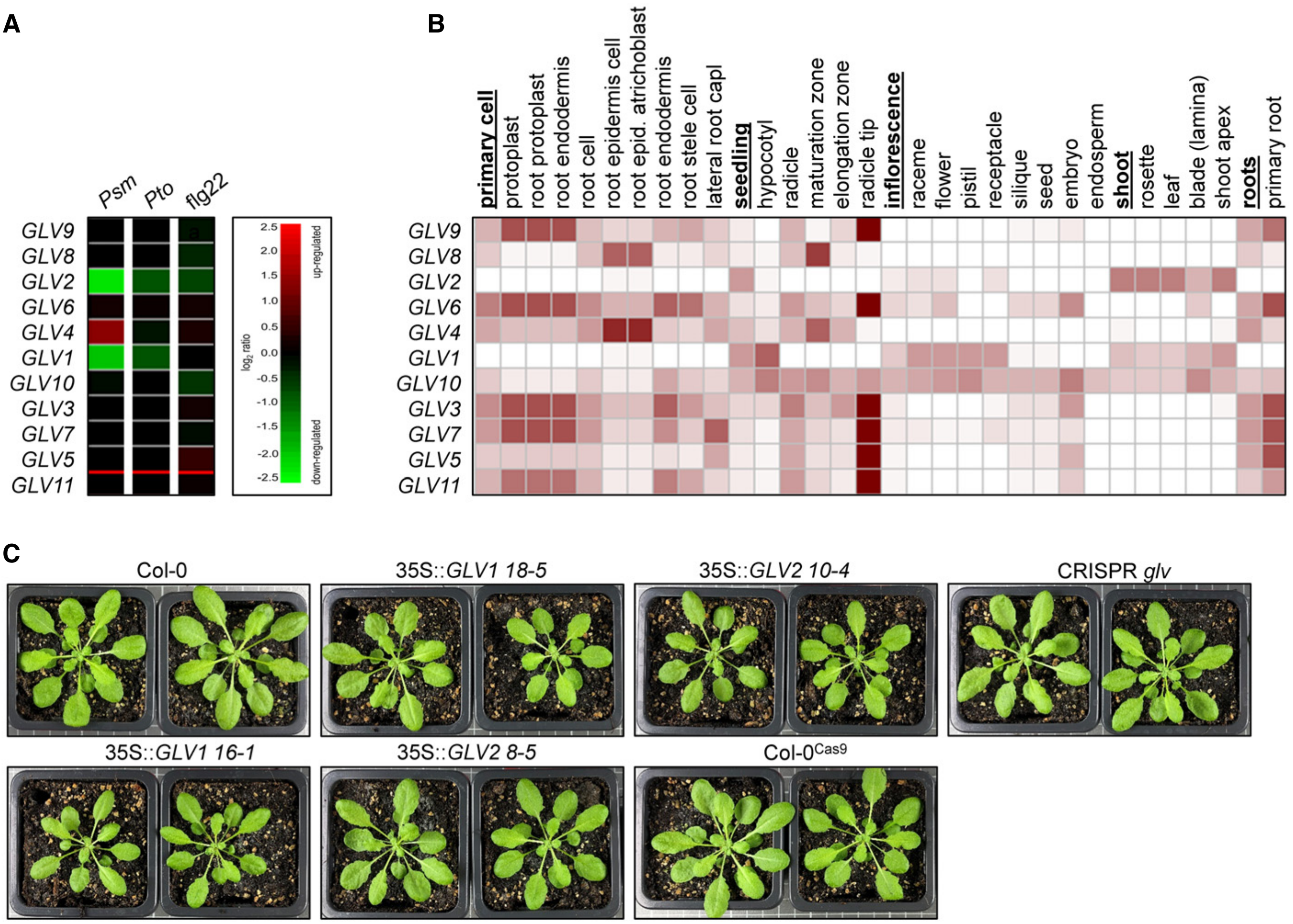

Figure EV1. *GLV* peptide differential expression and morphology of *GLV* overexpression lines and CRISPR *glv*.

A Both *GLV1* and *GLV2* are transcriptionally downregulated after infection with *Psm* and to a lesser extent after infection with *Pto*. Treatment with flg22 does not affect *GLV* gene expression. Data were obtained using Genevestigator software and are based on the AT_nRNASeq_ARABI_GL-1 data set.

B Tissue-specific expression pattern of *GLV* family members. *GLV2* is predicted to be the strongest expressed *GLV* member in *Arabidopsis* mature leaves. Data were obtained using Genevestigator software and are based on the AT_mRNASeq_ARABI_GL-1 data set.

C Pictures of 6-week-old plants of the indicated genotypes.

 © 2022 The Authors

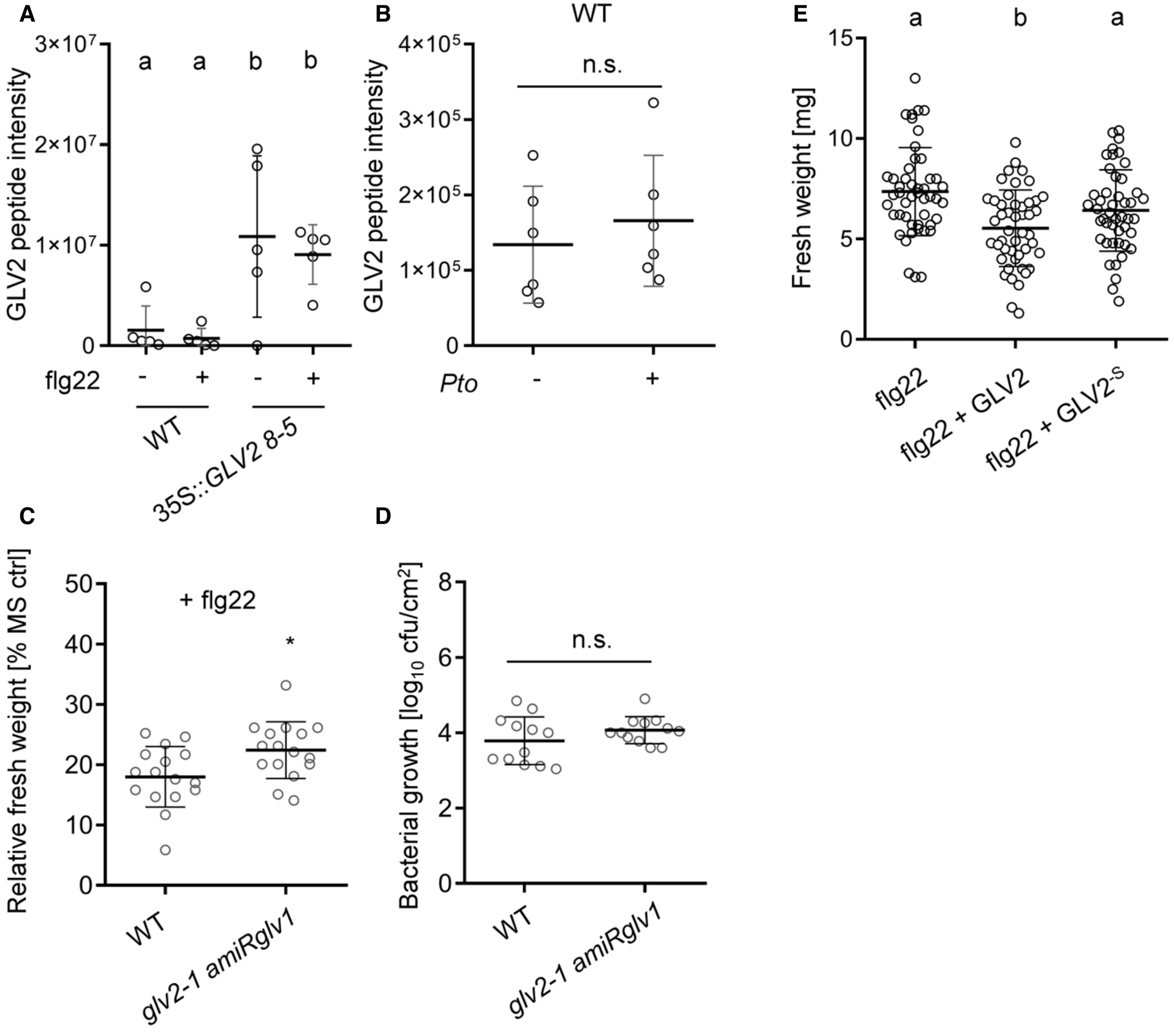

Figure EV2. GLV2 peptide detection, phenotypic characterization of *glv1 glv2*, and requirement of GLV2 tyrosine sulfation for its biological activity.

A, B Targeted proteomics (parallel reaction monitoring, PRM) detection of the GLV2 peptide (DMDY[+80]NSANKKRP[+16]IHN) from apoplastic wash fluids of the indicated genotypes after treatment with 1 h mock or 100 nM flg22 (A) or 24 h postinfection with *Pto* (B). An optimized targeted proteomics assay was setup using a synthetic reference GLV2 peptide (Appendix Table S3). Shown are label-free mean peptide intensities. The standard deviation is indicated from intensities ± SD, n = 5–6 biological replicates (one-way ANOVA, Dunnett *post hoc* test, a-b $P < 0.05$).

C Five-day-old seedlings of the indicated genotypes were treated with 100 nM flg22 for 7 days before measuring fresh weight. Shown is the mean of relative fresh weight compared to the MS control ± SD, n = 16 biological replicates (Student's *t*-test, *$P < 0.05$). The *glv2-1 amiRglv1* lines show reduced flg22-triggered seedling growth inhibition. Similar results were obtained in three independent experiments.

D Colony forming units (cfu) of *Pto* DC3000 COR- 4 days after spray infection of the indicated genotypes; n = 12 biological replicates from three pooled experiments ± SD. Student's *t*-test indicates $P = 0.19$. The *glv2-1 amiRglv1* lines do not show a significantly altered infection phenotype.

E 5-day-old WT seedlings were treated with 10 nM flg22 or co-treated with 1 μM GLV2 or 1 μM GLV2^{-S} for 7 days before measuring fresh weight. Shown is the mean of relative fresh weight compared to the MS medium control ± SD, n = 45–47 biological replicates from four pooled experiments (one-way ANOVA, a-b $P < 0.001$). The GLV2^{-S} mutant peptide does not significantly enhance flg22-induced seedling growth inhibition.

Source data are available online for this figure.

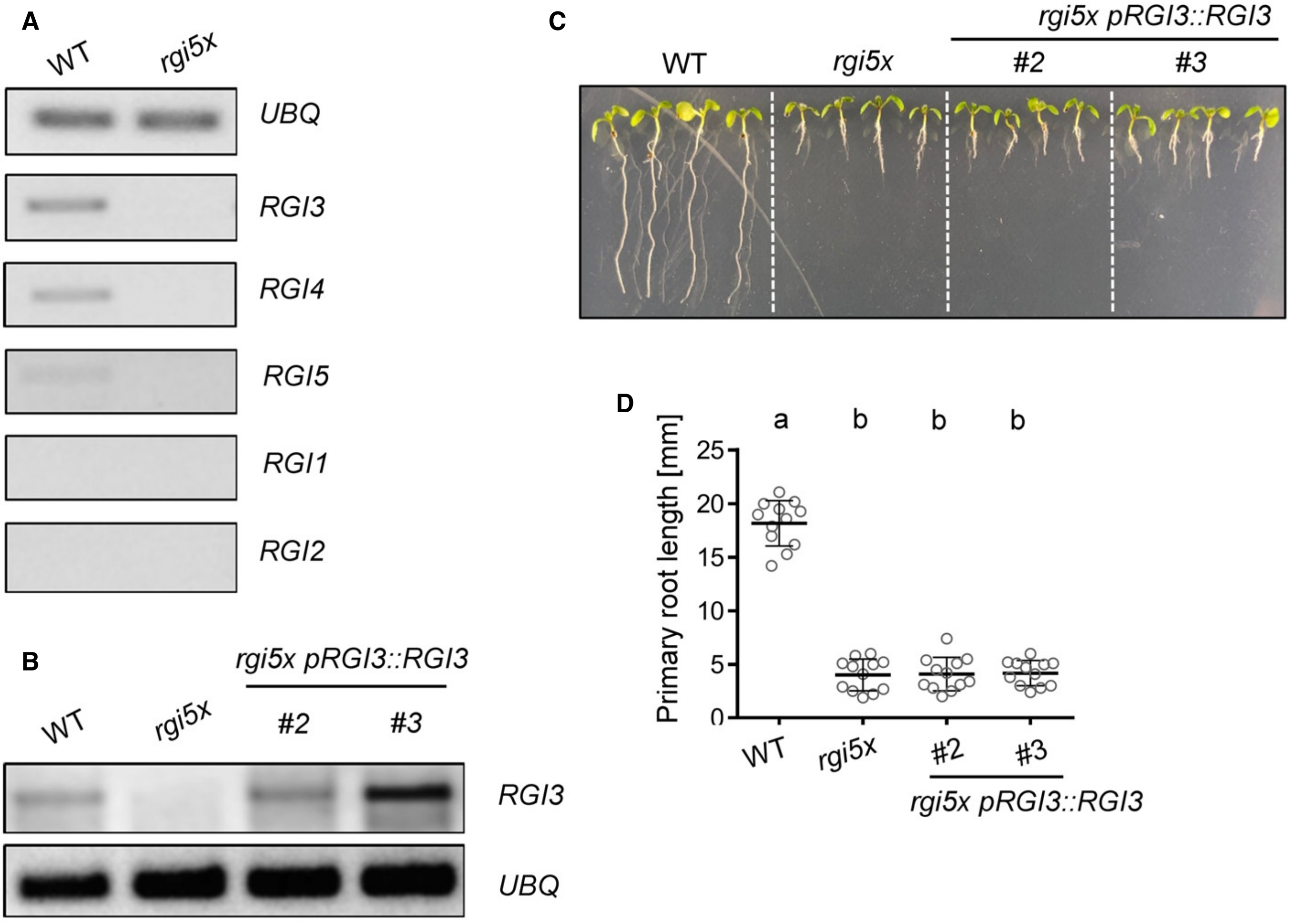

Figure EV3. Characterization of *RGI* expression in mature leaves, *RGI3* expression in and phenotypic characterization of *rgi5x pRGI3::RGI3*.

- A Semi-quantitative reverse transcription PCR showing expression of *RGI1-RGI5* in 6-week-old leaves of WT and *rgi5x*. *UBQ* was used as a control. *RGI3* and *RGI4* show comparable expression levels. *RGI5* shows weaker transcript levels and *RGI1* and *RGI2* expression was not detectable in mature leaves.
- B Semi-quantitative reverse transcription PCR showing expression of *RGI3* in transgenic *rgi5x pRGI3::RGI3* lines. *UBQ* was used as a control. Both lines of *rgi5x pRGI3::RGI3* show *RGI3* expression in mature leaves.
- C Representative pictures taken 7 days after vertical germination of the indicated genotypes on ½ MS Agar plates.
- D Quantification of main root length of seedlings shown in C. Shown is the mean of $n = 12$ biological replicates ± SD (one-way ANOVA, Tukey *post hoc* test, a-b $P < 0.001$). Expression of *pRGI3::RGI3* does not complement the short root phenotype of the *rgi5x* mutant.

Data information: Similar results were obtained in three independent experiments.

Source data are available online for this figure.

 © 2022 The Authors

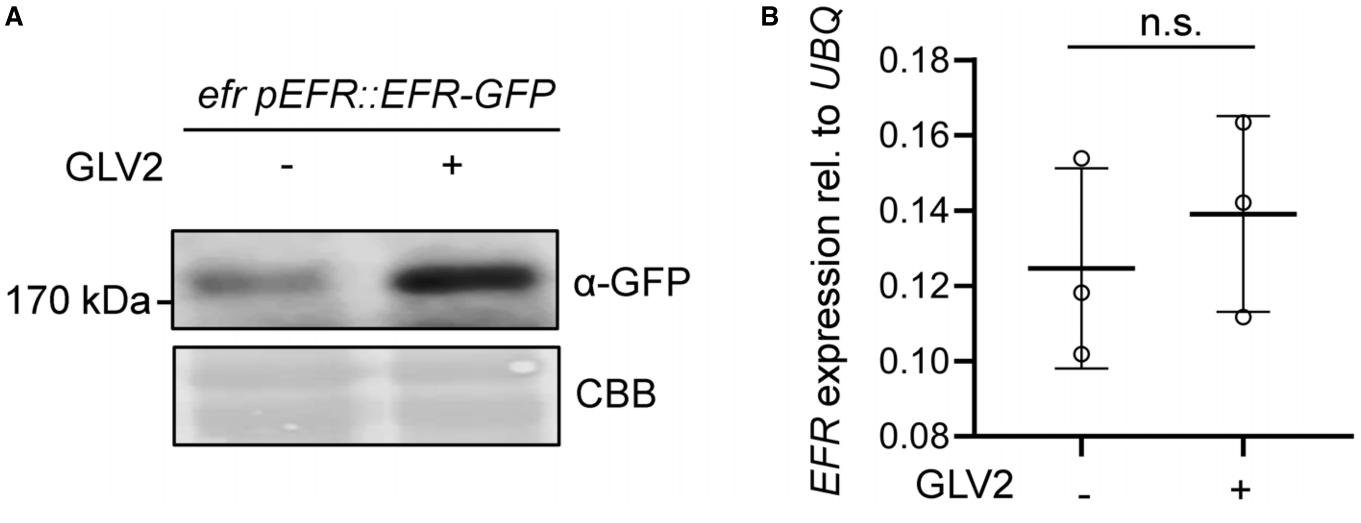

Figure EV4. GLV2 treatment promotes EFR abundance.

A Twelve-day-old *efr* pEFR::EFR-GFP seedlings were treated with mock or 1 μM GLV2 for 24 h before protein extraction. Western blots were probed with α-GFP antibodies. CBB: Coomassie brilliant blue.

B Quantitative real-time PCR of *EFR* transcripts from 12-day-old *efr* pEFR::EFR-GFP seedlings treated with mock or 1 μM GLV2 for 24 h, $n = 3$ biological replicates ± SD. *UBQ* was used as a house keeping gene. Student's *t*-test revealed no statistical difference.

Data information: Similar results were obtained in three independent experiments.

Source data are available online for this figure.

© 2022 The Authors
