# Supplementary material for: RGI‐GOLVEN signaling promotes cell surface immune receptor abundance to regulate plant immunity
Source: EMBO Rep. 2022 Mar 1;23(5):e53281. doi: 10.15252/embr.202153281 (PMC9066070; doi:10.15252/embr.202153281)

Source data Figure EV 3 A

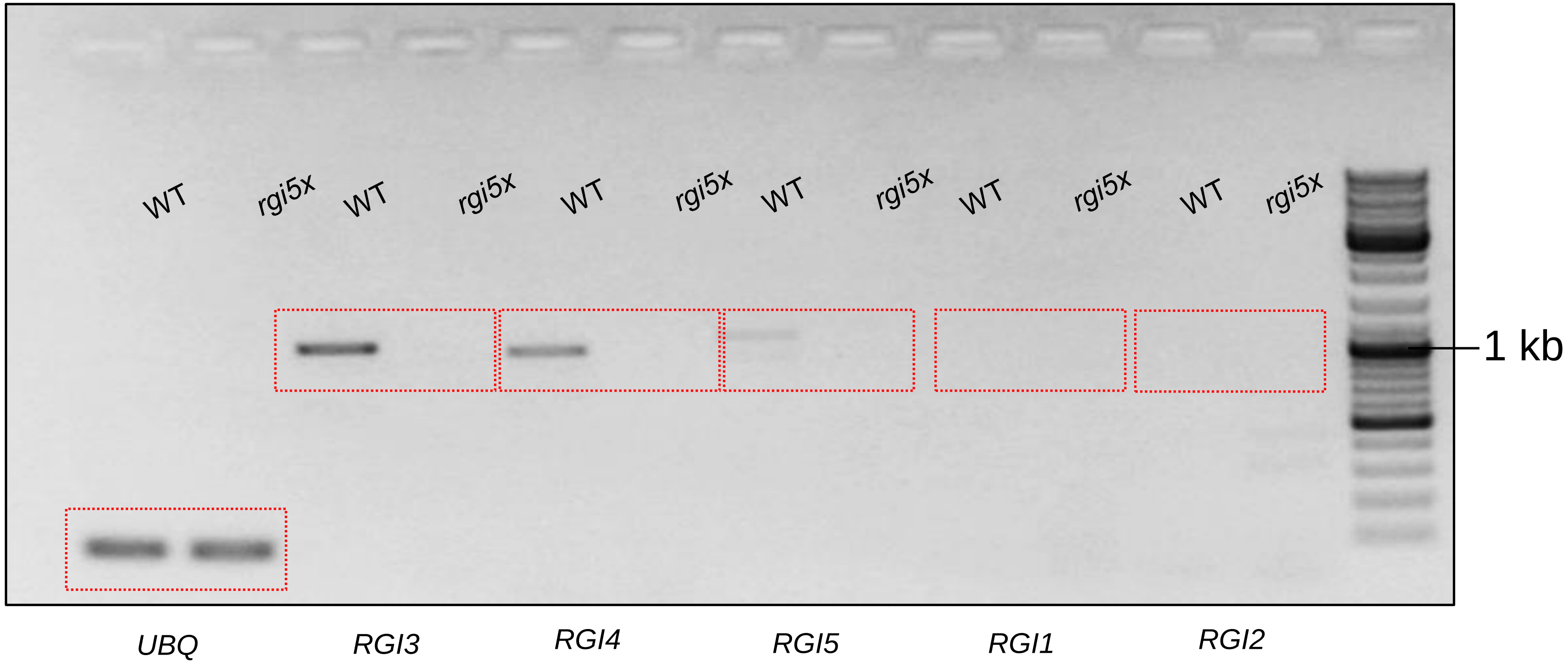

Red boxes indicate bands
used for figure assembly

Source data Figure EV 3 B

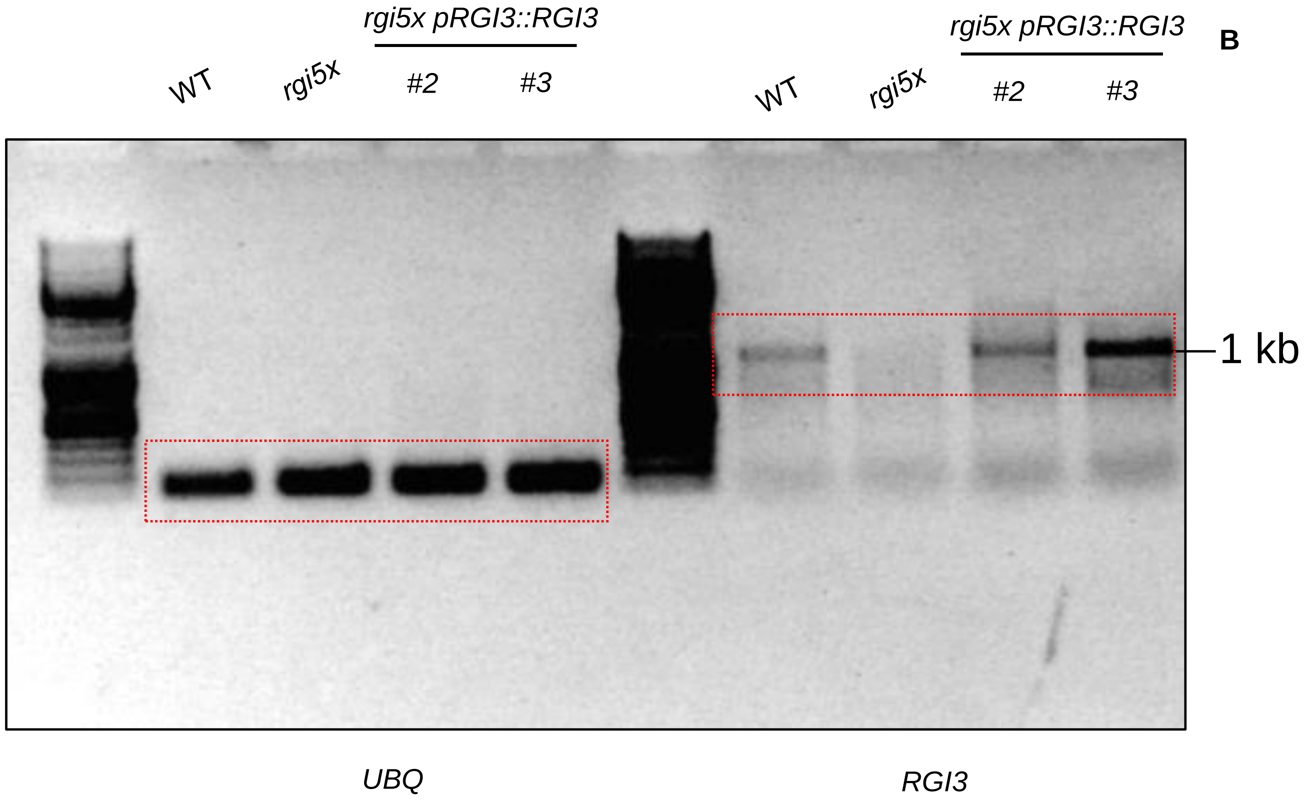

Red boxes indicate bands
used for figure assembly

Source data Figure EV 3 C

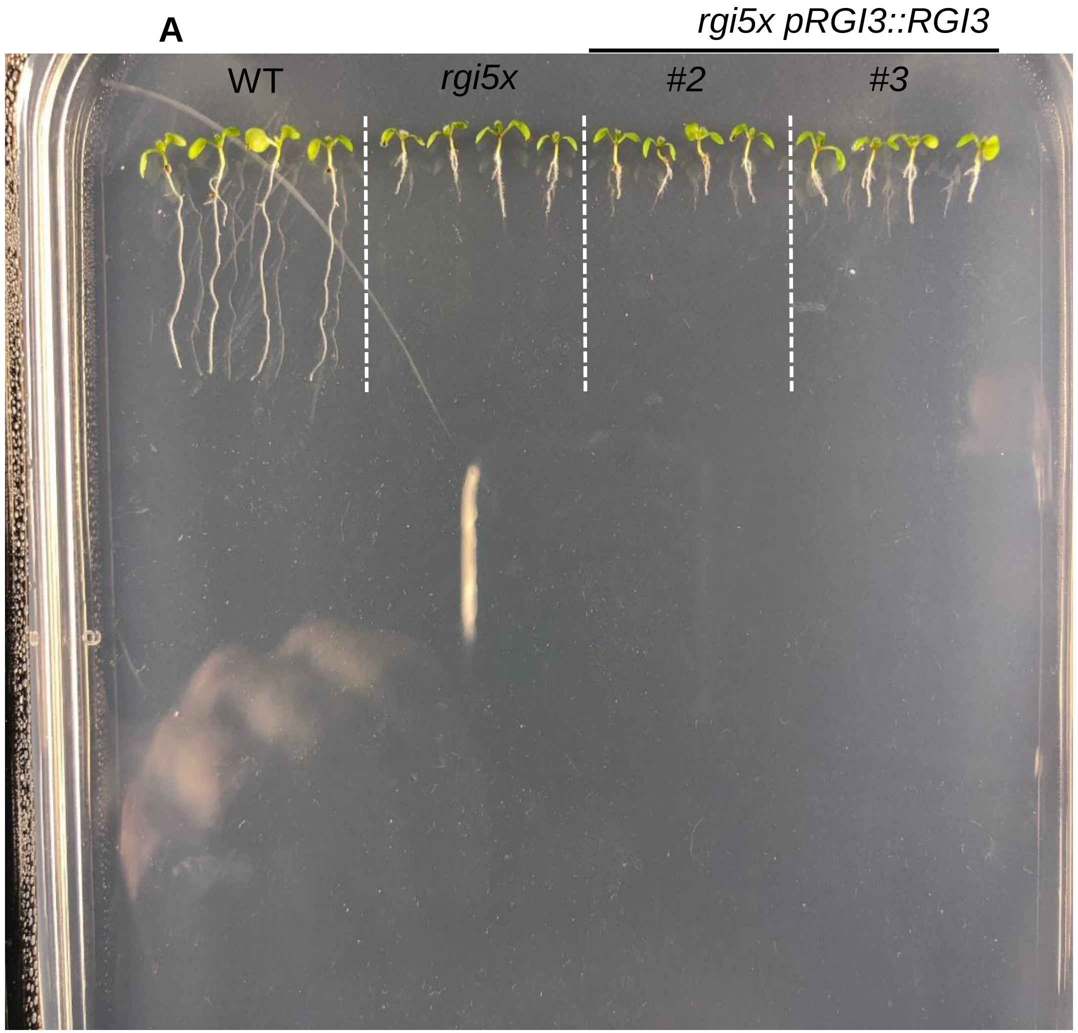

Supplement: Supplementary file 3 — Source Data for Expanded View [file EMBR-23-e53281-s002.zip › EV_Figure_Source_Data/EMBOR-2021-53281V4-Figure_Source_Data_EV3-sd.pdf]
