# Supplementary material for: RGI‐GOLVEN signaling promotes cell surface immune receptor abundance to regulate plant immunity
Source: EMBO Rep. 2022 Mar 1;23(5):e53281. doi: 10.15252/embr.202153281 (PMC9066070; doi:10.15252/embr.202153281)
Supplement: Supplementary file 7 — Source Data for Figure 4 [file EMBR-23-e53281-s009.zip › Figure_4_Source_Data/EMBOR-2021-53281V4-Figure_Source_Data_4-sd.pdf]

Source data Figure 4E

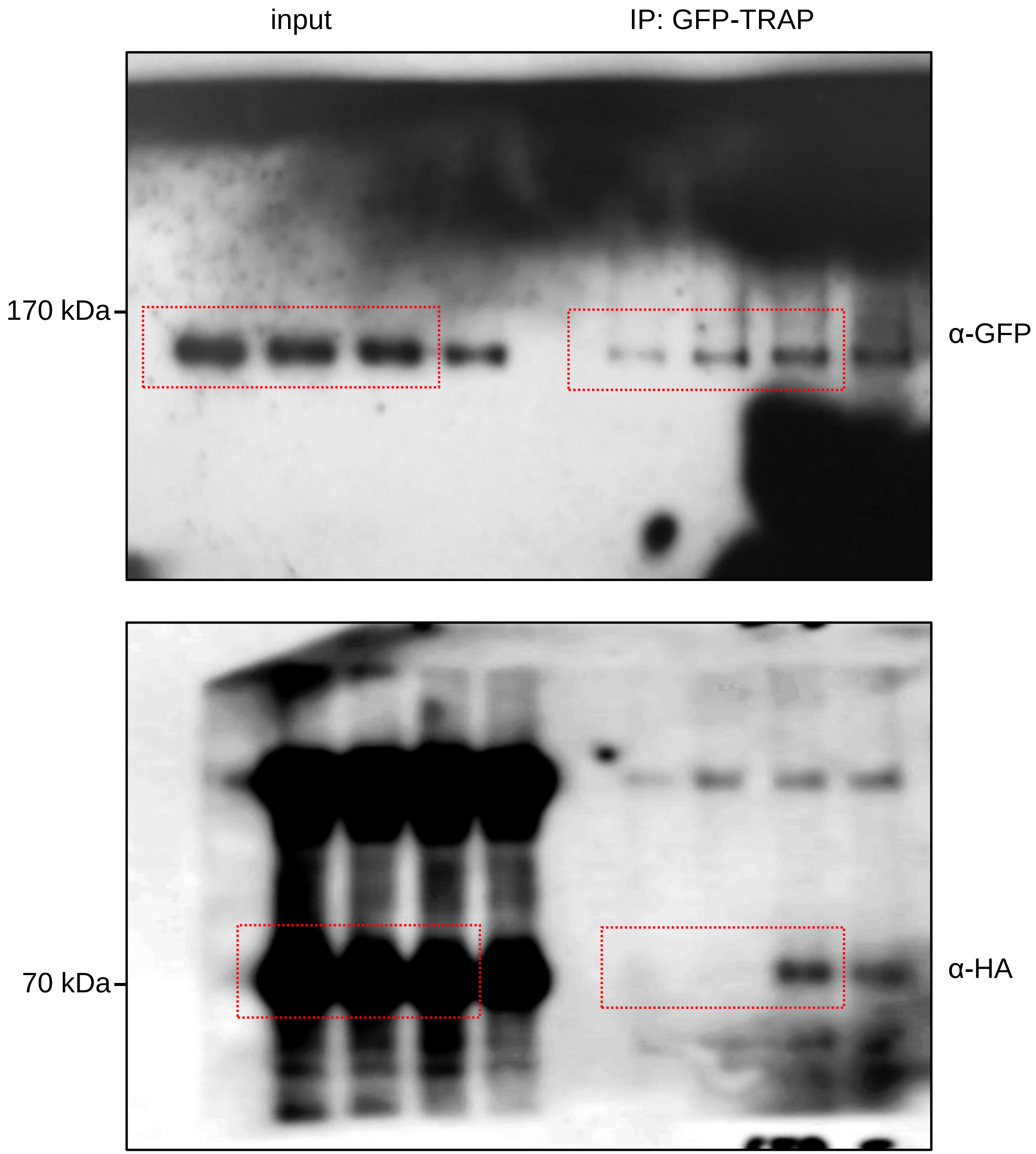

Red boxes indicate bands used for figure assembly
