# Supplementary material for: RGI‐GOLVEN signaling promotes cell surface immune receptor abundance to regulate plant immunity
Source: EMBO Rep. 2022 Mar 1;23(5):e53281. doi: 10.15252/embr.202153281 (PMC9066070; doi:10.15252/embr.202153281)
Supplement: Supplementary file 8 — Source Data for Figure 5 [file EMBR-23-e53281-s007.pdf]

Source data Figure 5A

170 kDa

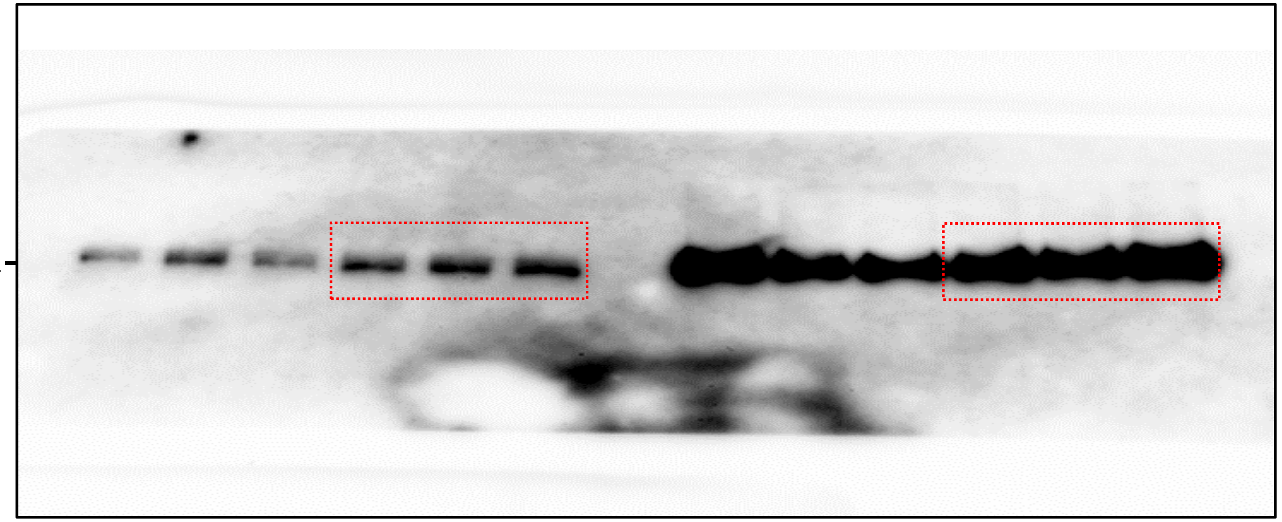

α-GFP
input

α-GFP
IP: GFP-TRAP

70 kDa

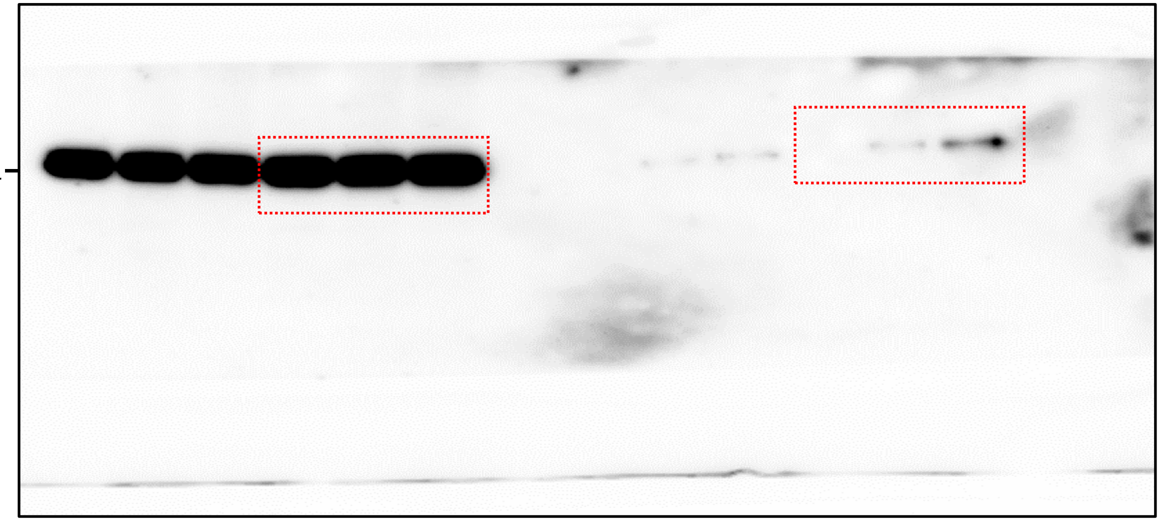

α-BAK1
input

α-BAK1
IP: GFP-TRAP

Red boxes indicate bands used for figure assembly

Source data Figure 5B

α-GFP IP: GFP-TRAP α-GFP input

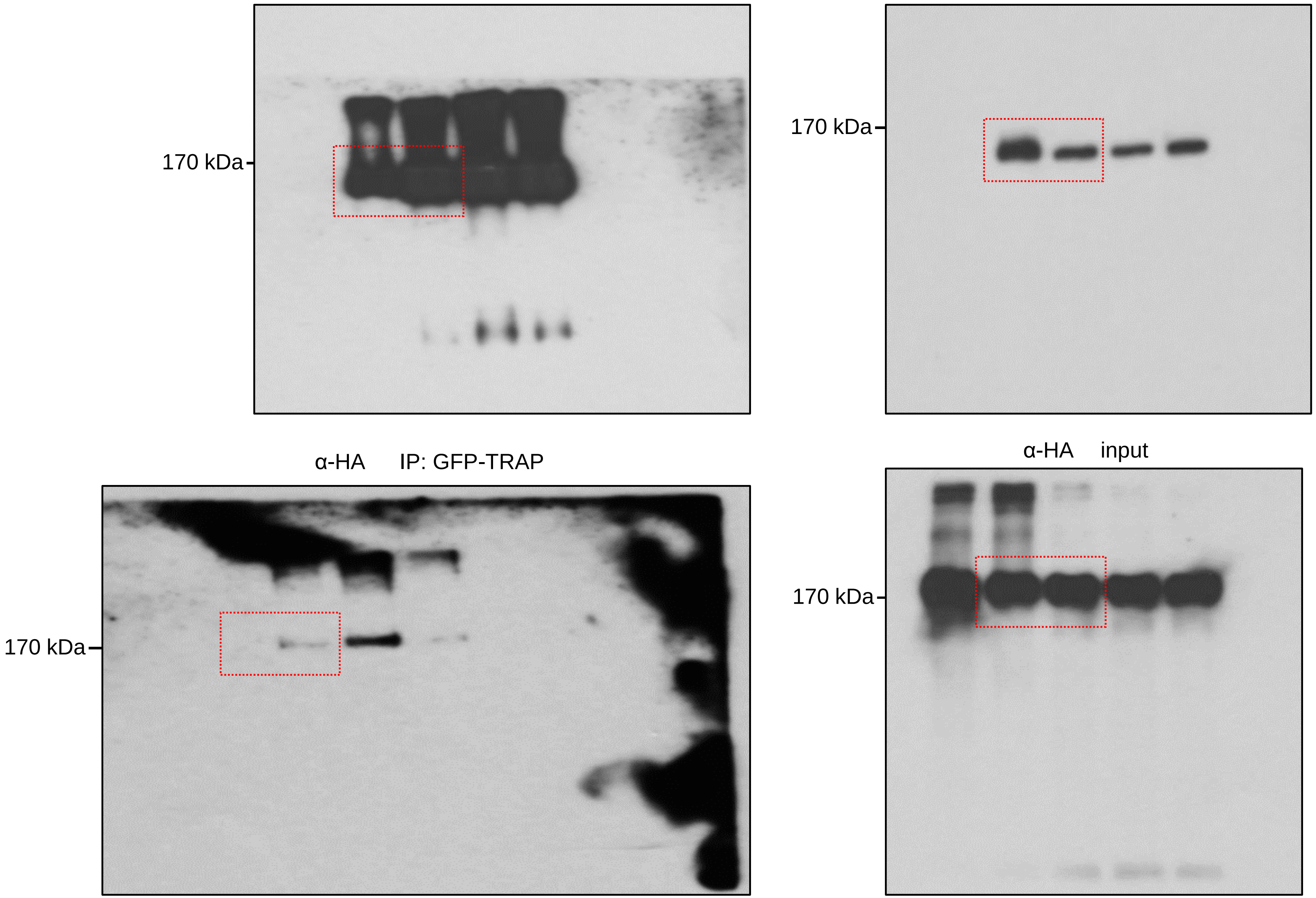

Red boxes indicate bands used for figure assembly

Source data Figure 5C

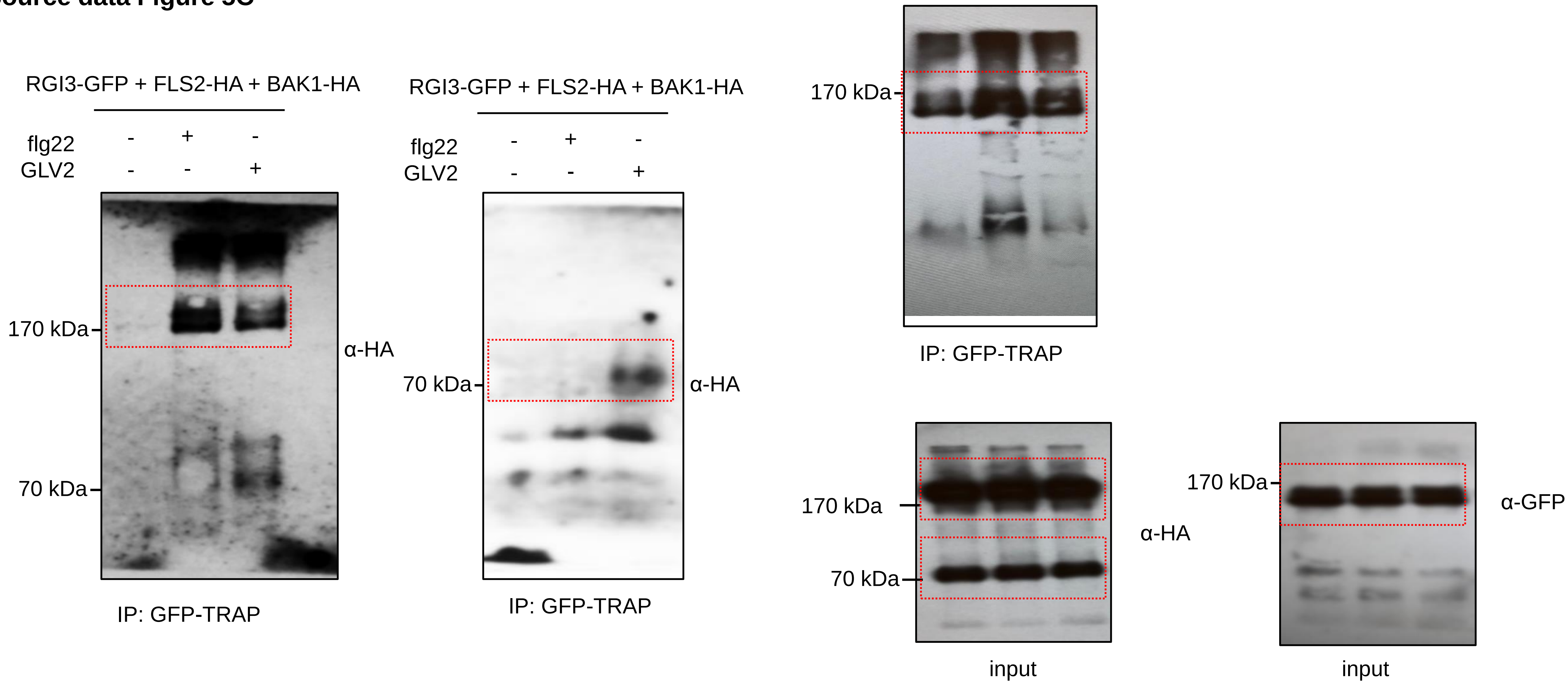

Red boxes indicate bands
used for figure assembly
