# Supplementary material for: RGI‐GOLVEN signaling promotes cell surface immune receptor abundance to regulate plant immunity
Source: EMBO Rep. 2022 Mar 1;23(5):e53281. doi: 10.15252/embr.202153281 (PMC9066070; doi:10.15252/embr.202153281)
Supplement: Supplementary file 9 — Source Data for Figure 6 [file EMBR-23-e53281-s010.zip › Figure_6_Source_Data/EMBOR-2021-53281V4-Figure_Source_Data_6-sd.pdf]

Source data Figure 6A

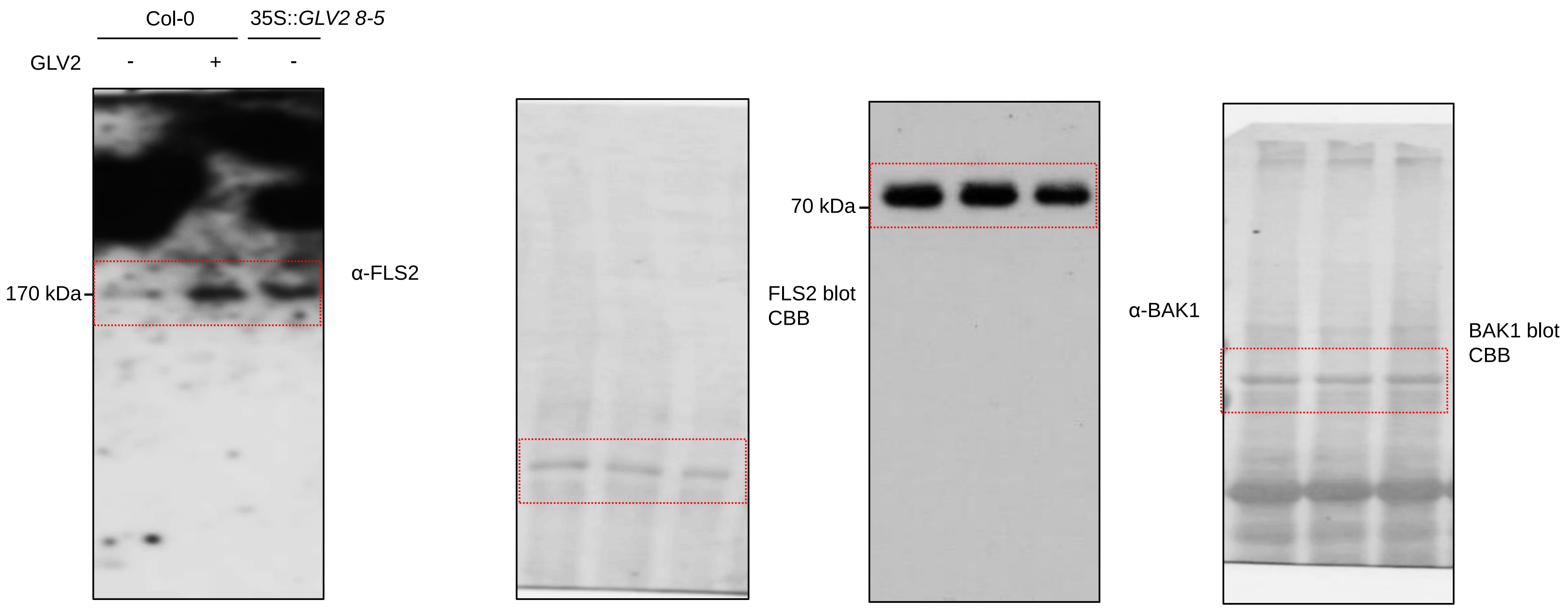

Red boxes indicate bands used for figure assembly

Source data Figure 6C

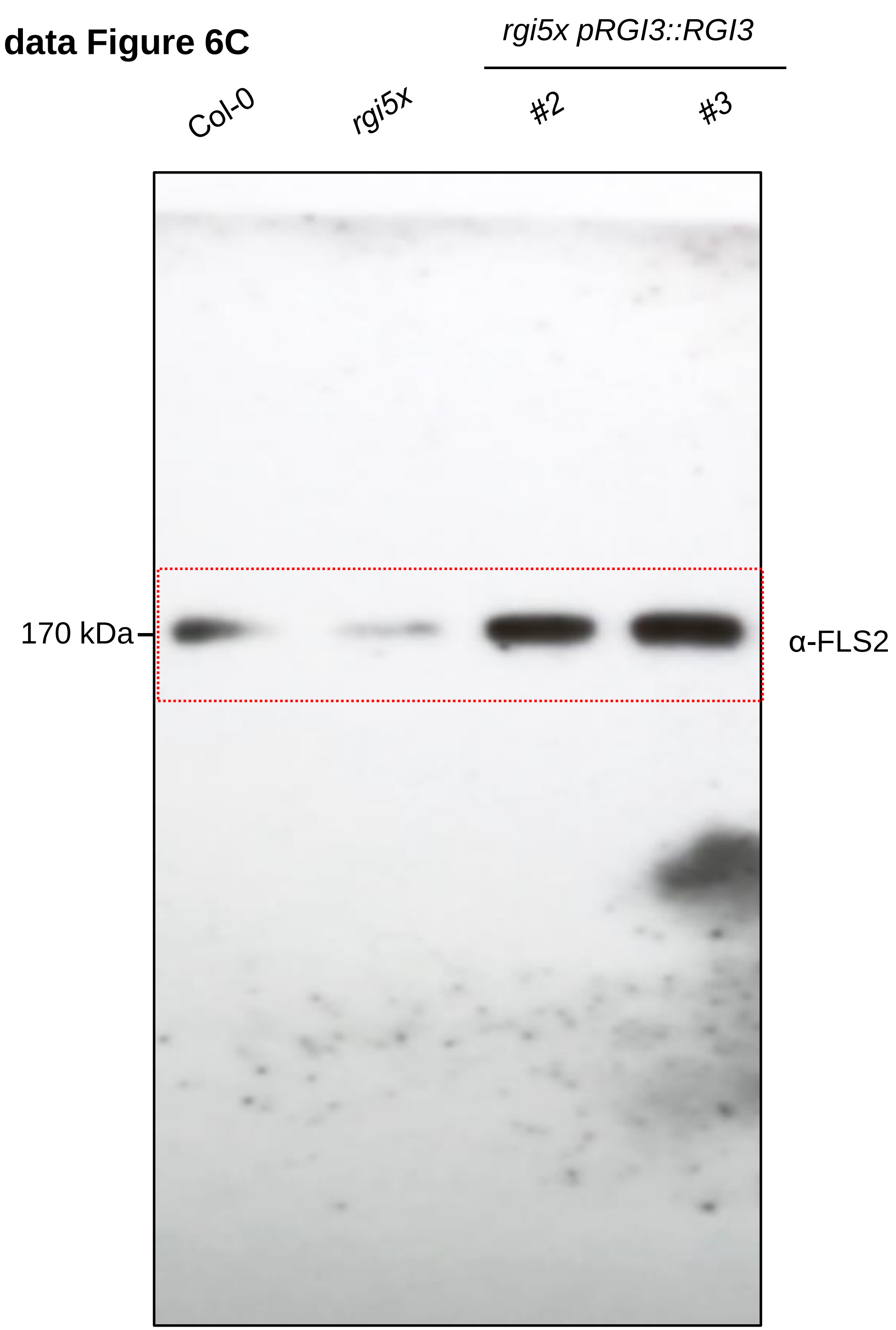

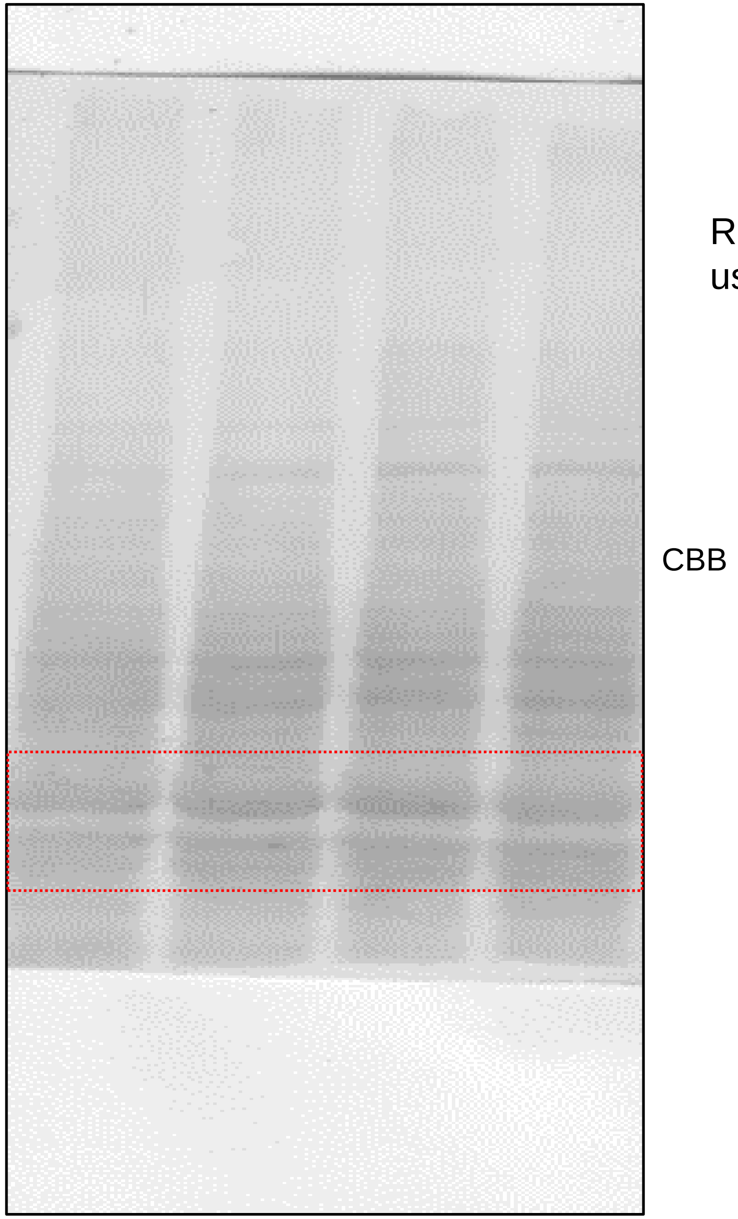

Red boxes indicate bands used for figure assembly

Source data Figure 6D

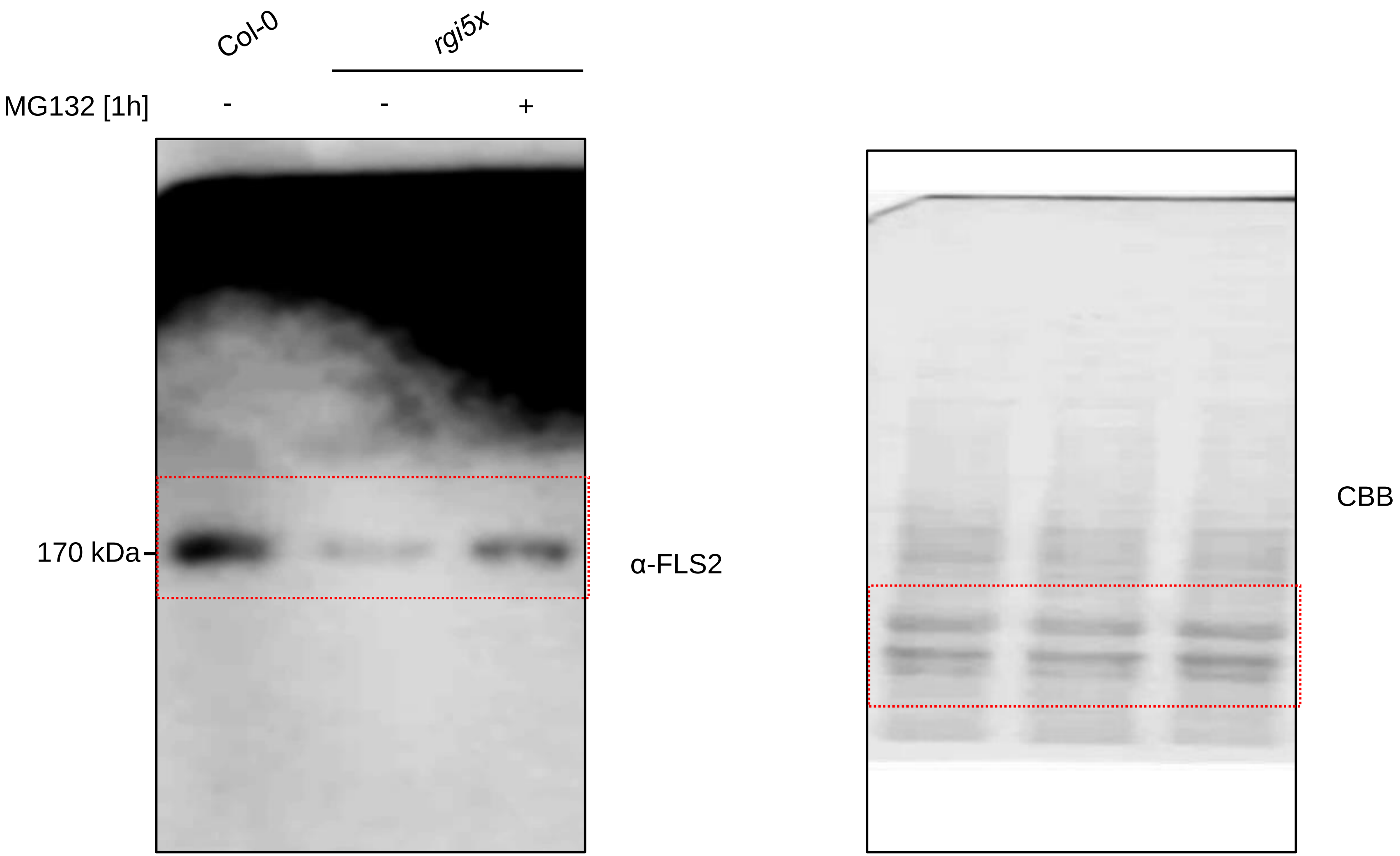

Red boxes indicate bands used for figure assembly
