# Supplementary material for: RGI‐GOLVEN signaling promotes cell surface immune receptor abundance to regulate plant immunity
Source: EMBO Rep. 2022 Mar 1;23(5):e53281. doi: 10.15252/embr.202153281 (PMC9066070; doi:10.15252/embr.202153281)
Supplement: Supplementary file 10 [file EMBR-23-e53281-s008.pdf]

EMBO reports

RGI-GOLVEN signaling promotes cell surface immune receptor abundance to regulate plant immunity

Martin Stegmann, Patricia Zecua-Ramirez, Christina Ludwig, Ho-Seok Lee, Brenda Peterson, Zachary Nimchuk, Youssef Belkhadir, and Ralph Hückelhoven
DOI: 10.15252/embr.202153281

Corresponding author(s): Martin Stegmann (martin.stegmann@tum.de)

Review Timeline:

Submission Date:	17th May 21
Editorial Decision:	25th May 21
Revision Received:	12th Oct 21
Editorial Decision:	11th Nov 21
Revision Received:	18th Jan 22
Editorial Decision:	2nd Feb 22
Revision Received:	4th Feb 22
Accepted:	7th Feb 22

Editor: Achim Breiling

Transaction Report: This manuscript was transferred to EMBO reports following peer review at The EMBO Journal.

(Note: With the exception of the correction of typographical or spelling errors that could be a source of ambiguity, letters and reports are not edited. Depending on transfer agreements, referee reports obtained elsewhere may or may not be included in this compilation. Referee reports are anonymous unless the Referee chooses to sign their reports.)

1st Editorial Decision 25th May 2021

Dear Dr. Stegmann,

Thank you for transferring your manuscript to EMBO reports. I now went through your manuscript, your p-b-p-response (revision plan) and the referee reports from The EMBO Journal (attached again below). The referees acknowledge that the findings are of interest. Nevertheless, they have raised a number of concerns and suggestions to improve the manuscript, or to strengthen the data and the conclusions drawn.

EMBO reports emphasizes novel functional over detailed mechanistic insight, but asks for clear physiological relevance of the findings, and strong experimental support of the major conclusions. Thus, we will not require addressing points regarding more mechanistic details experimentally. However, it will be necessary that in a revised manuscript you address all points questioning the main conclusions of the study, and all technical concerns, or points regarding the experimental design, model systems used, or data presentation. It would also be important to address the concerns regarding novelty (referee #1).

Given the constructive referee comments, we would like to invite you to revise your manuscript with the understanding that all referee concerns must be addressed in the revised manuscript and/or in a detailed point-by-point response. Acceptance of your manuscript will depend on a positive outcome of a second round of review. It is EMBO reports policy to allow a single round of revision only and acceptance of the manuscript will therefore depend on the completeness of your responses included in the next, final version of the manuscript.

Revised manuscripts should be submitted within three months of a request for revision. We are aware that many laboratories cannot function at full efficiency during the current COVID-19/SARS-CoV-2 pandemic and we have therefore extended our 'scooping protection policy' to cover the period required for full revision. Please contact me to discuss the revision should you need additional time, and also if you see a paper with related content published elsewhere.

When submitting your revised manuscript, please also carefully review the instructions that follow below.

PLEASE NOTE THAT upon resubmission revised manuscripts are subjected to an initial quality control prior to exposition to re-review. Upon failure in the initial quality control, the manuscripts are sent back to the authors, which may lead to delays. Frequent reasons for such a failure are the lack of the data availability section (please see below) and the presence of statistics based on n=2 (the authors are then asked to present scatter plots or provide more data points).

When submitting your revised manuscript, we will require:

1) a .docx formatted version of the final manuscript text (including legends for main figures, EV figures and tables), but without the figures included. Please make sure that changes are highlighted to be clearly visible. Figure legends should be compiled at the end of the manuscript text.

2) individual production quality figure files as .eps, .tif, .jpg (one file per figure), of main figures and EV figures. Please upload these as separate, individual files upon re-submission.

The Expanded View format, which will be displayed in the main HTML of the paper in a collapsible format, has replaced the Supplementary information. You can submit up to 5 images as Expanded View. Please follow the nomenclature Figure EV1, Figure EV2 etc. The figure legend for these should be included in the main manuscript document file in a section called Expanded View Figure Legends after the main Figure Legends section. Additional Supplementary material should be supplied as a single pdf file labeled Appendix. The Appendix should have page numbers and needs to include a table of content on the first page (with page numbers) and legends for all content. Please follow the nomenclature Appendix Figure Sx, Appendix Table Sx etc. throughout the text, and also label the figures and tables according to this nomenclature.

For more details please refer to our guide to authors:
http://www.embopress.org/page/journal/14693178/authorguide#manuscriptpreparation

See also our guide for figure preparation:
http://wol-prod-cdn.literatumonline.com/pb-assets/embo-site/EMBOPress_Figure_Guidelines_061115-1561436025777.pdf

3) a .docx formatted letter INCLUDING the reviewers' reports and your detailed point-by-point responses to their comments. As part of the EMBO Press transparent editorial process, the point-by-point response is part of the Review Process File (RPF), which will be published alongside your paper.

4) a complete author checklist, which you can download from our author guidelines (https://www.embopress.org/page/journal/14693178/authorguide). Please insert page numbers in the checklist to indicate where the requested information can be found in the manuscript. The completed author checklist will also be part of the RPF.

Please also follow our guidelines for the use of living organisms, and the respective reporting guidelines:
http://www.embopress.org/page/journal/14693178/authorguide#livingorganisms

5) that primary datasets produced in this study (e.g. RNA-seq, ChIP-seq and array data) are deposited in an appropriate public database. This is now mandatory (like the COI statement). If no primary datasets have been deposited in any database, please state this in this section (e.g. 'No primary datasets have been generated and deposited').

See also: http://embor.embopress.org/authorguide#datadeposition

Please remember to provide a reviewer password if the datasets are not yet public.

The accession numbers and database should be listed in a formal "Data Availability " section (placed after Materials & Methods) that follows the model below. Please note that the Data Availability Section is restricted to new primary data that are part of this study.

Data availability

The datasets produced in this study are available in the following databases:

- RNA-Seq data: Gene Expression Omnibus GSE46843 (https://www.ncbi.nlm.nih.gov/geo/query/acc.cgi?acc=GSE46843)
- [data type]: [name of the resource] [accession number/identifier/doi] ([URL or identifiers.org/DATABASE:ACCESSION])

*** Note - All links should resolve to a page where the data can be accessed. ***

Moreover, I have these editorial requests:

6) We strongly encourage the publication of original source data with the aim of making primary data more accessible and transparent to the reader. The source data will be published in a separate source data file online along with the accepted manuscript and will be linked to the relevant figure. If you would like to use this opportunity, please submit the source data (for example scans of entire gels or blots, data points of graphs in an excel sheet, additional images, etc.) of your key experiments together with the revised manuscript. If you want to provide source data, please include size markers for scans of entire gels, label the scans with figure and panel number, and send one PDF file per figure.

7) Our journal encourages inclusion of *data citations in the reference list* to directly cite datasets that were re-used and obtained from public databases. Data citations in the article text are distinct from normal bibliographical citations and should directly link to the database records from which the data can be accessed. In the main text, data citations are formatted as follows: "Data ref: Smith et al, 2001" or "Data ref: NCBI Sequence Read Archive PRJNA342805, 2017". In the Reference list, data citations must be labeled with "[DATASET]". A data reference must provide the database name, accession number/identifiers and a resolvable link to the landing page from which the data can be accessed at the end of the reference. Further instructions are available at: http://www.embopress.org/page/journal/14693178/authorguide#referencesformat

8) Regarding data quantification and statistics, please specify, where applicable, the number "n" for how many independent experiments (biological or technical replicates) were performed, the bars and error bars (e.g. SEM, SD) and the test used to calculate p-values in the respective figure legends. Please provide statistical testing where applicable, and also add a paragraph detailing this to the methods section. See:
http://www.embopress.org/page/journal/14693178/authorguide#statisticalanalysis

9) Please note our new reference format:
http://www.embopress.org/page/journal/14693178/authorguide#referencesformat

10) Please add up to five key words to the title page and order the manuscript sections like this:
Title page - Abstract - Introduction - Results - Discussion - Materials and Methods -Data availability section - Acknowledgements - Author contributions - Conflict of interest statement - References - Figure legends - Expanded View Figure legends.

I look forward to seeing a revised version of your manuscript when it is ready. Please let me know if you have questions or comments regarding the revision.

Kind regards,

Achim

Achim Breiling
Editor

EMBO Reports

Referee #1:

In the manuscript entitled "RGI-GOLVEN signaling promotes FLS2 abundance to regulate plant immunity", the authors provide genetic evidence that an endogenous GLV2 peptide sensitizes immune signaling through the flagellin receptor FLS2 in Arabidopsis thaliana. They further show that, following ligand perception, GLV2 receptor RGI3 physically associates with FLS2 and increases FLS2 protein accumulation. However, the mechanisms by which GLV2-RGI3 leads to an increase in FLS2 abundance and the extent to which this regulation applies to different PRRs remain to be addressed. As recent PTI-ETI studies in plants reported in Nature remind that PRR abundance control provides a critical step in plant immunity, this work adds new insight into this interesting topic and has potential to appeal to the readership of the journal. However, in light of previous studies (Wang et al New Phytologist 2021, reporting RGF7 peptide-RGI4/RGI5 involving BAK1/SERK4 to enhance plant immunity), conceptual advance seems not strong at least in the present form of the manuscript. Detailed comments are below.

The title is not kind to the readership beyond the area of plant immunity or peptide signaling.

To relate this study to previous studies on this peptide family, the authors should show which GLV and RGF peptides correspond to each other, together with receptors, in a table.

Lines 59-60, the data suggest that PROPEP processing is not only through METACASPASE4.
Lines 63-64, recent studies on SCOOP-MIK2 need to be cited.

Line 113-114, Wang et al 2021 reference information is incomplete.

In Fig S2, GLV1/2 mRNA levels are reduced in response to bacterial challenge, but not altered in response to flg22. Peptide expression and release need to be tested in response to bacterial challenge.

Line 155, what is COR and why cor- strain was used need to be explained.

In Fig 4, dose dependence and Kd values should be determined and compared with previously described peptide-receptors.

Lines 229-230, it reads abrupt to test possible RGI3-FLS2 interaction here. It helps if the authors cite previous studies on RGI-BAK1 interactions to provide a rationale.
Do RGI3-FLS2-BAK1 reside in the same complex and how does RGI3 regulates FLS2? Further insight is expected to be obtained by some more feasible experiments. For instance, in my view it is important and feasible to test how GLV2-RGI3 influences FLS2-BAK1 association in vitro and/or in planta.

In terms of FLS2 abundance regulation, possible proteasome dependence can be easily tested by using proteasome inhibitors. It is also important to test whether this applies to different BAK1-dependent LRR-RKs or PRRs. Even if these investigations are beyond the scope of this work, previous studies on PRR abundance control, including ERQC and internalization of the receptors, need to be cited and discussed.

Fig 6C indicates a dramatic decrease in the steady-state FLS2 levels in rgi5x, but the observed defects of flg22 responses in the mutant seems quite mild in Fig 3. How could the authors explain this? In Fig 3, fls2 mutant control is needed.

Referee #2:

Plant endogenous peptide-receptor signaling has been implicated to play a central role in plant growth, development, and responses to environmental stresses. In this manuscript, the authors presented data and showed that GLV2 peptides perceived by the RGI3 receptor kinase are involved in bacterial flagellin-mediated immunity and disease resistance against bacterial infections. In addition, RGI3 complexes with FLS2 and is required to maintain the FLS2 protein abundance. Although the other pairs of GLV and RGI have been identified in mediating diverse biological processes, including immunity, this work provides the first evidence showing RGI3 as the receptor of GLV2 in plant immunity. The data also suggested that GLV2-RGI3 regulates FLS2 protein abundance despite an unclear mode of action. Overall, this is a piece of interesting work and adds new players in plant immunity.

The authors have shown nicely that RGI3 is genetically required for GLV2-mediated responses. In addition, the extracellular domain of RGI3 binds to GLV2. In this assay (Fig 4D), the authors used flg22 as a negative control. However, GLV2-S, which lost the immunomodulatory function (Fig S5), should be included as a control for binding assay (Fig 4D). The part on that GLV2-RGI3 associates with and affects the FLS2 abundance was weakly elucidated (Figure 5, co-IP in N. benthamiana). Considering that this is a critical point to indicate how GLV2-RGI3 functions in plant immunity, to strengthen this claim, I would suggest the authors perform co-IP assays in Arabidopsis RGI3 or GLV2 transgenic plants using FLS2 antibodies. The authors have these

resources and should be able to do this in a reasonable time frame. Such data will strengthen their claims. The authors may discuss why and how GLV treatment increases the FLS2 abundance.

Minor points:
1. A comparison of the expression levels of all GLVs in different tissues would be helpful for the readers to understand why in some cases, some GLVs were selected for specific purposes, given that GLVs exhibit different expression patterns.

2. Line 79, for SCOOPs, two more most recently published papers should also be cited (Rhodes et al. 2021. Nat. Commun.; Hou et al., 2021. bioRxiv "Immune elicitation by sensing the conserved signature from phytocytokines and microbes via the Arabidopsis MIK2 receptor")

3. Figure S5, a loading control is missing for Panel (A).

Response to referee comments:

Referee #1:

In the manuscript entitled "RGI-GOLVEN signaling promotes FLS2 abundance to regulate plant immunity", the authors provide genetic evidence that an endogenous GLV2 peptide sensitizes immune signaling through the flagellin receptor FLS2 in Arabidopsis thaliana. They further show that, following ligand perception, GLV2 receptor RGI3 physically associates with FLS2 and increases FLS2 protein accumulation. However, the mechanisms by which GLV2-RGI3 leads to an increase in FLS2 abundance and the extent to which this regulation applies to different PRRs remain to be addressed. As recent PTI-ETI studies in plants reported in Nature remind that PRR abundance control provides a critical step in plant immunity, this work adds new insight into this interesting topic and has potential to appeal to the readership of the journal. However, in light of previous studies (Wang et al New Phytologist 2021, reporting RGF7 peptide-RGI4/RGI5 involving BAK1/SERK4 to enhance plant immunity), conceptual advance seems not strong at least in the present form of the manuscript.

RESPONSE:

Thank you for the overall positive assessment of our work. However, we disagree that the presented work`s conceptual advance is not strong enough in light of the recent Wang et al., New Phytologist 2021 manuscript. Our work provides significant progress in the understanding of GLV peptides and their receptors in the control of plant immunity:

- Our paper describes the importance of additional GLV peptides for plant immunity and that this is not limited to the pathogen/flg22-inducibly expressed GLV4/RGF7.
- Our paper reports that GLV peptides do no only function as danger signals (as reported for RGF7/GLV4) but also sensitize the plant`s responsiveness to the important and well-studied bacterial elicitor flg22. This opens a whole avenue for future research as it shows that GLV signalling and FLS2-triggered immunity are intimately interconnected. Furthermore, new data obtained during revision shows that the GLV-RGI pathway also controls elf18-induced responses and is not restricted to the control of FLS2-mediated immunity, suggesting that the pathway represents a general module controlling cell surface immunity.
- RGF7/GLV4 peptide application does not mimic preRGF7/GLV4 inducible overexpression in the Wang et al. paper. Thus, it remains unknown whether the reported effects for RGF7/GLV4-induced immune responses are direct or indirect. By contrast, our work reveals that direct application of GLV2 influences immune signalling by FLS2. Furthermore, we show that mature GLV2 peptides (as previously shown for roots) also accumulate in the apoplastic space of leaves where they can be perceived by RGI3 to modulate above-ground immune signalling.
- Our work reveals a novel role for RGI3 in GLV2 signalling during PTI. Wang and colleagues report the identification of RGI4 and RGI5 as leaf-expressed RGF7/GLV4 receptors during immunity. By contrast, we show that RGI3 is actually the strongest expressed RGI in mature leaves, binds GLV2 and is part of PRR signalling platforms. RGI3 is dynamically recruited to the FLS2 receptor complex dependent on flg22 and GLV2 perception. This provides first mechanistic insight in the cross-talk between the GLV-RGI signalling pathway and FLS2-triggered immunity. Also, it is the first report of a growth-regulatory receptor kinase being recruited to a ligand-activated PRR receptor complex. In addition, new data obtained during revision suggests that GLV2 perception can promote FLS2-BAK1 complex formation, the first report of a phytocytokine with such a function.
- We suggest a mechanistic basis of signalling cross-talk between GLV2-RGI3 signalling and FLS2 by showing that GLV2 perception through RGIs controls posttranscriptional FLS2 abundance and consequently signalling. Furthermore, new data obtained during revision suggests that ubiquitination-dependent degradation of FLS2 is promoted in *rgi5x*. This is the first report suggesting such a mechanism underlying phytocytokine activity. This opens interesting future research directions to understand the molecular basis.

Overall, we are convinced that the current manuscript presents a clear conceptual advance on our understanding of the function of GLV peptides in controlling plant immune signalling and nicely complements the previous findings by Wang and colleagues

Detailed comments are below.

The title is not kind to the readership beyond the area of plant immunity or peptide signaling.

RESPONSE:

The title of the manuscript has been changed to: "RGI-GOLVEN signaling promotes cell surface immune receptor abundance to regulate plant immunity" which should be more appealing to a wider audience

To relate this study to previous studies on this peptide family, the authors should show which GLV and RGF peptides correspond to each other, together with receptors, in a table.

RESPONSE:

Such a table was published in a review by Fernandez et al., Journal of Experimental Botany 2013. We updated this chart by including known RGI/RGFR receptors for GLV/RGF peptides in the new Appendix Table S1. We distinguish between genetic demonstration of a ligand-receptor relationship and biochemical evidence for direct interaction.

Lines 59-60, the data suggest that PROPEP processing is not only through METACASPASE4.

RESPONSE:

The text was modified to reflect this.

Lines 63-64, recent studies on SCOOP-MIK2 need to be cited.

RESPONSE:

Citations were added.

Line 113-114, Wang et al 2021 reference information is incomplete.

RESPONSE:

The reference was corrected. Upon initial submission, the manuscript of Wang and colleagues did not yet have assigned volume, issue and page numbers, which was now updated.

In Fig S2, GLV1/2 mRNA levels are reduced in response to bacterial challenge, but not altered in response to flg22. Peptide expression and release need to be tested in response to bacterial challenge

RESPONSE:

Thanks for suggesting this experiment. We now tested GLV2 peptide abundance in apoplastic wash fluids 24 hours after infection with *Pto* (new figure EV 1C). Similar to flg22 treatment, we did not observe a significant difference in GLV2 peptide levels after infection. Thus, we removed the statement from the discussion where we hypothesized that *Pto* may actively inhibit GLV2 maturation or expression.

Line 155, what is COR and why cor- strain was used need to be explained.

RESPONSE:

The *Pto* COR- strain lacks the effector molecule coronatine, which makes it less virulent and therefore easier to assess PTI related phenotypes. This *Pto* mutant is routinely used in the literature (e.g. Stegmann et al., Science 2017).

In Fig 4, dose dependence and Kd values should be determined and compared with previously described peptide-receptors.

RESPONSE:

Thank you for suggesting this experiments which we performed. The data is now included in the manuscript as the new figure 4D. We show by MST that GLV2 binds to RGI3 with high affinity (K_D=11.82 nm +/- 10.85 nm). For specificity, we included GLV2-S as a control which shows strongly reduced binding (K_D=129.26 +/- 81.69 nm). These affinities are in line with previously reported receptor-sulfated peptide interactions.

Lines 229-230, it reads abrupt to test possible RGI3-FLS2 interaction here. It helps if the authors cite previous studies on RGI-BAK1 interactions to provide a rationale.
Do RGI3-FLS2-BAK1 reside in the same complex and how does RGI3 regulates FLS2? Further insight is expected to be obtained by some more feasible experiments. For instance, in my view it is important and feasible to test how GLV2-RGI3 influences FLS2-BAK1 association in vitro and/or in planta.

RESPONSE:

The rationale for testing FLS2-RGI3 interaction and the papers referring to previously known complex formation between RGIs and BAK1/SERKs are now better explained and referred to in the results section of the manuscript. Also, we added a new figure 4E to the MS showing GLV2-induced RGI3-BAK1 interaction which fits better to Fig. 4 as it is an additional indication for RGI3 being a bona-fide receptor for GLV2. Furthermore, we tested whether FLS2-BAK1 complex formation is affected by GLV2 peptide co-treatment with flg22. Interestingly, FLS2-BAK1 complex formation was promoted in the presence of GLV2, suggesting that RGI-GLV signaling is part of PRR platforms and can already affect the activation of the receptor complex. These data were now added to the manuscript as the new Figure 5A.

In terms of FLS2 abundance regulation, possible proteasome dependence can be easily tested by using proteasome inhibitors. It is also important to test whether this applies to different BAK1-dependent LRR-RKs or PRRs. Even if these investigations are beyond the scope of this work, previous studies on PRR abundance control, including ERQC and internalization of the receptors, need to be cited and discussed.

RESPONSE:

We performed MG132 (proteasome inhibitor) treatments to test whether the defect in FLS2 accumulation in *rgi5x* can be complemented. Indeed, we found partial restoration of FLS2 levels upon treatment which is now added to the manuscript as the new Figure 6D. Concerning the involvement of GLV2-RGI3 signalling in additional SERK-dependent PRR signalling pathways: We tested whether *rgi5x* and the CRISPR *glv* mutant are affected in elf18-induced immune responses. indeed, both mutants show reduced sensitivity to elf18 in seedling growth inhibition experiments which we added as the new Figure 3F. We do not agree that ERQC control studies need to be cited, as FLS2 signalling is not primarily regulated by ERQC.

Fig 6C indicates a dramatic decrease in the steady-state FLS2 levels in rgi5x, but the observed defects of flg22 responses in the mutant seems quite mild in Fig 3. How could the authors explain this? In Fig 3, fls2 mutant control is needed.

RESPONSE:

We do not have an explanation for this but it is possible that the residual amounts of FLS2 detectable in *rgi5x* are sufficient to confer a certain degree of flg22 sensitivity, albeit to a lower extent. As suggested, we included a *fls2* mutant control in the *Pto* COR- infection experiment depicted in Fig. 3D. *rgi5x* mutants show an enhanced susceptibility phenotype similar to *fls2* mutants but slightly weaker compared to *bak1-5 bkk1*.

Referee #2:

Plant endogenous peptide-receptor signaling has been implicated to play a central role in plant growth, development, and responses to environmental stresses. In this manuscript, the authors presented data and showed that GLV2 peptides perceived by the RGI3 receptor kinase are involved in bacterial flagellin-mediated immunity and disease resistance against bacterial infections. In addition, RGI3 complexes with FLS2 and is required to maintain the FLS2 protein abundance. Although the other pairs of GLV and RGI have been identified in mediating diverse biological processes, including immunity, this work provides the first evidence showing RGI3 as the receptor of GLV2 in plant immunity. The data also suggested that GLV2-RGI3 regulates FLS2 protein abundance despite an unclear mode of action. Overall, this is a piece of interesting work and adds new players in plant immunity.

RESPONSE:

Thank you very much for the overall positive assessment of our work.

The authors have shown nicely that RGI3 is genetically required for GLV2-mediated responses. In addition, the extracellular domain of RGI3 binds to GLV2. In this assay (Fig 4D), the authors used flg22 as a negative control. However, GLV2-S, which lost the immunomodulatory function (Fig S5), should be included as a control for binding assay (Fig 4D). The part on that GLV2-RGI3 associates with and affects the FLS2 abundance was weakly elucidated (Figure 5, co-IP in N. benthamiana). Considering that this is a critical point to indicate how GLV2-RGI3 functions in plant immunity, to strengthen this claim, I would suggest the authors perform co-IP assays in Arabidopsis RGI3 or GLV2 transgenic plants using FLS2 antibodies. The authors have these resources and should be able to do this in a reasonable time frame. Such data will strengthen their claims. The authors may discuss why and how GLV treatment increases the FLS2 abundance.

RESPONSE:

Thank you very much for your suggested experiments. We now performed MST experiments using GLV2-S as a negative control and can show that RGI3 binds with high affinity to GLV2 and a strongly reduced affinity to GLV2-S. This data has been added as the new Fig. 4D. Concerning the co-IP experiments in *Arabidopsis*: Unfortunately, the line expressing *pRGI3::RGI3* used in our manuscript (despite the fact that the construct contains a C-terminal GFP tag and complements the PTI defects of the *rgi5x* mutant) never showed detectable RGI3-GFP protein accumulation by western blot analysis. This renders the line unsuitable for *in vivo* biochemical analysis. We also tried to generate RGI3-GFP overexpression lines but also these did not show detectable protein accumulation for yet unknown reasons. However, the fact that GLV2 treatment promotes flg22-induced FLS2-BAK1 complex formation after 10 minutes of treatment suggests that RGI3 is likely also part of FLS2 complexes under native conditions in Arabidopsis (new Fig. 5A).

Minor points:

1. A comparison of the expression levels of all GLVs in different tissues would be helpful for the readers to understand why in some cases, some GLVs were selected for specific purposes, given that GLVs exhibit different expression patterns.

RESPONSE:

A new panel was added as Figure EV1B showing the predicted expression of all *GLV* genes throughout different plant tissues. This shows that GLV2 is the strongest expressed GLV peptide family member in leaf tissue. Therefore, we chose to focus our subsequent analysis on GLV2.

Line 79, for SCOOPs, two more most recently published papers should also be cited (Rhodes et al. 2021. Nat. Commun.; Hou et al., 2021. bioRxiv "Immune elicitation by sensing the conserved signature from phytocytokines and microbes via the Arabidopsis MIK2 receptor")

RESPONSE:

References were added as suggested.

3. Figure S5, a loading control is missing for Panel (A).

RESPONSE:

Figure S5 did not show a western blot or Agarose gel so we are not sure which panel the referee is referring to.

Dear Dr. Stegmann,

Thank you for the submission of your revised manuscript to our editorial offices. I have now received the reports from the three referees that were asked to re-evaluate your study, you will find below. As you will see, referees #1 now supports the publication of your study. Referee #2 has remaining concerns and suggestions to improve the manuscript, I ask you to address in a final revised version. Please also provide a point-by-point response regarding these remaining points.

Moreover, I have these editorial requests I also ask you to address:

- Please shorten the abstract to not more than 175 words.

- Please remove the paragraph 'This PDF file includes:' from the title page.

- Please make sure that the number "n" for how many independent experiments were performed, their nature (biological versus technical replicates), the bars and error bars (e.g. SEM, SD) and the test used to calculate p-values is indicated in the respective figure legends (also of the diagrams in the Appendix), and that statistical testing has been done where applicable. Please avoid phrases like 'independent experiment' or 'independent replicate', but clearly state if these were biological or technical replicates. If statistical testing was done but there is no significant difference, please also mark this in the diagrams (n.s.). It seems presently some diagrams have only partial stats.

- Finally, please find attached a word file of the manuscript text (provided by our publisher) with changes we ask you to include in your final manuscript text, and some queries, we ask you to address. Please provide your final manuscript file with track changes, in order that we can see any modifications done.

In addition, I would need from you:
- a short, two-sentence summary of the manuscript (not more than 35 words).
- two to four short bullet points highlighting the key findings of your study.
- a schematic summary figure (in jpeg or tiff format with the exact width of 550 pixels and a height of not more than 400 pixels) that can be used as a visual synopsis on our website.

I look forward to seeing the final revised version of your manuscript when it is ready. Please let me know if you have questions regarding the revision.

Achim Breiling
Editor
EMBO Reports

Referee #1:

I have reviewed this work before. The relatively weak point from the previous version is the connection of RGI3 with FLS2. During the revision, the authors have strengthened this part and addressed my other minor concerns.

Referee #2:

I appreciate very much the revisions made by the authors. There are some points to be addressed.

Major points:
1) Although GLV-RGI-mediated control of FLS2 abundance is stressed in the Abstract, the data suggest that EFR also undergoes this regulation. It is important and in my view feasible for the authors to determine whether GLV2 also increases EFR abundance.
In the Abstract, it is better to reflect this new insight. In the Discussion, it is more interesting to note that FLS2 and EFR are both under the similar control, despite their divergence in ERQC-mediated receptor folding control. elf18/EFR needs to be mentioned in the Introduction.

2) In Figure 5C, it is important to note that BAK1 is free from the flg22-induced RGI3-FLS2 complex. This implies that a BAK1-free FLS2 pool is preferentially associated with RGI3 in response to flg22. Thoughtful discussion is needed in L344-345, when proposing the notion that RGi3 is recruited to a flg22-activated FLS2 complex.

Detailed comments:

In the Abstract or Introduction, some explanations are needed for the definition of phytocytokines or in what sense they are analogous to metazoan cytokines. Cytokines are released to regulate the behavior of other cells, in particular immune cells, in metazoans. "Extracellular release" and "cell-to-cell signaling" need to be mentioned when introducing the term "phytocytokines".

L42, it is not clear what "posttranscriptional FLS2 abundance" means.

L73, Peps, PIPs and SCOOPs are implicated in "positive feedback" loops but not in "feed-forward" loops.

L100 and L210, RGI1-5 need to be spelled RGI1-RGI5.

In Fig 1B, what is the possible difference in plant size (growth) between WT and 35S::GLV1/GLV2 plants, in the absence of flg22 application or bacterial inoculation? It is important to state GLV1/GLV2 effects on growth-related processes under the tested conditions.

In Fig EV1C, is there statistical significance between WT and 35S::GLV2? Asterisks needed for all differentially regulated samples of statistical significance, throughout all figures.

L173-174, there was not statistical significance. The results need to be described that there was not a detectable increase in susceptibility.

GLV2 peptide rescue of CRISPR glv phenotypes is integral to the mutant analyses. Fig EV2A and 2B need to be shown in Figure 1. Fig EV2C results are also intriguing, and deserve being shown in Figure 1 as well.

L190, "classical" seems not appropriate.

Why in Figure 5A is there not an increase in FLS2-GFP accumulation in response to GLV2?

L276, both flg22 and GLV2 treatments.

L319, the authors conclude that RGI3 is a GLV2 receptor in leaves. What is RGI3 expression in leaves and other tissues? Some information appreciated.

L330, Pep.

L370-371, it is not clear what the authors wish to say.

The Discussion seems to have a bit too much less-relevant or speculative contents, but lacks some important points raised above. It should be more concise.

Response to referee comments

Referee #1:

I have reviewed this work before. The relatively weak point from the previous version is the connection of RGI3 with FLS2. During the revision, the authors have strengthened this part and addressed my other minor concerns.

Referee #2:

I appreciate very much the revisions made by the authors. There are some points to be addressed.

Major points:

1) Although GLV-RGI-mediated control of FLS2 abundance is stressed in the Abstract, the data suggest that EFR also undergoes this regulation. It is important and in my view feasible for the authors to determine whether GLV2 also increases EFR abundance.
In the Abstract, it is better to reflect this new insight. In the Discussion, it is more interesting to note that FLS2 and EFR are both under the similar control, despite their divergence in ERQC-mediated receptor folding control. elf18/EFR needs to be mentioned in the Introduction.

Response: Thank you very much for suggesting this experiment. Indeed, we noticed that EFR-GFP levels in an *efr* pEFR::EFR-GFP line were also increased after treatment with GLV2 (new Fig. EV4A). We also tested whether *EFR* transcript levels are increased after GLV2 treatment. This is not the case (new Fig. EV4B), suggesting that GLV2 regulates EFR abundance in a similar mechanistic way as FLS2.
We now introduce EFR and elf18 in the introduction section. Moreover, the abstract has been slightly amended to reflect that GLV2 signaling affects PRR abundance and not only FLS2 levels.
Concerning divergence in ERQC-mediated control of FLS2 and EFR we now added the following sentences to the discussion: “Unlike FLS2, EFR accumulation is dependent on functional endoplasmic reticulum quality control (Saijo et al, 2009; Li et al, 2009; Nekrasov et al, 2009) and we cannot exclude that GLV2-RGI3 may also modulate this regulatory pathway. However, this seems less likely as EFR and FLS2 are regulated by GLV2-RGI3 in a similar way.”

2) In Figure 5C, it is important to note that BAK1 is free from the flg22-induced RGI3-FLS2 complex. This implies that a BAK1-free FLS2 pool is preferentially associated with RGI3 in response to flg22. Thoughtful discussion is needed in L344-345, when proposing the notion that RGi3 is recruited to a flg22-activated FLS2 complex.

Response: It is indeed true that we did not see BAK1 co-immunoprecipitating with RGI3 after flg22 treatment. It may be possible that flg22 treatment induces the formation of FLS2-RGI3 complexes free of BAK1. However, the absence of the BAK1 band could also be explained by the experimental setup and western blot detection limits.
The absence of the BAK1 band after flg22 treatment can be interpreted that BAK1 will only be present in a fraction of FLS2 and of this fraction only a subfraction will be recruited to RGI3 which makes this indirect association difficult to visualize by western blot. As an illustration you can see below an uncropped blot where the exact same exposure time is shown for input (left) and IP (right).

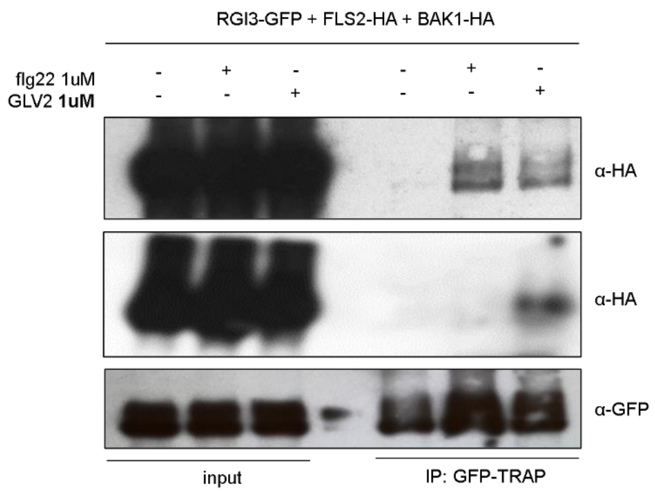

You can see that band intensities for RGI3-GFP are comparable between input and IP, while band intensities for FLS2-HA and BAK1-HA are strongly reduced in the IP compared to the input. Another similar factor of dilution is expected in flg22 treated samples for BAK1 which would be indirectly recruited to RGI3 via FLS2. I hope this illustrates that it is difficult to obtain RGI3-FLS2-BAK1 complexes in this western blot experiment and it does not necessarily indicate that RGI3 is recruited to a BAK1-free pool of FLS2.

Nevertheless, we agree that this point should be discussed appropriately. Thus, we added the following sentences to the discussion section:

“RGI3 did not co-immunoprecipitate BAK1 after flg22 treatment which raises the question whether RGI3 may preferentially associate with a BAK1-free pool of FLS2. However, this may also be explained by detection limitations as BAK1 would only indirectly associate with RGI3 via FLS2 in this experimental setup. Future studies need to determine if RGI3 forms tripartite complexes with PRRs and BAK1 in an activation status-dependent manner.”

Detailed comments:

In the Abstract or Introduction, some explanations are needed for the definition of phytocytokines or in what sense they are analogous to metazoan cytokines. Cytokines are released to regulate the behavior of other cells, in particular immune cells, in metazoans. "Extracellular release" and "cell-to-cell signaling" need to be mentioned when introducing the term "phytocytokines".

Response: We modified the phytocytokine description in the Introduction in the following way: “Research in recent years has identified several classes of such immunomodulatory peptides. These are secreted and modulate cell-to-cell signaling. They are functionally analogous to metazoan cytokines and thus referred to as phytocytokines (Gust et al, 2017).

L42, it is not clear what "posttranscriptional FLS2 abundance" means.

Response: We removed posttranscriptional from the abstract and replaced it with “GLV2-RGI signaling promotes PRR abundance independent of transcriptional regulation…”

L73, Peps, PIPs and SCOOPs are implicated in "positive feedback" loops but not in "feed-forward" loops.

Response: Thank you for your comment and we modified this text section accordingly.

L100 and L210, RGI1-5 need to be spelled RGI1-RGI5.

Response: We corrected this throughout and modified the text accordingly.

In Fig 1B, what is the possible difference in plant size (growth) between WT and 35S::GLV1/GLV2 plants, in the absence of flg22 application or bacterial inoculation? It is important to state GLV1/GLV2 effects on growth-related processes under the tested conditions.

Response: *GLV1* and *GLV2* overexpression lines show altered root gravitropism but no alterations in overall seedling size, as shown in Fig. 1A of Whitford et al., Developmental Cell 2012. Grown under our conditions, *GLV1* and GLV2 *overexpression* lines are slightly smaller compared to the wild type but also do not show overall changes in morphology. Plant Pictures of adult plants were added as the new Fig. EV1 C. Furthermore, we added the following sentences after describing phenotypic results for the *GLV1* and *GLV2* overexpression lines: "*GLV1* and *GLV2* overexpression lines show disturbed root gravitropism and unaltered seedling size (Whitford et al, 2012). Grown under our conditions we observed slightly reduced leaf size compared to the wild type but no overall change in shoot morphology (Fig. EV 1C)."
In addition, we added pictures of the CRISPR *glv* mutant in Fig. EV 1C. We added a sentence before describing phenotypic results of CRISPR *glv*: "Also, grown under our conditions the CRISPR *glv* mutant did not show changes in adult plant morphology (Fig. EV 1C)".

In Fig EV1C, is there statistical significance between WT and 35S::GLV2? Asterisks needed for all differentially regulated samples of statistical significance, throughout all figures.

Response: Indeed, WT and 35S::*GLV2* are statistically different from each other. We apologize for omitting to include statistical analysis for this figure, which has now been modified in the figure, as well as the legend. Furthermore, we slightly rearranged figures and previous Fig. EV 1C is now Fig. EV 2A.

L173-174, there was not statistical significance. The results need to be described that there was not a detectable increase in susceptibility.

Response: We modified the text accordingly and now clearly state that we did not observe increased susceptibility for *amiRglv1 glv2-1* compared to the WT.

GLV2 peptide rescue of CRISPR glv phenotypes is integral to the mutant analyses. Fig EV2A and 2B need to be shown in Figure 1. Fig EV2C results are also intriguing, and deserve being shown in Figure 1 as well.

Response: Thank you for your suggestion. We moved Figure EV2A and EV2B to Figure 1 (new panels Fig. 1F and 1G). However, we kept the GLV2^{-S} SGI data in the expanded view as Fig. EV 2E.

L190, "classical" seems not appropriate.

Response: We removed "classical" as suggested.

Why in Figure 5A is there not an increase in FLS2-GFP accumulation in response to GLV2?

Response: We only observed FLS2 increase in response to GLV2 after prolonged treatment. Depicted in Fig. 6 is 24h GLV2 treatment. We did not expect any differences upon short-term treatment (10 minutes) as performed in Fig. 5A.

L276, both flg22 and GLV2 treatments.

Response: This line describes that GLV2 treatment for 24 hours or GLV2 precursor overexpression resulted in increased FLS2 protein levels. We don't know what the reviewer means as there was no flg22 treatment done in this experiment.

L319, the authors conclude that RGI3 is a GLV2 receptor in leaves. What is RGI3 expression in leaves and other tissues? Some information appreciated.

Response: *RGI3* is expressed in leaves, as shown by semi qRT PCR in Fig. EV3A. Also, this sentence is part of the manuscript: "We confirmed that *RGI3* is expressed in leaves from 6-week-old plants by semi-quantitative reverse transcription PCR (Fig EV3A)."

L330, Pep.

Response: We decided to stick with the nomenclature PEP throughout the manuscript, rather than Pep.

L370-371, it is not clear what the authors wish to say.

Response: This section of the discussion was intended to discuss how GLV-RGI pathway components may contribute to the regulation of PTI. We agree that this part of the discussion may have been a bit too speculative and we thus removed it.

The Discussion seems to have a bit too much less-relevant or speculative contents, but lacks some important points raised above. It should be more concise.

Response: We now condensed the discussion to focus on the more important points.

Dear Dr. Stegmann,

Thank you for the submission of your revised manuscript to our editorial offices. I have now received the report from the referee that was asked to re-evaluate your study, you will find below. As you will see, the referee now fully supports the publication of your study. However, s/he has a final suggestion to improve the manuscript, I ask you to incorporate in a further revised version of the manuscript.

Moreover, I have these editorial requests I also ask you to address:

- Please remove the bullet points and the two-sentence summary from the main manuscript text. I have saved this separately.

- Please completely delete text throught the manuscript presently shown in strikethrough. The typesetter might get confused.

- We updated our journal's competing interests policy in January 2022 and request authors to consider both actual and perceived competing interests. Please review the policy https://www.embopress.org/competing-interests and update your competing interests if necessary. Please name this section 'Disclosure and Competing Interests Statement'.

- In the figure legends, each legend is followed by the sentence 'Data information: Similar results were obtained in at least 3 independent experiments.' This seems in most cases redundant, as details about replicates are given for each panel in the legend. Please remove those sentences if redundant.

- Please add the manuscript title to the Appendix file (e.g. 'Appendix for ...').

Please let me know if you have questions regarding the revision. Please upload the final files via our submission system.

Best,

Achim Breiling
Senior Editor
EMBO Reports

Referee #2:

I am happy with the revisions, and strongly recommend this work be published.
One point to make is, that the data only support the authors' claim in PRRs of BAK1-dependent LRR ectodomain class. Although the manuscript title is good in the present form, this point needs to be mentioned somewhere in the main text.

The authors have addressed all minor editorial requests.

3rd Revision - Editorial Decision 7th Feb 2022

Dr. Martin Stegmann
Phytopathology, School of Life Sciences Weihenstephan, Technical University of Munich, 85354 Freising, Germany
Phytopathology
Emil-Ramann-Straße 2
Freising, Deutschland 85354
Germany

Dear Dr. Stegmann,

I am very pleased to accept your manuscript for publication in the next available issue of EMBO reports. Thank you for your contribution to our journal.

At the end of this email I include important information about how to proceed. Please ensure that you take the time to read the information and complete and return the necessary forms to allow us to publish your manuscript as quickly as possible.

As part of the EMBO publication's Transparent Editorial Process, EMBO reports publishes online a Review Process File to accompany accepted manuscripts. As you are aware, this File will be published in conjunction with your paper and will include the referee reports, your point-by-point response and all pertinent correspondence relating to the manuscript.

If you do NOT want this File to be published, please inform the editorial office within 2 days, if you have not done so already, otherwise the File will be published by default [contact: emboreports@embo.org]. If you do opt out, the Review Process File link will point to the following statement: "No Review Process File is available with this article, as the authors have chosen not to make the review process public in this case."

Thank you again for your contribution to EMBO reports and congratulations on a successful publication. Please consider us again in the future for your most exciting work.

Yours sincerely,

Achim Breiling
Editor
EMBO Reports

****

THINGS TO DO NOW:

You will receive proofs by e-mail approximately 2-3 weeks after all relevant files have been sent to our Production Office; you should return your corrections within 2 days of receiving the proofs.

Please inform us if there is likely to be any difficulty in reaching you at the above address at that time. Failure to meet our deadlines may result in a delay of publication, or publication without your corrections.

All further communications concerning your paper should quote reference number EMBOR-2021-53281V4 and be addressed to emboreports@wiley.com.

Should you be planning a Press Release on your article, please get in contact with emboreports@wiley.com as early as possible, in order to coordinate publication and release dates.

EMBO PRESS

YOU MUST COMPLETE ALL CELLS WITH A PINK BACKGROUND ➜

PLEASE NOTE THAT THIS CHECKLIST WILL BE PUBLISHED ALONGSIDE YOUR PAPER

Corresponding Author Name: Martin Stegmann
Journal Submitted to: EMBO reports
Manuscript Number: EMBOR-2021-53281

USEFUL LINKS FOR COMPLETING THIS FORM

http://www.antibodypedia.com
http://1degreebio.org
http://www.equator-network.org/reporting-guidelines/improving-bioscience-research-repor
http://grants.nih.gov/grants/olaw/olaw.htm
http://www.mrc.ac.uk/Ourresearch/Ethicsresearchguidance/Useofanimals/index.htm
http://ClinicalTrials.gov
http://www.consort-statement.org
http://www.consort-statement.org/checklists/view/32-consort/66-title
http://www.equator-network.org/reporting-guidelines/reporting-recommendations-for-tumo
http://datadryad.org
http://figshare.com
http://www.ncbi.nlm.nih.gov/gap
http://www.ebi.ac.uk/ega
http://biomodels.net/
http://biomodels.net/miriam/
http://jjj.biochem.sun.ac.za
https://osp.od.nih.gov/biosafety-biosecurity-and-emerging-biotechnology/
http://www.selectagents.gov/

Reporting Checklist For Life Sciences Articles (Rev. June 2017)

This checklist is used to ensure good reporting standards and to improve the reproducibility of published results. These guidelines are consistent with the Principles and Guidelines for Reporting Preclinical Research issued by the NIH in 2014. Please follow the journal's authorship guidelines in preparing your manuscript.

A- Figures
1. Data
The data shown in figures should satisfy the following conditions:

- ➔ the data were obtained and processed according to the field's best practice and are presented to reflect the results of the experiments in an accurate and unbiased manner.
- ➔ figure panels include only data points, measurements or observations that can be compared to each other in a scientifically meaningful way.
- ➔ graphs include clearly labeled error bars for independent experiments and sample sizes. Unless justified, error bars should not be shown for technical replicates.
- ➔ if n< 5, the individual data points from each experiment should be plotted and any statistical test employed should be justified
- ➔ Source Data should be included to report the data underlying graphs. Please follow the guidelines set out in the author ship guidelines on Data Presentation.

2. Captions

Each figure caption should contain the following information, for each panel where they are relevant:

- ➔ a specification of the experimental system investigated (eg cell line, species name).
- ➔ the assay(s) and method(s) used to carry out the reported observations and measurements
- ➔ an explicit mention of the biological and chemical entity(ies) that are being measured.
- ➔ an explicit mention of the biological and chemical entity(ies) that are altered/varied/perturbed in a controlled manner.

- ➔ the exact sample size (n) for each experimental group/condition, given as a number, not a range;
- ➔ a description of the sample collection allowing the reader to understand whether the samples represent technical or biological replicates (including how many animals, litters, cultures, etc.).
- ➔ a statement of how many times the experiment shown was independently replicated in the laboratory.
- ➔ definitions of statistical methods and measures:
  - common tests, such as t-test (please specify whether paired vs. unpaired), simple χ^2 tests, Wilcoxon and Mann-Whitney tests, can be unambiguously identified by name only, but more complex techniques should be described in the methods section;
  - are tests one-sided or two-sided?
  - are there adjustments for multiple comparisons?
  - exact statistical test results, e.g., P values = x but not P values < x;
  - definition of 'center values' as median or average;
  - definition of error bars as s.d. or s.e.m.

Any descriptions too long for the figure legend should be included in the methods section and/or with the source data.

In the pink boxes below, please ensure that the answers to the following questions are reported in the manuscript itself. Every question should be answered. If the question is not relevant to your research, please write NA (non applicable). We encourage you to include a specific subsection in the methods section for statistics, reagents, animal models and human subjects.

B- Statistics and general methods

Please fill out these boxes ➜ (Do not worry if you cannot see all your text once you press return)

1.a. How was the sample size chosen to ensure adequate power to detect a pre-specified effect size?	Sample size was determined corresponding to standards in the literature. Every expeirment has been repeated at least three times with similar results.
1.b. For animal studies, include a statement about sample size estimate even if no statistical methods were used.	NA
2. Describe inclusion/exclusion criteria if samples or animals were excluded from the analysis. Were the criteria pre-established?	No data was excluded from analysis
3. Were any steps taken to minimize the effects of subjective bias when allocating animals/samples to treatment (e.g. randomization procedure)? If yes, please describe.	Randomization was not relevant.
For animal studies, include a statement about randomization even if no randomization was used.	NA
4.a. Were any steps taken to minimize the effects of subjective bias during group allocation or/and when assessing results (e.g. blinding of the investigator)? If yes please describe.	Blinding was not required for the assays used here.
4.b. For animal studies, include a statement about blinding even if no blinding was done	NA
5. For every figure, are statistical tests justified as appropriate?	Statistical analysis was performed appropriate to the respective data. Details are found in the respective figure legends.
Do the data meet the assumptions of the tests (e.g., normal distribution)? Describe any methods used to assess it.	NA
Is there an estimate of variation within each group of data?	Yes, this is reperesented through standard deviation in data graphs.

Is the variance similar between the groups that are being statistically compared?	Yes

C- Reagents

6. To show that antibodies were profiled for use in the system under study (assay and species), provide a citation, catalog number and/or clone number, supplementary information or reference to an antibody validation profile. e.g., Antibodypedia (see link list at top right), 1DegreeBio (see link list at top right).	α-GFP (mouse monoclonal B-2, sc-9996, Santa Cruz biotechnology), α-FLS2 (rabbit polyclonal, Chinchilla et al., The Plant Cell 2006), α-BAK1 (rabbit polyclonal, Roux et al., Plant Cell 2011), α-mouse IgG-HRP (A0168, Sigma), α-rabbit IgG–HRP (A0454, Sigma).
7. Identify the source of cell lines and report if they were recently authenticated (e.g., by STR profiling) and tested for mycoplasma contamination.	NA

* for all hyperlinks, please see the table at the top right of the document

D- Animal Models

8. Report species, strain, gender, age of animals and genetic modification status where applicable. Please detail housing and husbandry conditions and the source of animals.	NA
9. For experiments involving live vertebrates, include a statement of compliance with ethical regulations and identify the committee(s) approving the experiments.	NA
10. We recommend consulting the ARRIVE guidelines (see link list at top right) (PLoS Biol. 8(6), e1000412, 2010) to ensure that other relevant aspects of animal studies are adequately reported. See author guidelines, under 'Reporting Guidelines'. See also: NIH (see link list at top right) and MRC (see link list at top right) recommendations. Please confirm compliance.	NA

E- Human Subjects

11. Identify the committee(s) approving the study protocol.	NA
12. Include a statement confirming that informed consent was obtained from all subjects and that the experiments conformed to the principles set out in the WMA Declaration of Helsinki and the Department of Health and Human Services Belmont Report.	NA
13. For publication of patient photos, include a statement confirming that consent to publish was obtained.	NA
14. Report any restrictions on the availability (and/or on the use) of human data or samples.	NA
15. Report the clinical trial registration number (at ClinicalTrials.gov or equivalent), where applicable.	NA
16. For phase II and III randomized controlled trials, please refer to the CONSORT flow diagram (see link list at top right) and submit the CONSORT checklist (see link list at top right) with your submission. See author guidelines, under 'Reporting Guidelines'. Please confirm you have submitted this list.	NA
17. For tumor marker prognostic studies, we recommend that you follow the REMARK reporting guidelines (see link list at top right). See author guidelines, under 'Reporting Guidelines'. Please confirm you have followed these guidelines.	NA

F- Data Accessibility

18: Provide a "Data Availability" section at the end of the Materials & Methods, listing the accession codes for data generated in this study and deposited in a public database (e.g. RNA-Seq data: Gene Expression Omnibus GSE39462, Proteomics data: PRIDE PXD000208 etc.) Please refer to our author guidelines for 'Data Deposition'.<br><br>Data deposition in a public repository is mandatory for:<br>a. Protein, DNA and RNA sequences<br>b. Macromolecular structures<br>c. Crystallographic data for small molecules<br>d. Functional genomics data<br>e. Proteomics and molecular interactions	All targeted proteomic raw data and the Skyline analysis file have been deposited to Panorama Public (Sharma et al, 2018) and can be accessed under https://panoramaweb.org/GLV2_peptide.url (ProteomeXchange ID: PXD023855).
19. Deposition is strongly recommended for any datasets that are central and integral to the study; please consider the journal's data policy. If no structured public repository exists for a given data type, we encourage the provision of datasets in the manuscript as a Supplementary Document (see author guidelines under 'Expanded View' or in unstructured repositories such as Dryad (see link list at top right) or Figshare (see link list at top right).	
20. Access to human clinical and genomic datasets should be provided with as few restrictions as possible while respecting ethical obligations to the patients and relevant medical and legal issues. If practically possible and compatible with the individual consent agreement used in the study, such data should be deposited in one of the major public access-controlled repositories such as dbGAP (see link list at top right) or EGA (see link list at top right).	NA
21. Computational models that are central and integral to a study should be shared without restrictions and provided in a machine-readable form. The relevant accession numbers or links should be provided. When possible, standardized format (SBML, CellML) should be used instead of scripts (e.g. MATLAB). Authors are strongly encouraged to follow the MIRIAM guidelines (see link list at top right) and deposit their model in a public database such as Biomodels (see link list at top right) or JWS Online (see link list at top right). If computer source code is provided with the paper, it should be deposited in a public repository or included in supplementary information.	NA

G- Dual use research of concern

22. Could your study fall under dual use research restrictions? Please check biosecurity documents (see link list at top right) and list of select agents and toxins (APHIS/CDC) (see link list at top right). According to our biosecurity guidelines, provide a statement only if it could.	No
